# Community Health Programs Delivered Through Information and Communications Technology in High-Income Countries: Scoping Review

**DOI:** 10.2196/26515

**Published:** 2022-03-09

**Authors:** Hannah Beks, Olivia King, Renee Clapham, Laura Alston, Kristen Glenister, Carol McKinstry, Claire Quilliam, Ian Wellwood, Catherine Williams, Anna Wong Shee

**Affiliations:** 1 School of Medicine Deakin University Geelong Australia; 2 Barwon Health Geelong Australia; 3 St Vincents Health Australia Melbourne Australia; 4 Ballarat Health Services Ballarat Australia; 5 Colac Area Health Colac Australia; 6 Global Obesity Centre Institute for Health Transformation Deakin University Geelong Australia; 7 Department of Rural Health University of Melbourne Wangaratta Australia; 8 Department of Rural Health University of Melbourne Shepparton Australia; 9 La Trobe Rural Health School La Trobe University Bendigo Australia; 10 Faculty of Health Sciences Australian Catholic University Ballarat Australia

**Keywords:** telemedicine, delivery of health care, pandemics, community health services, information and communications technology, mobile phone

## Abstract

**Background:**

The COVID-19 pandemic has required widespread and rapid adoption of information and communications technology (ICT) platforms by health professionals. Transitioning health programs from face-to-face to remote delivery using ICT platforms has introduced new challenges.

**Objective:**

The objective of this review is to scope for ICT-delivered health programs implemented within the community health setting in high-income countries and rapidly disseminate findings to health professionals.

**Methods:**

The Joanna Briggs Institute’s scoping review methodology guided the review of the literature.

**Results:**

The search retrieved 7110 unique citations. Each title and abstract was screened by at least two reviewers, resulting in 399 citations for full-text review. Of these 399 citations, 72 (18%) were included. An additional 27 citations were identified through reviewing the reference lists of the included studies, resulting in 99 citations. Citations examined 83 ICT-delivered programs from 19 high-income countries. Variations in program design, ICT platforms, research design, and outcomes were evident.

**Conclusions:**

Included programs and research were heterogeneous, addressing prevalent chronic diseases. Evidence was retrieved for the effectiveness of nurse and allied health ICT-delivered programs. Findings indicated that outcomes for participants receiving ICT-delivered programs, when compared with participants receiving in-person programs, were either equivalent or better. Gaps included a paucity of co-designed programs, qualitative research around group programs, programs for patients and carers, and evaluation of cost-effectiveness. During COVID-19 and beyond, health professionals in the community health setting are encouraged to build on existing knowledge and address evidence gaps by developing and evaluating innovative ICT-delivered programs in collaboration with consumers and carers.

## Introduction

### Background

Health professionals, working across community and acute health care settings, have responded rapidly to the COVID-19 pandemic by adopting information and communications technology (ICT) to continue delivering health programs [1-3]. Internationally, there has been an upward surge in the use of ICT to facilitate videoconferencing and telephone consultations to meet physical distancing requirements [4-6]. In Australia, this shift to telehealth in the community health setting required a temporary restructure to government funding models [7]. COVID-19 has been a catalyst for global adoption and focus on the prioritization of ICT in health, particularly in the community health setting (primary care, ambulatory care, home-based care, and outpatient hospital care) where primary and secondary prevention health programs are delivered [3,8-13].

*Digital health*, *eHealth*, and *telehealth* (including telemedicine) are terms used interchangeably and broadly defined as the use of ICT platforms for the remote delivery of health care to consumers [3,14,15]. Examples include videoconferencing and telephone consultations, web-based platforms, electronic health records, SMS text messaging, and smartphone apps (or mobile health, which can include telemonitoring platforms) [14]. Globally, there is increasing support for the use of ICT platforms to improve the accessibility of health services, particularly for health promotion and disease prevention [14,16]. This is evidenced by a surge in research evaluating the usability and effectiveness of ICT-delivered health [17,18], including programs addressing chronic disease risk factors [19-23], patient education and health literacy [24,25], and chronic disease self-management [18,26-28].

Barriers to the adoption of ICT platforms by health professionals are well documented and include a lack of ICT familiarity, lack of time to implement ICT programs, design and technical concerns, and attitudes toward ICT [29-32]. There has been little scope to address these barriers during the pandemic, where there has been a greater focus on the use of ICT in COVID-19 surveillance [33-35], and delivery of telehealth consultations [3,36]. To support health professionals in transitioning community health programs to remote delivery using ICT during COVID-19, a collaborative group was established between 4 Australian universities and 2 regional health services in April 2020. A review working group was formed, with the purpose of engaging directly with health professionals to understand knowledge gaps regarding program delivery using ICT. During the consultation phase (May to June 2020), health professionals voiced concerns regarding the transition of community health programs (particularly group programs) to an ICT platform and the potential for reduced program effectiveness. Similar concerns have been shared by other health professionals internationally [37].

Approaches to undertaking reviews to inform evidence-based decision-making in health care vary [38]. Engaging stakeholders in the review process is suggested to generate more relevant review findings and enable prompt dissemination into practice [39]. An initial search was undertaken of MEDLINE Ovid, Cochrane Database of Systematic Reviews, Joanna Briggs Institute’s (JBI) Evidence Synthesis, and PROSPERO for existing reviews (or proposed reviews) examining ICT-delivered health programs implemented in the community health care setting in high-income countries (HIC). No recent reviews were located that mapped the evidence for community health ICT-delivered programs, justifying the need for a scoping review [40]. The review was limited to HIC because advanced use of ICT platforms is more likely with similarities in resourcing [14]. Capturing a broad range of ICT platforms across various health disciplines and specialties was important for participating health professionals seeking to innovate and engage consumers in programs. Responding to these needs, researchers and health professionals in the review working group collaborated to develop the review question, objectives, and inclusion and exclusion criteria.

### Review Questions and Objectives

The review question is as follows:

What is the evidence for the development and implementation of health programs delivered through ICT for consumers in the community health care settings in HIC?

The specific review objectives include the following:

1. to scope for evidence examining the development and implementation of ICT-delivered health programs in the community health care setting in HIC,
2. to scope for consumer co-design processes used to develop health programs,
3. to examine strategies to facilitate the sharing of consumer lived experience and peer interaction through an ICT platform, and
4. to scope for any andragogical or pedagogical principles or theories, informing program design.

## Methods

### Overview

This scoping review examined the evidence around ICT-delivered health programs implemented in HIC community health care settings. This review used the JBI’s scoping review methodology [41]. Search terms were developed for the population, concept, and context. The review question, objectives, inclusion and exclusion criteria, and search strategies were developed and documented in advance (Section S1 in Multimedia Appendix 1 [41-141]). The PRISMA-ScR (Preferred Reporting Items for Systematic Reviews and Meta-Analyses extension for Scoping Reviews) was adhered to (Table S1 in Multimedia Appendix 1) [42-141].

### Search Strategy

The JBI 3-step search process was used [142]. A preliminary search was undertaken in Ovid MEDLINE and CINAHL using keywords. A tailored search was then developed for each information source using keywords. For databases, a combination of Boolean operators, truncations, and Medical Subject Headings were used to form search strings (Multimedia Appendix 1). Health librarian assistance was obtained for developing the initial Ovid MEDLINE search strategy and translating searches. Reference lists of included studies were also searched for additional studies.

The databases searched included Ovid MEDLINE, CINAHL (EBSCOhost), Embase (Elsevier), and Cochrane Database of Systematic Reviews (Table S2 in Multimedia Appendix 1). Multiple platforms were searched for gray literature (Table S3 in Multimedia Appendix 1). Database searches were conducted on June 16, 2020. Gray literature searches were conducted between June 15 and 30, 2020.

### Inclusion Criteria and Exclusion Criteria

The literature was selected according to the inclusion and exclusion criteria presented in Table 1). Health programs (excluding infectious disease screening, surveillance, antenatal and postnatal, and postoperative rehabilitation programs) delivered by a health professional using an ICT platform to all populations (including carers and family members) in the community health context of HIC, as defined by the Organization for Economic Co-operation and Development (OECD) [142], were included. All types of literature published from January 1, 2010, to June 16, 2020, were included to capture a broad range of ICT platforms and health programs. Only studies published in English were included because of resource constraints.

**Table 1:** Inclusion and exclusion criteria.

	Inclusion criteria	Exclusion criteria
Population	Health programs delivered for infants, children, young people, and adults, including those delivered for consumers, carers, and family or friends of consumers	No exclusions
Concept	Health programs (interventions, models of health care, and services, including, but not limited to, health education, self-management, health promotion and rehabilitation for secondary prevention of disease) delivered by health professionals (including psychologists, speech therapists, speech pathologists, occupational therapists, physiotherapists, physical therapists, podiatrists, exercise physiologists, dietitians, social workers, audiologists, nurses, and doctors) addressing health conditions including, but not limited to, chronic disease (eg, cardiovascular disease, respiratory disease, diabetes, renal disease, cancer, and mental illness) or risk factors for developing chronic disease including, but not limited to, obesity, physical inactivity, poor health literacy, and alcohol misuse using information and communications technology (eg, mobile health, eHealth, telehealth, web-based interventions, and digital health)	Infectious disease screening and surveillance programs, antenatal and postnatal programs, with the exception of gestational diabetes mellitus and postoperative rehabilitation programs
Context	Health programs implemented in the community health context in high-income countries (according to the Organization for Economic Co-operation and Development criteria), including primary care clinics and hospital outpatient clinics	Programs delivered in low- and middle-income countries

### Study Selection and Data Extraction

Searches were undertaken with the assistance of librarians skilled in systematic reviews. Citations were imported into Covidence (Veritas Health Innovation) for screening. Titles and abstracts were screened independently by at least two reviewers with conflicts resolved through mediation with an independent reviewer. All authors were involved in either screening, resolving conflicts, or both. Authors only resolved conflicts for citations that they did not screen. Full-text review and data extraction was then undertaken. For articles not meeting the inclusion criteria, reasons were noted (Table S4 in Multimedia Appendix 1). Reference lists of the included citations were screened for additional literature. Data extraction was tabulated (Section S1 in Multimedia Appendix 1)**,** and findings were synthesized using a descriptive approach informed by the review objectives [41]**.** Consistent with scoping review methods and to enable rapid dissemination of findings, a quality assessment of the studies was not undertaken [143,144].

## Results

### Overview

**Figure 1:**
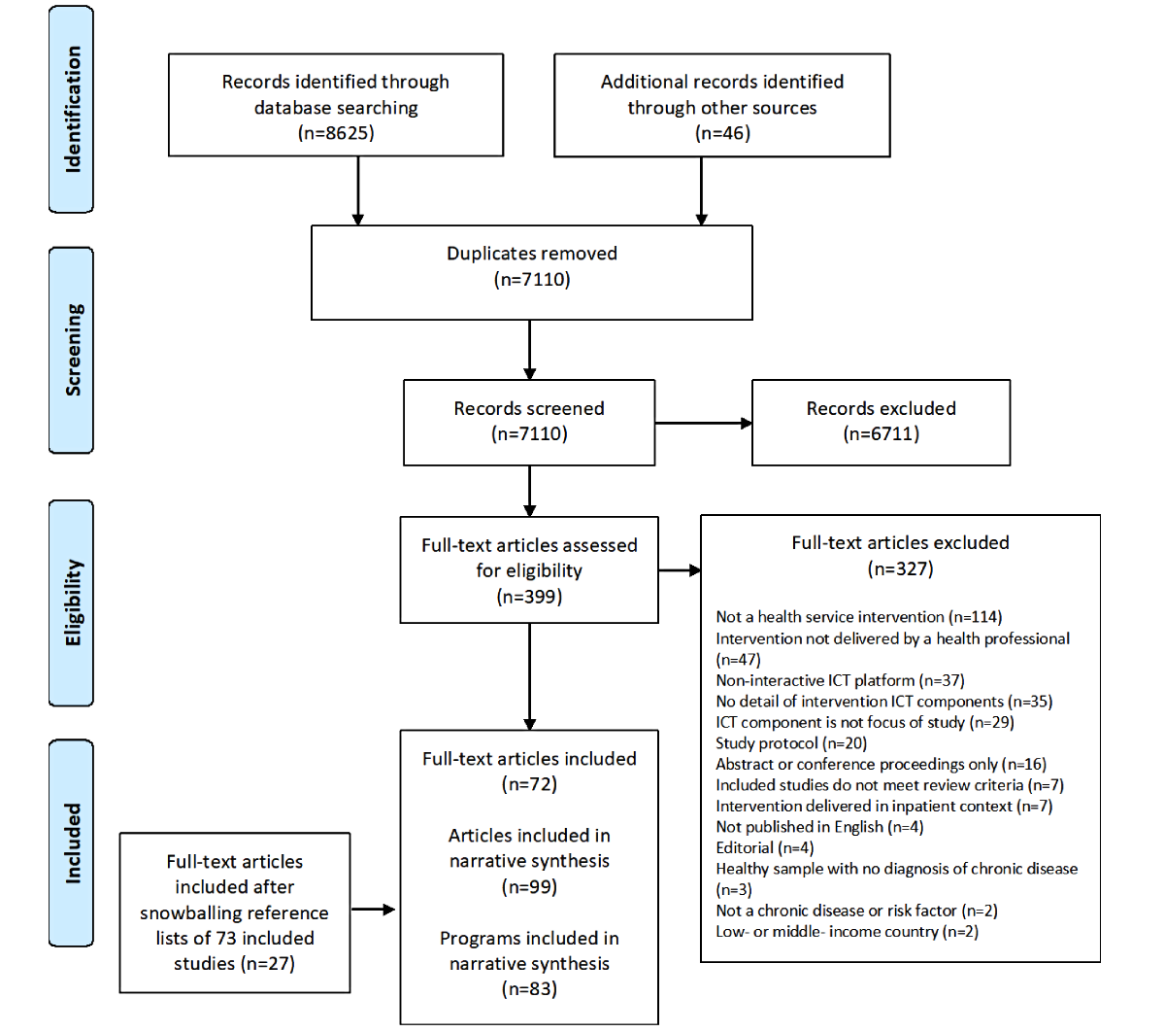
PRISMA (Preferred Reporting Item for Systematic Reviews and Meta-Analyses) flow diagram. ICT: information and communications technology.

Of the 399 citations eligible for full-text screening, 72 (18%) met the inclusion criteria. An additional 27 citations were identified from the reference lists of the included citations, resulting in 99 citations examining 83 programs (Figure 1). Reasons for exclusion were provided (Table S4 in Multimedia Appendix 1).

### Heterogeneity of Programs Using ICT Platforms

The included health programs (n=83) were heterogeneous in design and use of ICT platforms, addressing a variety of chronic diseases (cancer, 3/83, 4%; cardiovascular disease [CVD], 12/83, 14%; diabetes [including gestational diabetes], 30/83, 36%; chronic obstructive pulmonary disease [COPD], 14/83, 17%; other chronic diseases, 11/83, 13%; and chronic pain, 2/83, 2%) and risk factors for developing chronic disease (11/83, 13%; Table S5 in Multimedia Appendix 1). The most frequently used ICT platform for program delivery was the telephone (24/83, 29%) and then internet-based platforms (21/83, 25%), telehealth (telemonitoring; 15/83, 18%), and videoconferencing (11/83, 13%). Some programs used a combination of ICT: telephone and internet-based platforms (1/83, 1%); telephone and mobile apps (2/83, 2%); telemonitoring and an internet-based platform (6/83, 7%); and telehealth (telemonitoring), videoconferencing, and telephone (2/83, 2%). Most programs were delivered by nurses (30/83, 36%) or a multidisciplinary health care team (24/83, 29%), dietitians (8/83, 10%), physiotherapists (7/83, 8%), diabetes educators (4/83, 5%), and psychologists (4/83, 5%). Diverse community health settings were captured where the programs were delivered. Most programs were delivered in outpatient hospital settings (51/83, 61%), followed by home-based settings (12/83, 15%; delivered by other community health organizations that were not primary care practices or hospitals), primary care practices (10/83, 12%), and other community health centers, including multidisciplinary centers (7/83, 8%) and community cancer centers (3/83, 4%).

The included health programs were from 19 OECD HIC. The United States had the highest number of programs (31/83, 37%), followed by Australia (14/83, 17%), Canada (7/83, 8%), Spain (5/83, 6%), the United Kingdom (5/83, 6%), Denmark (4/83, 5%), Norway (3/83, 4%), Italy (3/83, 4%), the Netherlands (2/83, 2%), Belgium (2/83, 2%), Taiwan (2/83, 2%), Greece (2/83, 2%), France (2/83, 2%), Japan (1/83, 1%), Finland (1/83, 1%), Germany (1/83, 1%), South Korea (1/83, 1%), Singapore (1/83, 1%), and Switzerland (1/83, 1%). A total of 2 programs were implemented in >1 country, accounting for 88 sites of program implementation across all included studies [76,86].

### Program Design: Group Programs, Co-design, and Guiding Theories

The programs primarily targeted only patients (76/83, 92%). Fewer programs were for patients and carers (7/83, 8%) and included 2 programs for cancer management [43,44], 1 telemonitoring program for CVD [56], 1 rehabilitation program for acquired brain injury [61], 1 pediatric asthma management program [64,65], 1 coping skills training program for COPD [78], and 1 self-management program for psychological distress [133].

Of the 83 programs, 16 (19%) were either delivered to groups of participants or included a component that involved groups of participants. Of these 16 programs, 5 (31%) targeted diabetes education, self-management, and behavior change coaching [101,102,108,116,125]; 4 (25%) programs were CVD rehabilitation (secondary prevention) or counseling programs [46,47,52,53,55,57]; 4 (25%) addressed risk factors for chronic disease through education and behavior change coaching [131,132,136,141]; 1 (6%) involved group cognitive behavioral therapy (CBT) for participants experiencing insomnia [66]; 1 (6%) involved pharmacist-led group education for hepatitis C [73]; and 1 (6%) involved group education for osteoarthritis [70].

No studies included strategies to facilitate the sharing of consumer lived experience and peer interaction in group ICT-delivered programs. A qualitative study evaluating 1 group program (CVD rehabilitation program) reported that participants engaged in group sessions but did not provide information regarding participants’ experiences [46]. There was limited information of any co-design processes used with consumers or participants to develop programs. Only 2 studies investigating 2 different programs mentioned collaboration with consumers or community organizations to develop interventions; however, no detail about the collaboration was provided [70,119].

None of the studies used specific andragogical or pedagogical principles to inform the delivery of ICT programs to adult or child participants. A total of 12 citations referred explicitly to health behavior theories that informed program development or delivery. Constructs of social cognitive theory (SCT) were used to inform a diabetes self-management support program (Health Education Access Through Information Technology and Utilization Program) [123], a diabetes telemedicine program [111], a pedometer-based intervention for the secondary prevention of CVD [50], a telephone-based Living Well with Diabetes program [104], a telephone-based symptom management program for people with lung cancer and their carers [76], and a telephone-based health coaching program for the secondary prevention of CVD [58,59]. Strategies were implemented to optimize program participation and adherence by promoting SCT constructs (eg, self-efficacy). Examples of strategies included supporting participants to engage in goal setting (eg, related to physical activity) [50], encouraging participants to seek support and rewarding achievements [104], and equipping participants with skills (through cognitive restructuring, problem solving, or self-soothing) to enhance self-efficacy [76].

Other theories included self-determination theory, which informed the development of a telephone-based coaching program targeting physical activity and quality of life for inactive adults through self-management [132]. Using self-determination theory as a conceptual framework, the program integrated motivational interviewing and CBT approaches to coaching [132]. The chronic care model developed by Wagner et al [145] and the transtheoretical model [146] were also used to guide a diabetes self-management education program [119], enabling self-management education and management goals to be provided and set specifically for the stage of change participants were at. The transtheoretical model was also used to inform the content and delivery of pediatric asthma management programs delivered to children and their carers [64,65] and a telehealth diabetes self-management program, along with the health belief model [102].

### Research Evidence: Study Designs, Findings, and Limitations

Heterogeneity was evident in the research design of included citations (n=99) when evaluating the effectiveness, feasibility, or acceptability of the included programs (n=83; Table S6 in Multimedia Appendix 1). Most studies used a randomized controlled trial (RCT) design (58/99, 59%), followed by a single cohort study design (12/99, 12%), a cohort study with 2 or more groups (7/99, 7%), a qualitative design (5/99, 5%), an economic evaluation of an RCT (4/99, 4%), a mixed methods study design (3/99, 3%), or a survey design (2/99, 2%). The remaining citations used other non-RCTs or experimental study designs (8/99, 8%).

Primary and secondary outcomes, and approaches to measuring outcomes (eg, use of validated questionnaires or devices) varied between studies and conditions (Table S6 in Multimedia Appendix 1). For RCTs, the reported effect was categorized as positive (ICT intervention was effective or more effective than control), neutral (effects were equivalent to control), or negative (ICT intervention was not effective or less effective than control) where appropriate, to provide an indication of the effectiveness of programs using ICT platforms. Of the 58 studies able to be categorized, 30 (52%) reported positive effects on the primary and secondary outcomes attributed to the ICT intervention, when compared with the control group, whereas 28 (48%) studies reported a neutral effect. No RCTs reported that outcomes were worse in the ICT intervention group than in the control group. Owing to the heterogeneity of primary and secondary outcome measures and program design, the most frequently reported outcome measures for condition groups used in RCTs are reported in Table 2, with the effects categorized. From the studies included in this table, there was consistency in the findings of RCTs of COPD programs reporting on health service use outcome measures. The effect of programs on the rate of hospitalization of the ICT intervention and control groups were found to be neutral. However, for RCTs of programs using clinical, anthropometric, or physical activity outcome measures, there was a mix of positive and neutral effects. The length of the final follow-up periods in RCTs ranged from 6 weeks to 5 years (with a median follow-up period of 12, IQR 6-15 months).

**Table 2:** Most frequently reported primary outcome measures in included RCTs^a^.

Study		Reported effect and results
**Outcome measure: HbA**_1c_ **^b^ (diabetes programs)**		
	Baron et al [94]	Neutral: Program did not achieve a clinically significant reduction in HbA_1c_.
	Blackberry et al [96]	Neutral: At 18-months follow-up, the effect on HbA_1c_ did not differ between the intervention and control (mean difference 0.2, 95%CI −0.2 to 0.2; *P*=.84).
	Buysse et al [97]	Positive: Both groups received tele-education at different time points (delayed access [control] and immediate access [study group]) and demonstrated an overall significant impact of tele-education on HbA_1c_ reduction (−0.5% control and −0.4% study group, respectively).
	Carter et al [98]	Positive: Patients enrolled in intervention were 4.58 times more likely to achieve an HbA_1c_ target <7%.
	Charpentier et al [99]	Positive: At 6 months, mean HbA_1c_ was lower in the intervention group than in the control group (8.41 vs 9.10, respectively).
	Davis et al [102]	Positive: A significant reduction in HbA_1c_ was found in the intervention group, compared with usual care (9.4 to 8.2 in the intervention group, compared with 8.8 to 8.6 in usual care).
	Fountoulakis et al [107]	Positive: Significant reduction in HbA_1c_ in the intervention group at 3 and 6 months, when compared with that in the control group.
	Greenwood et al [108]	Positive: The intervention group had a statistically significant difference of 0.41 percentage points at 6 months when compared with the control group.
	Klingeman et al [117]	Positive: Average HbA_1c_ reduced by 1.7% in the intervention group, compared with 0.3% in the control group.
	Sood et al [124]	Neutral: No statistically significant differences between the intervention and control groups at 18 months.
	Varney et al [127]	Positive: The intervention group experienced a greater mean change in adjusted HbA_1c_ than the controls between baseline and 12 months; however, this was not sustained.
	Wakefield et al [129]	Neutral: Participants in the intervention group experienced decreased HbA_1c_ during the 6-month intervention period when compared with the control group; however, 6 months after the intervention was withdrawn, the intervention groups were comparable with the control group.
	Weinstock et al [113]	Positive: Intervention was associated with improved HbA_1c_ over 5 years, when compared with control.
	Wild et al [110]	Positive: Clinically and statistically significant improvements were observed in the intervention group at 9 months, when compared with the control group.
**Outcome measure: rate of hospitalization (COPD^c^ programs)**		
	Antoniades et al [75]	Neutral: No significant difference between the intervention and control groups at 12 months.
	Blumenthal et al [78]	Neutral: No significant difference between the intervention and control groups up to 4.4 years follow-up
	Fairbrother et al [84]	Neutral: No significant difference between the intervention and control groups at 12 months.
	Pinnock et al [85]	Neutral: No significant difference between the intervention and control groups at 12 months.
	Kessler et al [86]	Neutral: No significant difference between the intervention and control groups at 12 months.
	Tabak et al [89]	Neutral: No significant difference between the intervention and control groups at 2 months.
**Outcome measure: PA^d^ or capacity (cardiovascular disease programs)**		
	Lear et al [47]	Positive: Intervention group participants who received support from a health professional through an internet-based platform had a greater increase in maximal time on the treadmill by 45.7 seconds (95% CI 1.04-90.48) compared with the usual care group over the 16 months (*P*=.045).
	Furber et al [50]	Positive: After the 6-week intervention, improvements in total PA time, total PA sessions, walking time, and walking sessions were all significantly greater in the intervention group who received telephone support than in the control who received 2 education pamphlets and no support via telephone.
	Hawkes et al [59]	Neutral: No significant difference between the PA of participants in the intervention and control groups at 6 months follow-up.
	Hwang et al [52,53]	Neutral: No difference was found between the PA of participants receiving the telerehabilitation intervention when compared with the control group who received center-based care, and it was less costly than center-based heart failure rehabilitation.
	Nolan et al [57]	Positive: More telehealth participants than control participants reported adherence to exercise and diet after treatment at a 6-month follow-up.
**Outcome measure: weight loss or prevention of weight gained (risk factors for chronic disease programs)**		
	Ferrara et al [135]	Positive: Compared with those receiving usual care, women in the lifestyle intervention had reduced weekly rate of gestational weight gain (mean 0.26 vs 0.32 kg/week).
	Padwal et al [138]	Neutral: Face to-face or web-based delivery of intensive self-management program was no more effective than the once off provision of educational materials and were more costly.
	Weinstock et al [141]	Positive: Mean percent weight loss at 2-year follow-up was higher for the conference call group than for the individual call group (−5.6% compared with −1.8%).

^a^RCT: randomized controlled trial.

^b^HbA_1c_: glycated hemoglobin A_1c_.

^c^COPD: chronic obstructive pulmonary disease.

^d^PA: physical activity.

Of the 7 studies using qualitative inquiry (including mixed methods studies using qualitative inquiry), 3 (43%) studies examined the attitudes of participants (a videoconferencing education workshop for inflammatory arthritis, a COPD telemonitoring program, and a telemonitoring program for diabetes) [71,88,109], 2 (29%) examined perceptions of a T2DM smartphone app [118,121], 1 (14%) measured the patient experience of being involved in a web-based cardiac rehabilitation program [46], and 1 (14%) examined the perceptions of both patients and health professionals involved with a COPD telemonitoring service [83]. Themes varied but generally related to the accessibility of ICT programs [46] and general participant satisfaction [88]. A study also reported no difference in feedback obtained from participants who attended an in-person program compared with those who attended videoconferencing [71]. Another study reported limitations of using ICT platforms, including frustration with using smartphones [118], whereas other studies reported that technology was acceptable [83,88,109,121].

Studies providing an economic evaluation of an ICT-delivered program, in conjunction with either an RCT [49,52,54,132] or a case-control study [51], supported the potential for the cost-effectiveness of ICT-delivered programs when compared with in-person programs. When examining telerehabilitation for CVD, Hwang et al [52] found the intervention to be as effective and less costly than center-based rehabilitation. Ho et al [51] reported that a telehealth program for CVD was more cost-effective and more likely to prevent hospitalizations than usual care. However, a telemonitoring program for CVD was reportedly not cost-effective because the intervention had higher costs (including equipment costs) than usual care, and no significant difference was found in quality-adjusted life years [54].

Research limitations frequently reported included high attrition rates, small sample sizes (or not statistically powered for outcome measures), and limited external validity. The total attrition rates of RCTs ranged from 1% to 63%, with a median attrition rate of 18% (IQR 10%-25%). Difficulty in recruiting participants was also reported by some researchers. An RCT conducted a survey of why participants declined to participate in the trial and found personal reasons and concerns with technology were frequently cited by respondents [54].

## Discussion

### Principal Findings

This review provides a broad overview of research examining ICT-delivered programs implemented in the community health setting in 19 countries, providing a sample of programs from 24% (19/80) of OECD HIC [144]. The highest proportion of included ICT-delivered programs was implemented in the United States, the country with the highest financial investment in health care (16.9% of gross domestic product in 2018) [147] and a growing investment in digital health [148]. Although this review was limited to programs implemented by OECD HIC, other studies have identified a surge in ICT programs and innovations in low- and middle-income countries [149-151]. Because of the COVID-19 pandemic, it is anticipated that ICT-delivered health programs and innovations will continue to increase as global health care systems are transformed [152].

Included programs and citations were diverse, addressing a range of chronic diseases and risk factors, using a variety of ICT platforms delivered by different health professionals across different community health settings. Programs mostly targeted highly prevalent chronic diseases and risk factors, such as CVD, COPD, diabetes, and obesity or being overweight [153,154], and were delivered in the outpatient hospital setting. The need to facilitate a greater adoption of ICT in other community health settings (eg, primary care practices) has been identified by other international research and is supported by the review findings [155]. Furthermore, there were few self-management and education programs addressing cancer and mental health conditions, other chronic diseases that pose a significant burden on global populations [153]. During the COVID-19 pandemic, the need for improved accessibility to mental health programs has also been identified [156]. There were also few programs implemented for patients and carers. There is an increasing focus on the importance of carer engagement, particularly for dementia care [157] and mental health [158], and research around the role of ICT programs in supporting carers [159].

A high proportion of programs were delivered using the telephone, internet, and telemonitoring. With a surge in the use of mobile health technologies through smartphone apps and other innovations (eg, activity monitoring devices), this finding suggests that the telephone remains an important ICT platform for improving patient accessibility to health professionals, particularly for self-management and behavior change coaching. This is evident by the use of telehealth during COVID-19 in countries such as Australia, where telephone consultations have had a higher uptake in primary care settings compared with videoconferencing delivered via web-based platforms [160]. The usefulness of videoconferencing for delivering group education, behavior change coaching, and self-management programs is also indicated by the review findings. Although this review reports little about the acceptability of ICT-delivered group programs and strategies to facilitate peer interaction, other reviews have found that group programs delivered through videoconferencing have been acceptable and feasible to participants [161]. Future research needs to examine how to facilitate group interaction in ICT programs [162].

Although the included studies had a range of research designs (a finding of another systematic review examining emergent eHealth interventions [163]), the findings from this review supported the effectiveness of nurse-led ICT programs in improving pain associated with cancer [43], improving quality-of-life outcomes and reducing hospital admissions for patients with CVD [56], improving health outcomes for patients with CVD [58,59], and improving quality of life in carers of children with asthma [64,65]. Findings also indicated the effectiveness of ICT-delivered programs by allied health professionals, including a telenutrition program delivered by dietitians [60] and a chronic pain program delivered by physiotherapists [91]. The results from included RCTs comparing participant outcomes of an ICT program to a control group (receiving mostly in-person care) were either equivalent or better for ICT programs. Other reviews examining ICT interventions, such as nurse and allied health, delivered telehealth interventions [164], and electronic CBT [165], also concluded that delivering health interventions through ICT platforms does not lead to poorer health outcomes for patients.

Substantial gaps in research evidence relating to ICT programs delivered in the community health setting by health professionals were identified. There were few co-designed ICT programs (and no documentation of co-design processes) and no reference to specific pedagogical or andragogical educational principles guiding program delivery—gaps identified by other reviews [166,167]. Engaging stakeholders in program development through co-design processes is thought to create programs that are more useful and acceptable to end users [168]. Some programs were developed or guided by theories; however, further research is required to examine whether using theories (eg, SCT) to develop and guide programs results in better outcomes for participants [169]. Few studies have examined participant acceptability, experience, and perceptions of ICT programs through qualitative inquiry. However, qualitative findings resonate with other reviews that have found that participants are generally satisfied with telehealth [170]. Findings indicate that there is a need for greater consumer engagement in the process of developing ICT programs and evaluating effectiveness [171]. There is also a need for more economic evaluations of ICT programs delivered in the community health setting, which is also lacking in broader health services research [172,173].

Engaging with health professionals to understand knowledge gaps regarding community health ICT program delivery during COVID-19 and codevelopment of the scoping review question, objectives, and inclusion and exclusion criteria are strengths of this review. A summary of review findings was rapidly disseminated to health professionals involved, and findings were discussed during a short webinar. The limitations of the review include only a brief search of international gray literature due to the need to rapidly disseminate findings to health professionals. Undertaking a more thorough search of the international gray literature could have minimized publication bias. There is potential that relevant citations were not included in the review owing to this constraint. Despite this, every effort was made to review the reference lists of included citations for additional studies. Studies published in a language other than English were not captured by this review owing to resource constraints.

### Conclusions

This review identified heterogeneity in available evidence examining ICT-delivered programs in community health settings in HIC. There is promising evidence for the effectiveness of nurse and allied health delivered ICT programs. From RCTs, outcomes for participants receiving ICT programs, compared with those receiving in-person programs, were either equivalent or better. Gaps identified included a paucity of co-designed programs; qualitative research relating to consumer acceptability, experience, and interactions in group programs; and cost-effectiveness of ICT programs and programs targeting patients and carers. It is expected that because of COVID-19, there will be a surge in the innovation, development, and evaluation of community health programs delivered using ICT platforms, providing an opportunity for health professionals and researchers to build on existing knowledge and address evidence gaps.

## Appendix

Multimedia Appendix 1 Included studies characteristics, search strategies, protocol, excluded studies, and PRISMA-ScR (Preferred Reporting Items for Systematic Reviews and Meta-Analyses extension for Scoping Reviews) checklist.

## Abbreviations

CBT: cognitive behavioral therapy
COPD: chronic obstructive pulmonary disease
CVD: cardiovascular disease
HIC: high-income countries
ICT: information and communications technology
JBI: Joanna Briggs Institute
OECD: Organization for Economic Co-operation and Development
PRISMA-ScR: Preferred Reporting Items for Systematic Reviews and Meta-Analyses extension for Scoping Reviews
RCT: randomized controlled trial
SCT: social cognitive theory
