# Supplementary material for: Community Health Programs Delivered Through Information and Communications Technology in High-Income Countries: Scoping Review
Source: J Med Internet Res. 2022 Mar 9;24(3):e26515. doi: 10.2196/26515 (PMC8943572; doi:10.2196/26515)
Supplement: Multimedia Appendix 1 [file jmir_v24i3e26515_app1.docx]

**Section S1 - Scoping Review protocol (v.2 2 June 2020)**

The review questions, objectives and inclusion/exclusion criteria were developed through a series of workshops with clinicians and health service academics during the early phase of the COVID-19 lockdown period. These formed a protocol for undertaking a scoping review using the Joanna Briggs Institute’s Methodology for Scoping Reviews^41^.

**Review question**

*What is the evidence around the development and implementation of health programs delivered through information and communications technology for consumers in the community health care setting in high-income countries?*

**Review objectives**

(1) Scope the literature for evidence examining the development and implementation of health programs delivered through information and communications technology (ICT) for consumers in the community health care setting in high-income countries,

(2) Scope for any co-design processes used with consumers to develop multi-disciplinary health programs,

(3) Examine any strategies to facilitate the sharing of consumer lived experience and peer interaction through an ICT platform, and

(4) Scope for any andragogy or pedagogical principles, including theories informing health program design including building rapport between consumers and clinicians.

**Table 1. Inclusion and exclusion criteria**

|  | **Inclusion criteria** | | **Exclusion criteria** |
| --- | --- | --- | --- |
| **Population** | Health programs delivered for infants, children, young people and adults, including those delivered for consumers, carers and family/friends of consumers. | No exclusions. | |
| **Concept** | Health programs (including, but not limited to, health education, self-management, health promotion and rehabilitation for secondary prevention of disease) delivered by health professionals (including psychologists, speech therapists, speech pathologists, occupational therapists, physiotherapists, physical therapists, podiatrists, exercise physiologists, dietitians, social workers, audiologists, nurses and doctors) addressing health conditions including , but not limited to, chronic disease (e.g. cardiovascular disease, respiratory disease, diabetes, renal disease, cancer, mental illness) and/or risk factors for developing chronic disease including , but not limited to, obesity, physical inactivity, poor health literacy and alcohol misuse using information communications technology (e.g. mHealth , eHealth, telehealth , virtual care, web based interventions, digital health). | Exclude infectious disease screening and surveillance programs, antenatal/postnatal programs, with the exception of gestational diabetes mellitus and post-operative rehabilitation programs. | |
| **Context** | Health programs implemented in the community health context in high income countries including primary care clinics and hospital outpatient clinics with findings published since 1 January 2010. | Exclude programs delivered in low and middle-income countries. | |

The databases to be searched include: Ovid MEDLINE, CINAHL (EBSCOhost), Embase (Elsevier) and Cochrane Database of Systematic Reviews. Multiple platforms are to be searched for unpublished studies and grey literature

**Data extraction**

Data extraction is to be presented in a table categorized under the following headings; author, date of publication, target disease and/or chronic disease risk factor, target group, design, country, location, intervention type, intervention description, intervention development with consumer involvement, discipline deliver program, Information Communication Technology (ICT) platform used, sample size, attrition rates, mean age years (standard deviation) or years (range), female n= (%), primary outcome, secondary outcome, key results, conclusions/implications for practice and reported limitations.

**Table 2. Search strategy: Ovid MEDLINE**

| **Search string** | **Query** |
| --- | --- |
| 1 | exp Health Education/ |
| 2 | (program* adj2 health).kf,tw. |
| 3 | ((patient or health) adj2 education).kf,tw. |
| 4 | or/1-3 |
| 5 | Outpatients/ |
| 6 | (Home care adj3 (service or delivery)).kf,tw. |
| 7 | community health.kf,tw. |
| 8 | (outpatient* or out-patient*).kf,tw. |
| 9 | (ambulatory care or ambulatory health service* or ambulatory health care service* or ambulatory primary health service*).kf,tw. |
| 10 | exp Community Health Services/ |
| 11 | community health services/ or community health nursing/ or community mental health services/ or maternal health services/ or health services for the aged/ or health services, indigenous/ |
| 12 | exp Health Services/ |
| 13 | exp Primary Health Care/ |
| 14 | exp Home Care Services/ |
| 15 | exp Ambulatory Care/ |
| 16 | or/5-15 |
| 17 | Smartphone/ |
| 18 | exp Cell Phone/ |
| 19 | telecommunications/ |
| 20 | webcasts as topic/ |
| 21 | exp telemedicine/ |
| 22 | ((information and communications technolog*) or ICT* or telecommunication*).kf,tw. |
| 23 | (app or software or computer program*).kf,tw. |
| 24 | (mobile* or smartphone or smart phone or cell* phone).kf,tw. |
| 25 | (tablet or device or iPad or iPhone or android or computer or PC or laptop).kf,tw. |
| 26 | (telehealth or ehealth or e-health or electronic health or mhealth or m-health or mobile health or m-learning or e-learning or digital health or DHI or virtual care).kf,tw. |
| 27 | (internet-based or web-based).kf,tw. |
| 28 | (webcast or webinar* or podcast or youtube or vimeo or multi-media or multimedia).kf,tw. |
| 29 | (video or video-based or video recording).kf,tw. |
| 30 | or/17-29 |
| 31 | (New Zealand* or Northland or Auckland or Waikato or Gisborne or Hawkes Bay or Taranaki or Whanganui or Manawatu or Wellington or Marlborough or Nelson or Tasman or West Coast or Canterbury or Christchurch or Dunedin or Otago or Southland).kf,tw. |
| 32 | (Australia* or Tasmania* or Victoria* or New South Wales or Queensland or Northern Territor* or Western Australia* or South Australia*).kf,tw. |
| 33 | exp Australia/ |
| 34 | exp Austria-Hungary/ or exp Austria/ |
| 35 | exp Singapore/ |
| 36 | exp Belgium/ |
| 37 | exp Poland/ |
| 38 | exp Croatia/ |
| 39 | exp Czech Republic/ |
| 40 | exp Canada/ |
| 41 | exp Switzerland/ |
| 42 | exp Germany, East/ or exp Germany/ or exp Germany, West/ |
| 43 | exp Spain/ |
| 44 | exp France/ |
| 45 | exp Greece/ |
| 46 | exp United Kingdom/ |
| 47 | exp Scotland/ |
| 48 | exp Northern Ireland/ or exp Ireland/ |
| 49 | exp Italy/ |
| 50 | exp Japan/ |
| 51 | exp Hong Kong/ |
| 52 | exp Norway/ |
| 53 | exp Netherlands/ |
| 54 | exp Sweden/ |
| 55 | exp "Scandinavian and Nordic Countries"/ |
| 56 | exp New Zealand/ |
| 57 | exp United States/ |
| 58 | Alaska/ |
| 59 | or/31-58 |
| 60 | and/4,16,30,59 |
| 61 | Limit 60 to yr=“2010-Current” |

**Table S1 - Preferred Reporting Items for Systematic reviews and Meta-Analyses extension for Scoping Reviews (PRISMA-ScR) Checklist**

| **SECTION** | **ITEM** | **PRISMA-ScR CHECKLIST ITEM** | **REPORTED ON PAGE #** |
| --- | --- | --- | --- |
| **TITLE** | | | |
| Title | 1 | Identify the report as a scoping review. | 2 |
| **ABSTRACT** | | | |
| Structured summary | 2 | Provide a structured summary that includes (as applicable): background, objectives, eligibility criteria, sources of evidence, charting methods, results, and conclusions that relate to the review questions and objectives. | 2 |
| **INTRODUCTION** | | | |
| Rationale | 3 | Describe the rationale for the review in the context of what is already known. Explain why the review questions/objectives lend themselves to a scoping review approach. | 3-4 |
| Objectives | 4 | Provide an explicit statement of the questions and objectives being addressed with reference to their key elements (e.g., population or participants, concepts, and context) or other relevant key elements used to conceptualize the review questions and/or objectives. | 4 |
| **METHODS** | | | |
| Protocol and registration | 5 | Indicate whether a review protocol exists; state if and where it can be accessed (e.g., a Web address); and if available, provide registration information, including the registration number. | 5 (Additional file 1) |
| Eligibility criteria | 6 | Specify characteristics of the sources of evidence used as eligibility criteria (e.g., years considered, language, and publication status), and provide a rationale. | 5-6 (Additional file 1) |
| Information sources* | 7 | Describe all information sources in the search (e.g., databases with dates of coverage and contact with authors to identify additional sources), as well as the date the most recent search was executed. | 4-5 (Additional file 3, Additional file 4) |
| Search | 8 | Present the full electronic search strategy for at least 1 database, including any limits used, such that it could be repeated. | Additional file 3 |
| Selection of sources of evidence† | 9 | State the process for selecting sources of evidence (i.e., screening and eligibility) included in the scoping review. | 5-7 |
| Data charting process‡ | 10 | Describe the methods of charting data from the included sources of evidence (e.g., calibrated forms or forms that have been tested by the team before their use, and whether data charting was done independently or in duplicate) and any processes for obtaining and confirming data from investigators. | 5-7 |
| Data items | 11 | List and define all variables for which data were sought and any assumptions and simplifications made. | 5-7 (Additional file 1) |
| Critical appraisal of individual sources of evidence§ | 12 | If done, provide a rationale for conducting a critical appraisal of included sources of evidence; describe the methods used and how this information was used in any data synthesis (if appropriate). | N/A |
| Synthesis of results | 13 | Describe the methods of handling and summarizing the data that were charted. | 5-7 |

| **SECTION** | **ITEM** | **PRISMA-ScR CHECKLIST ITEM** | **REPORTED ON PAGE #** |
| --- | --- | --- | --- |
| **RESULTS** | | | |
| Selection of sources of evidence | 14 | Give numbers of sources of evidence screened, assessed for eligibility, and included in the review, with reasons for exclusions at each stage, ideally using a flow diagram. | 7 (Figure 1) |
| Characteristics of sources of evidence | 15 | For each source of evidence, present characteristics for which data were charted and provide the citations. | 7-11 (Table 3, Table 4) |
| Critical appraisal within sources of evidence | 16 | If done, present data on critical appraisal of included sources of evidence (see item 12). | N/A |
| Results of individual sources of evidence | 17 | For each included source of evidence, present the relevant data that were charted that relate to the review questions and objectives. | Table 3, Table 4 |
| Synthesis of results | 18 | Summarize and/or present the charting results as they relate to the review questions and objectives. | 7-11 (Figure 2) |
| **DISCUSSION** | | | |
| Summary of evidence | 19 | Summarize the main results (including an overview of concepts, themes, and types of evidence available), link to the review questions and objectives, and consider the relevance to key groups. | 11-14 |
| Limitations | 20 | Discuss the limitations of the scoping review process. | 15 |
| Conclusions | 21 | Provide a general interpretation of the results with respect to the review questions and objectives, as well as potential implications and/or next steps. | 15 |
| **FUNDING** | | | |
| Funding | 22 | Describe sources of funding for the included sources of evidence, as well as sources of funding for the scoping review. Describe the role of the funders of the scoping review. | 15-16 |

JBI = Joanna Briggs Institute; PRISMA-ScR = Preferred Reporting Items for Systematic reviews and Meta-Analyses extension for Scoping Reviews.

* Where *sources of evidence* (see second footnote) are compiled from, such as bibliographic databases, social media platforms, and Web sites.

† A more inclusive/heterogeneous term used to account for the different types of evidence or data sources (e.g., quantitative and/or qualitative research, expert opinion, and policy documents) that may be eligible in a scoping review as opposed to only studies. This is not to be confused with *information sources* (see first footnote).

‡ The frameworks by Arksey and O’Malley (6) and Levac and colleagues (7) and the JBI guidance (4, 5) refer to the process of data extraction in a scoping review as data charting*.*

§ The process of systematically examining research evidence to assess its validity, results, and relevance before using it to inform a decision. This term is used for items 12 and 19 instead of "risk of bias" (which is more applicable to systematic reviews of interventions) to include and acknowledge the various sources of evidence that may be used in a scoping review (e.g., quantitative and/or qualitative research, expert opinion, and policy document).

*From:* Tricco AC, Lillie E, Zarin W, O'Brien KK, Colquhoun H, Levac D, et al. PRISMA Extension for Scoping Reviews (PRISMAScR): Checklist and Explanation. Ann Intern Med. 2018;169:467–473. [doi: 10.7326/M18-0850.](http://annals.org/aim/fullarticle/2700389/prisma-extension-scoping-reviews-prisma-scr-checklist-explanation)

**Table S2 - Electronic search results and terms**

| Ovid MEDLINE | 3552 |
| --- | --- |
| CINAHL (EBSCOhost) | 2060 |
| Embase (Elsevier) | 1976 |
| Cochrane Central Register of Controlled Trials | 1037 |
| Total | 8625 |
| Duplicate papers | 1514 |
| Total left to screen | 7111 |

Database searches were conducted on the 16 June 2020. Grey literature searches were conducted between 15 June 2020 and 30 June 2020.

Database(s): **Ovid MEDLINE(R) Epub Ahead of Print, In-Process & Other Non-Indexed Citations, Ovid MEDLINE(R) Daily, Ovid MEDLINE and Versions(R)**

Search strategy:

| **Search string** | **Query** |
| --- | --- |
| 1 | exp Health Education/ |
| 2 | (program* adj2 health).kf,tw. |
| 3 | ((patient or health) adj2 education).kf,tw. |
| 4 | or/1-3 |
| 5 | Outpatients/ |
| 6 | (Home care adj3 (service or delivery)).kf,tw. |
| 7 | community health.kf,tw. |
| 8 | (outpatient* or out-patient*).kf,tw. |
| 9 | (ambulatory care or ambulatory health service* or ambulatory health care service* or ambulatory primary health service*).kf,tw. |
| 10 | exp Community Health Services/ |
| 11 | community health services/ or community health nursing/ or community mental health services/ or maternal health services/ or health services for the aged/ or health services, indigenous/ |
| 12 | exp Health Services/ |
| 13 | exp Primary Health Care/ |
| 14 | exp Home Care Services/ |
| 15 | exp Ambulatory Care/ |
| 16 | or/5-15 |
| 17 | Smartphone/ |
| 18 | exp Cell Phone/ |
| 19 | telecommunications/ |
| 20 | webcasts as topic/ |
| 21 | exp telemedicine/ |
| 22 | ((information and communications technolog*) or ICT* or telecommunication*).kf,tw. |
| 23 | (app or software or computer program*).kf,tw. |
| 24 | (mobile* or smartphone or smart phone or cell* phone).kf,tw. |
| 25 | (tablet or device or iPad or iPhone or android or computer or PC or laptop).kf,tw. |
| 26 | (telehealth or ehealth or e-health or electronic health or mhealth or m-health or mobile health or m-learning or e-learning or digital health or DHI or virtual care).kf,tw. |
| 27 | (internet-based or web-based).kf,tw. |
| 28 | (webcast or webinar* or podcast or youtube or vimeo or multi-media or multimedia).kf,tw. |
| 29 | (video or video-based or video recording).kf,tw. |
| 30 | or/17-29 |
| 31 | (New Zealand* or Northland or Auckland or Waikato or Gisborne or Hawkes Bay or Taranaki or Whanganui or Manawatu or Wellington or Marlborough or Nelson or Tasman or West Coast or Canterbury or Christchurch or Dunedin or Otago or Southland).kf,tw. |
| 32 | (Australia* or Tasmania* or Victoria* or New South Wales or Queensland or Northern Territor* or Western Australia* or South Australia*).kf,tw. |
| 33 | exp Australia/ |
| 34 | exp Austria-Hungary/ or exp Austria/ |
| 35 | exp Singapore/ |
| 36 | exp Belgium/ |
| 37 | exp Poland/ |
| 38 | exp Croatia/ |
| 39 | exp Czech Republic/ |
| 40 | exp Canada/ |
| 41 | exp Switzerland/ |
| 42 | exp Germany, East/ or exp Germany/ or exp Germany, West/ |
| 43 | exp Spain/ |
| 44 | exp France/ |
| 45 | exp Greece/ |
| 46 | exp United Kingdom/ |
| 47 | exp Scotland/ |
| 48 | exp Northern Ireland/ or exp Ireland/ |
| 49 | exp Italy/ |
| 50 | exp Japan/ |
| 51 | exp Hong Kong/ |
| 52 | exp Norway/ |
| 53 | exp Netherlands/ |
| 54 | exp Sweden/ |
| 55 | exp "Scandinavian and Nordic Countries"/ |
| 56 | exp New Zealand/ |
| 57 | exp United States/ |
| 58 | Alaska/ |
| 59 | or/31-58 |
| 60 | and/4,16,30,59 |
| 61 | Limit 60 to yr=“2010-Current” |

Database(s): **CINAHL (EBSCOhost)**

Search strategy:

| **Search string** | **Query** |
| --- | --- |
| S1 | (MH “Health Education+”) |
| S2 | TI (program N2 health) OR AB (program N2 health) |
| S3 | TI ((patient or health) N2 education) OR AB ((patient or health) N2 education) |
| S4 | S1 OR S2 OR S3 |
| S5 | (MH “Outpatients”) |
| S6 | TI (Home care N3 (service or delivery)) OR AB (Home care N3 (service or delivery)) |
| S7 | TI (community health) OR AB (community health) |
| S8 | TI (outpatient or out-patient) OR AB (outpatient or out-patient) |
| S9 | TI (ambulatory care or ambulatory health service or ambulatory health care service or ambulatory primary health service) OR AB (ambulatory care or ambulatory health service or ambulatory health care service or ambulatory primary health service) |
| S10 | (MH “Community Health Services+”) |
| S11 | (MH “community health services” or MH “community health nursing” or MH “community mental health services” or MH “maternal health services” or MH “health services for the aged” or MH “health services, indigenous”) |
| S12 | (MH “Health Services+”) |
| S13 | (MH “Primary Health Care+”) |
| S14 | (MH “Home Care Services+”) |
| S15 | (MH “Ambulatory Care”) |
| S16 | S5 or S6 or S7 or S8 or S9 or S10 or S11 or S12 or S13 or S14 or S15 |
| S17 | (MH “Smartphone”) |
| S18 | (MH “Cell Phone”) |
| S19 | (MH “telecommunications”) |
| S20 | (MH “webcasts+”) |
| S21 | TI ((information and communications technology or information and communications technologies) or ICT or telecommunication or telecommunications) OR AB ((information and communications technology or information and communications technologies) or ICT or telecommunication or telecommunications) |
| S22 | TI (app or software or computer program or computer programs or computer programming) OR AB (app or software or computer program or computer programs or computer programming) |
| S23 | TI (mobile or smartphone or smart phone or cell phone) OR AB (mobile or smartphone or smart phone or cell phone) |
| S24 | TI (tablet or device or iPad or iPhone or android or computer or PC or laptop) OR AB (tablet or device or iPad or iPhone or android or computer or PC or laptop) |
| S25 | TI (telehealth or ehealth or e-health or electronic health or mhealth or m-health or mobile health or m-learning or e-learning or digital health or DHI or virtual care) OR AB (telehealth or ehealth or e-health or electronic health or mhealth or m-health or mobile health or m-learning or e-learning or digital health or DHI or virtual care) |
| S26 | TI (internet-based or web-based) OR AB (internet-based or web-based) |
| S27 | TI (webcast or webinar or podcast or youtube or vimeo or multi-media or multimedia) OR AB (webcast or webinar or podcast or youtube or vimeo or multi-media or multimedia) |
| S28 | TI (video or video-based or video recording) OR AB (video or video-based or video recording) |
| S29 | (MH “Telemedicine+”) |
| S30 | S17 OR S18 OR S19 OR S20 OR S21 OR S22 OR S23 OR S24 OR S25 OR S26 OR S27 OR S28 or S29 |
| S31 | TI (New Zealand or Northland or Auckland or Waikato or Gisborne or Hawkes Bay or Taranaki or Whanganui or Manawatu or Wellington or Marlborough or Nelson or Tasman or West Coast or Canterbury or Christchurch or Dunedin or Otago or Southland) OR AB (New Zealand or Northland or Auckland or Waikato or Gisborne or Hawkes Bay or Taranaki or Whanganui or Manawatu or Wellington or Marlborough or Nelson or Tasman or West Coast or Canterbury or Christchurch or Dunedin or Otago or Southland) |
| S32 | TI (Australia or Tasmania or Victoria or New South Wales or Queensland or Northern Territory or Western Australia or South Australia) OR AB (Australia or Tasmania or Victoria or New South Wales or Queensland or Northern Territory or Western Australia or South Australia) |
| S33 | (MH “Australia+”) |
| S34 | (MH “Austra-Hungary+” or MH “Austria+”) |
| S35 | (MH “Singapore+”) |
| S36 | (MH “Belgium+”) |
| S37 | (MH “Poland+”) |
| S38 | (MH “Croatia+”) |
| S39 | (MH “Czech Republic+”) |
| S40 | (MH “Canada+”) |
| S41 | (MH “Switzerland+”) |
| S42 | (MH “Germany, East” or MH “Germany+” or MH “Germany, West”) |
| S43 | (MH “Spain+”) |
| S44 | (MH “France+”) |
| S45 | (MH “Greece+”) |
| S46 | (MH “United Kingdom+”) |
| S47 | (MH “Scotland+”) |
| S48 | (MH “Northern Ireland+” or MH “Ireland+”) |
| S49 | (MH “Italy+”) |
| S50 | (MH “Japan+”) |
| S51 | (MH “Hong Kong+”) |
| S52 | (MH “Norway+”) |
| S53 | (MH “Netherlands+”) |
| S54 | (MH “Sweden+”) |
| S55 | (MH “Scandinavian and Nordic Countries”) |
| S56 | (MH “New Zealand+”) |
| S57 | (MH “United States+”) |
| S58 | (MH “Alaska”) |
| S59 | S31 OR S32 OR S33 OR S34 OR S35 OR S36 OR S37 OR S38 OR S39 OR S40 OR S41 OR S42 OR S43 OR S44 OR S45 OR S46 OR S47 OR S48 OR S49 OR S50 OR S51 OR S52 OR S53 OR S54 OR S55 OR S56 OR S57 OR S58 |
| S60 | S4 AND S16 AND S30 AND S59 |
| S61 | Limiters: Published Date: 20100101-20201231 |

Database(s): **Embase (Elsevier)**

Search strategy:

| **Search string** | **Query** |
| --- | --- |
| 1 | 'Health Education'/exp |
| 2 | (program* NEAR/2 health):ti,ab,kw |
| 3 | ((patient OR health) NEAR/2 education):ti,ab,kw |
| 4 | #1 OR #2 OR #3 |
| 5 | 'Outpatient'/de |
| 6 | ('Home care' NEAR/3 (service OR delivery)):ti,ab,kw |
| 7 | 'community health':ti,ab,kw |
| 8 | (outpatient* OR 'out-patient*'):ti,ab,kw |
| 9 | ('ambulatory care' OR 'ambulatory health service*' OR 'ambulatory health care service*' OR 'ambulatory primary health service*'):ti,ab,kw |
| 10 | 'Community care'/exp |
| 11 | 'community health nursing'/de OR 'community mental health service'/de OR 'maternal health service'/de OR 'elderly care'/de OR 'indigenous health care'/de |
| 12 | 'Health Service'/exp |
| 13 | 'Primary Health Care'/exp |
| 14 | 'Home Care'/exp |
| 15 | 'Ambulatory Care'/exp |
| 16 | #5 OR #6 OR #7 OR #8 OR #9 OR #10 OR #11 OR #12 OR #13 OR #14 OR #15 |
| 17 | 'mobile phone'/exp |
| 18 | 'telecommunication'/de |
| 19 | 'webcast'/de |
| 20 | 'telehealth'/exp |
| 21 | ((information AND 'communications technolog*') OR ICT* OR telecommunication*):ti,ab,kw |
| 22 | (app OR software OR 'computer program*'):ti,ab,kw |
| 23 | (mobile* OR smartphone OR 'smart phone' OR 'cell* phone'):ti,ab,kw |
| 24 | (tablet OR device OR iPad OR iPhone OR android OR computer OR PC OR laptop):ti,ab,kw |
| 25 | (telehealth OR ehealth OR 'e-health' OR 'electronic health' OR mhealth OR 'm-health' OR 'mobile health' OR 'm-learning' OR 'e-learning' OR 'digital health' OR DHI OR 'virtual care'):ti,ab,kw |
| 26 | ('internet-based' OR 'web-based'):ti,ab,kw |
| 27 | (webcast OR webinar* OR podcast OR youtube OR vimeo OR 'multi-media' OR multimedia):ti,ab,kw |
| 28 | (video OR 'video-based' OR 'video recording'):ti,ab,kw |
| 29 | #17 OR #18 OR #19 OR #20 OR #21 OR #22 OR #23 OR #24 OR #25 OR #26 OR #27 OR #28 |
| 30 | ('New Zealand*' OR Northland OR Auckland OR Waikato OR Gisborne OR 'Hawkes Bay' OR Taranaki OR Whanganui OR Manawatu OR Wellington OR Marlborough OR Nelson OR Tasman OR 'West Coast' OR Canterbury OR Christchurch OR Dunedin OR Otago OR Southland):ti,ab,kw |
| 31 | (Australia* OR Tasmania* OR Victoria* OR 'New South Wales' OR Queensland OR 'Northern Territor*' OR 'Western Australia*' OR 'South Australia*'):ti,ab,kw |
| 32 | 'Australia'/exp |
| 33 | 'Austria-Hungary'/de OR 'Austria'/de |
| 34 | 'Singapore'/de |
| 35 | 'Belgium'/exp |
| 36 | 'Poland'/de |
| 37 | 'Croatia'/de |
| 38 | 'Czech Republic'/de |
| 39 | 'Canada'/exp |
| 40 | 'Switzerland'/de |
| 41 | 'German Democaritc Republic'/de OR 'Germany'/exp OR 'German Federal Republic'/de |
| 42 | 'Spain'/exp |
| 43 | 'France'/exp |
| 44 | 'Greece'/de |
| 45 | 'United Kingdom'/exp |
| 46 | 'Ireland'/de |
| 47 | 'Italy'/exp |
| 48 | 'Japan'/de |
| 49 | 'Hong Kong'/de |
| 50 | 'Norway'/exp |
| 51 | 'Netherlands'/de |
| 52 | 'Sweden'/de |
| 53 | 'Scandinavia'/exp |
| 54 | 'New Zealand'/de |
| 55 | 'United States'/exp |
| 56 | #30 OR #31 OR #32 OR #33 OR #34 OR #35 OR #36 OR #37 OR #38 OR #39 OR #40 OR #41 OR #42 OR #43 OR #44 OR #45 OR #46 OR #47 OR #48 OR #49 OR #50 OR #51 OR #52 OR #53 OR #54 OR #55 |
| 57 | #4 AND #16 AND #29 AND #56 |
| 60 | #57 AND [2010-2020]/py AND [embase]/lim |

Database(s); **Cochrane Central Register of Controlled Trials**

Search Strategy:

| **#** | **Query** |
| --- | --- |
| #1 | MeSH descriptor: [Health Education] in all MeSH products |
| #2 | (program* adj2 health).kf,tw |
| #3 | ((patient or health) adj2 education).kf,tw. |
| #4 | #1 or #2 or #3 |
| #5 | MeSH descriptor: [Outpatients] explode all trees |
| #6 | Community health.kf,tw. |
| #7 | (outpatient* or out-patient*).kf,tw. |
| #8 | (ambulatory care or ambulatory health service* or ambulatory health care service* or ambulatory primary health service*).kf,tw. |
| #9 | MeSH descriptor: [Community Health Services] explode all trees |
| #10 | MeSH descriptor: [Health Services] explode all trees |
| #11 | MeSH descriptor: [Primary Health Care] explode all trees |
| #12 | MeSH descriptor: [Home Care Services] |
| #13 | MeSH descriptor: [Ambulatory Care] explode all trees |
| #14 | #5 #6 or #7 or #8 or #9 or #10 or #11 or #12 or #13 |
| #15 | MeSH descriptor: [Smartphone] explode all trees |
| #16 | MeSH descriptor: [Cell Phone] explode all trees |
| #17 | MeSH descriptor: [Telecommunications] explode all trees |
| #18 | MeSH descriptor: [Webcasts as Topic] explode all trees |
| #19 | MeSH descriptor: [Telemedicine] explode all trees |
| #20 | (app or software or computer program*).kf,tw. |
| #21 | (telehealth or ehealth or e-health or electronic health or mhealth or m-health or mobile health or m-learning or e-learning or digital health or DHI or virtual care).kf,tw. |
| #22 | (tablet or device or iPad or iPhone or android or computer or PC or laptop).kf,tw. |
| #23 | ((information and communications technolog*) or ICT* or telecommunication*).kf,tw. |
| #24 | (internet-based or web-based).kf,tw. |
| #25 | (video or video-based or video recording).kf,tw. |
| #26 | #15 or #16 or #17 or #18 or #19 or #20 or #21 or #22 or #23 or #24 or #25 |
| #27 | #4 and #14 and #26 with publication Year from 2010 to present, in Trials |

**Table S3 - Grey literature sources searched**

|  | |
| --- | --- |
| **Source** | **URL** |
| **Australian sources** | |
| Department of Health, Australian Government | health.gov.au |
| Australian Physiotherapy Association | https://australian.physio/telehealth |
| Victorian Department Of Health and Human Services | https://www.dhhs.vic.gov.au/ |
| Australian Psychological Society | https://www.psychology.org.au |
| Occupational Therapy Australia | https://www.otaus.com.au/member-resources/covid-19/telehealth |
| The Royal Australian College of General Practitioners | www.racgp.org.au |
| Services for Australian Rural and Remote Allied Health (SARRAH) | https://sarrah.org.au |
| Lung Foundation | https://lungfoundation.com.au/ |
| Lung Foundation / Pulmonary Rehabilitation Toolkit | https://pulmonaryrehab.com.au/ |
| HomeBase Pulmonary Rehab | https://homebaserehab.net/ |
| Thoracic Society of Australia and New Zealand | https://www.thoracic.org.au/ |
| Heart Foundation | www.heartfoundation.org.au |
| Heart Online | https://www.heartonline.org.au/ |
| Cardiac Society of Australia and New Zealand | https://www.csanz.edu.au/ |
| NSW Agency for Clinical Innovation | https://www.aci.health.nsw.gov.au/ |
| Queensland Health | https://www.health.qld.gov.au/ |
| SA Health | https://www.sahealth.sa.gov.au/ |
| WA Health | https://ww2.health.wa.gov.au/ |
| Northern Territory Government of Australia Department of Health | https://health.nt.gov.au/ |
| Tasmania Government of Health | https://www.health.tas.gov.au/ |
| ACT Government Health | https://www.health.act.gov.au/ |
| Australian Institute of Health and Welfare | https://www.aihw.gov.au/ |
| Australian Health Professions Australia | https://ahpa.com.au/ |
| Telehealth Victoria | https://telehealthvictoria.org.au/ |
| Digital Health CRC | https://www.digitalhealthcrc.com/ |
| Australian Telehealth Society | https://www.aths.org.au/ |
| **International sources^** | |
| Google scholar | http://scholar.google.com/ |
| Google | http://www.google.com |
| Canadian Physiotherapy Association | https://physiotherapy.ca/ |

^The limited international grey literature search is acknowledged. An extensive search was not feasible within the time constraints of the review and need for rapid dissemination of evidence to Australian health services.

**Table S4. Excluded studies**

| **Reference** | **Reason for exclusion** |
| --- | --- |
| Agboola S et al. Impact of the connected cardiac care program on hospitalisations and mortality: A matched-control analysis. Circulation 2013;128(22):1. | Abstract/conference proceedings only |
| Atreja A et al. Improved quality of care for IBD patients using healthPROMISE App: A randomized, control trial. American Journal of Gastroenterology 2018;113(1):S1. | Abstract/conference proceedings only |
| Ciemins E et al. Using telehealth to provide diabetes care to patients in rural Montana: Findings from the Promoting Realistic Individual Self-Management Program (PRISM). Diabetes Technology and Therapeutics 2013;15(1):S70. | Abstract/conference proceedings only |
| Clark H et al. The effectiveness of web-based patient education and action and coping plans as an adjunct to patient treatment in physiotherapy: A randomized controlled trial. Physiotherapy Theory & Practice 2019;35(10):930-939. | Abstract/conference proceedings only |
| Clark JP and Armstrong D. Telepodiatry in the treatment of lower-extremity ulcers. Journal of Diabetes Science and Technology 2013;7(1):A27. | Abstract/conference proceedings only |
| Cosgrove V et al. An online intervention for bipolar disorder Moodswings 2.0: www.moodswings.net.au." Bipolar Disorders 2013;15(1):97. | Abstract/conference proceedings only |
| Czesak AM et al. Effects of educational text messages as an adjunct to face to face educational programmes for people with diabetes. Diabetic Medicine 2016;33(1):126. | Abstract/conference proceedings only |
| Dahlberg LE et al. Joint academy-an innovative internet-based platform for the management of osteoarthritis. Journal of Orthopaedic Research 2017;35. | Abstract/conference proceedings only |
| Fjell M et al. Patients' experiences of using an interactive app during treatment for breast cancer. Quality of Life Research 2018;27:S20. | Abstract/conference proceedings only |
| Kennedy C et al. Effectiveness of a telemedicine education program for adults with inflammatory arthritis living in rural and remote communities in Ontario. Journal of Rheumatology 2015;42(7):1274-1275. | Abstract/conference proceedings only |
| McLin V et al. KidsETransplant: a platform for liver-transplanted children. Studies in Health Technology & Informatics 2013;192(1):1226. | Abstract/conference proceedings only |
| Nundy S et al. Using text messaging to provide self-management support and care coordination for individuals with chronic disease. Journal of general internal medicine 2013;28:S449-S450. | Abstract/conference proceedings only |
| Penn CL. Telemedicine Expanding in Arkansas Examples--UAMS and Beyond. Journal of the Arkansas Medical Society 2015;112(6):78-80. | Abstract/conference proceedings only |
| Tirimacco R et al. The Country Access to Cardiac Health (CATCH) Program: Evaluation of a telephone phase 2 cardiac rehabilitation program using a standardised program framework and evaluation procedures. Global Heart 2014;9(1):e335-e336. | Abstract/conference proceedings only |
| van Doorn-van Atten MN et al. Effects of a multi-component nutritional telemonitoring intervention on nutritional status, diet quality, physical functioning and quality of life of community-dwelling older adults. British Journal of Nutrition 2018;119(10):1185-1194. | Abstract/conference proceedings only |
| Varnfield M et al. 'Real world' m-health technology supported home-based cardiac rehabilitation - are we there yet? European Heart Journal 2018;39(1):226-227. | Abstract/conference proceedings only |
| Jacobs PG and Saunders GH. New opportunities and challenges for teleaudiology within Department of Veterans Affairs. Journal of Rehabilitation Research & Development 2014;51(5):7. | Editorial |
| Phillips J. Reducing admissions for long-term conditions: is telehealth the answer? British Journal of Community Nursing 2012;17(2):76. | Editorial |
| Portnoy J et al. Telemedicine in the Era of COVID-19. Journal of Allergy and Clinical Immunology: In Practice 2020;8(5):1489-1491. | Editorial |
| Williamson and Owen. An online exercise program plus automated coping skills raining improved pain and function in chronic knee pain. ACP Journal Club 2017;166(12):6-6. | Editorial |
| Markwick L et al. Expanding Telemedicine to Include Primary Care for the Urban Adult. Journal of Health Care for the Poor & Underserved 2015;26(3):771-776. | Healthy sample with no diagnoses of chronic disease |
| Zetterman CV et al. Validation of a virtual preoperative evaluation clinic: a pilot study." Studies in Health Technology & Informatics 2011;163(1):737-739. | Healthy sample with no diagnoses of chronic disease |
| Levinger P et al. A novel web-support intervention to promote recovery following Anterior Cruciate Ligament reconstruction: A pilot randomised controlled trial. Physical Therapy in Sport 2017;27(1):29-37. | Healthy sample with no diagnosis of chronic disease |
| Badger TA et al. Telephone-delivered health education and interpersonal counseling improve quality of life for Latinas with breast cancer and their supportive partners. Psycho-oncology 2013;22(5):1035‐1042. | ICT component is not focus of study |
| Baker DW et al. The effect of progressive, reinforcing telephone education and counseling versus brief educational intervention on knowledge, self-care behaviors and heart failure symptoms. Journal of cardiac failure 2011;17(10):789‐796. | ICT component is not focus of study |
| Bishop D et al. Family Intervention: telephone Tracking (FITT): a pilot stroke outcome study. Topics in stroke rehabilitation 2011;21(supp 1):S63‐74. | ICT component is not focus of study |
| Blumenthal JA et al. The effects of a telehealth coping skills intervention on outcomes in chronic obstructive pulmonary disease: primary results from the INSPIRE-II study. Psychosomatic medicine 2014;76(8):581‐592. | ICT component is not focus of study |
| Bohlin A et al. Childhood obesity treatment: telephone coaching is as good as usual care in maintaining weight loss – a randomized controlled trial. Clinical Obesity 2017;7(4):199-205. | ICT component is not focus of study |
| Carlucci M and Thanavaro J. Early Telemedicine to Promote Continuous Positive Airway Pressure Adherence. Journal for Nurse Practitioners 2019;15(3):e57-e59. | ICT component is not focus of study |
| Chew CL and Yee SL. The rheumatology monitoring clinic in Singapore - A novel advanced practice nurse-/pharmacist-led clinic. Proceedings of Singapore Healthcare 2013;22(1):48-55. | ICT component is not focus of study |
| Egede LE et al. Telephone-Delivered Behavioral Skills Intervention for African American Adults with Type 2 Diabetes: a Randomized Controlled Trial. Journal of General Internal Medicine 2017;32(7):775‐782. | ICT component is not focus of study |
| Konjeti VR et al. A tele-health based technology can effectively triage liver transplant evaluations and minimize futile invasive testing in an integrated health delivery system. Gastroenterology 2017;152(5):S1190. | ICT component is not focus of study |
| Lawler SP et al. Multiple health behavior changes and co-variation in a telephone counseling trial. Annals of Behavioral Medicine 2010;39(3):250‐257. | ICT component is not focus of study |
| Newman L et al. Digital technology use among disadvantaged Australians: implications for equitable consumer participation in digitally-mediated communication and information exchange with health services. Australian Health Review 2012;36(2):125-129. | ICT component is not focus of study |
| O’Neil A et al. Long-term efficacy of a tele-health intervention for acute coronary syndrome patients with depression: 12-month results of the MoodCare randomized controlled trial. European Journal of Preventive Cardiology 2015;22(9):1111-1120. | ICT component is not focus of study |
| O'Brien KM et al. Telephone-based weight loss support for patients with knee osteoarthritis: a pragmatic randomised controlled trial. Osteoarthritis and Cartilage 2018;26(4):485‐494. | ICT component is not focus of study |
| Osofsky HJ et al. Integrated care: meeting mental health needs after the gulf oil spill. Psychiatric Services 2014;65(3):280-283. | ICT component is not focus of study |
| Piras EM and Miele F. Clinical self-tracking and monitoring technologies: negotiations in the ICT-mediated patient–provider relationship. Health Sociology Review 2017;26(1):38-53. | ICT component is not focus of study |
| Rollo ME et al. Cost evaluation of providing evidence-based dietetic services for weight management in adults: In-person versus eHealth delivery. Nutrition & Dietetics 2018;75(1):35-43. | ICT component is not focus of study |
| Schechter CB et al. Costs and effects of a telephonic diabetes self-management support intervention using health educators. Journal of Diabetes and Its Complications 2016;30(2):300‐305. | ICT component is not focus of study |
| Simon P. The role of telemedicine in the organisation of care. Soins: La Revue de Reference Infirmiere2010;750:29-31. | ICT component is not focus of study |
| Smith AC et al. Generating new telehealth services using a whole of community approach: experience in regional Queensland. Journal of Telemedicine & Telecare 2014;20(7):365-369. | ICT component is not focus of study |
| St Clair M and Murtagh D. Barriers to Telehealth Uptake in Rural, Regional, Remote Australia: What Can Be Done to Expand Telehealth Access in Remote Areas?...Health Informatics Conference, August 12-14, 2019, Melbourne, Australia. Studies in Health Technology & Informatics pp. 174-182. | ICT component is not focus of study |
| Ströbl V et al. A combined planning and telephone aftercare intervention for obese patients: effects on physical activity and body weight after one year. Journal of Rehabilitation Medicine 2013;45(2):198‐205. | ICT component is not focus of study |
| Topolovec-Vranic J et al. Traumatic brain injury and mental health: The need for technology-based mental health treatment programs. Brain Injury 2012;26(4-5):562-563. | ICT component is not focus of study |
| Trief PM et al. Health and Psychosocial Outcomes of a Telephonic Couples Behavior Change Intervention in Patients with Poorly Controlled Type 2 Diabetes: a Randomized Clinical Trial. Diabetes Care 2016;39(12):2165‐2173. | ICT component is not focus of study |
| Tucker S et al. A Methodology for Adapting Psychoeducational Content to Mobile Platforms. Studies in Health Technology & Informatics 2015;216:999. | ICT component is not focus of study |
| Vinson MH et al. Design, implementation, and evaluation of population-specific telehealth nursing services. Nursing Economics 2011;29(5):265-272. | ICT component is not focus of study |
| Wachtel TM. Preferred models of cardiac rehabilitation in rural South Australia from a health consumer's perspective. Australian Journal of Advanced Nursing 2011;28(3):30-36. | ICT component is not focus of study |
| Walters J et al. Effects of telephone health mentoring in community-recruited chronic obstructive pulmonary disease on self-management capacity, quality of life and psychological morbidity: A randomised controlled trial. BMJ Open 2013;3(9). | ICT component is not focus of study |
| Woodward-Kron R et al. Educating the patient for health care communication in the age of the world wide web: a qualitative study." Academic Medicine 2014;89(2):318-325. | ICT component is not focus of study |
| Zibrik L et al. Patient and community centered eHealth: Exploring eHealth barriers and facilitators for chronic disease self-management within British Columbia's immigrant Chinese and Punjabi seniors. Health Policy and Technology 2015;4(4):348-356. | ICT component is not focus of study |
| Fitzner and Moss. Telehealth--an effective delivery method for diabetes self-management education? Population Health Management 2013;16(3):169-77 | Included studies do not meet review criteria |
| Allen JK et al. Technology-assisted weight management interventions: systematic review of clinical trials." Telemedicine Journal and E-Health 2014;20(12):1103-1120. | Included studies do not meet review criteria (systematic review) |
| De Greef KP et al. The effects of a pedometer-based behavioral modification program with telephone support on physical activity and sedentary behavior in type 2 diabetes patients. Patient education and counseling 2011;84(2):275‐279. | Included studies do not meet review criteria (systematic review) |
| de Jong JM et al. A Cloud-Based Virtual Outpatient Clinic for Patient-Centered Care: Proof-of-Concept Study. Journal of Medical Internet Research 2018;20(9):64-64. | Included studies do not meet review criteria (systematic review) |
| Fortmann AL et al. 2019. Innovative Diabetes Interventions in the U.S. Hispanic Population. Diabetes Spectrum 2019;32(4):295-301. | Included studies do not meet review criteria (systematic review) |
| Hallensleben C et al. eHealth for people with COPD in the netherlands: A scoping review. International Journal of COPD 2019;14(1):1681-1690. | Included studies do not meet review criteria (systematic review) |
| Tuot DS. and Boulware LE. Telehealth Applications to Enhance CKD Knowledge and Awareness Among Patients and Providers. Advances in Chronic Kidney Disease 2017;24(1):39-45. | Included studies do not meet review criteria (systematic review) |
| Chen I et al. Digitizing Patient Education and Engagement: Implementing an Interactive Patient Care System in a Pediatric Teaching Hospital. CIN: Computers, Informatics, Nursing 2017;35(8):383-384. | Intervention delivered in inpatient context |
| Dilles A et al. Comparison of a computer assisted learning program to standard education tools in hospitalized heart failure patients. European Journal of Cardiovascular Nursing 2011;10(3):187-193. | Intervention delivered in inpatient context |
| Lerret SM et al. Using the Engaging Parents in Education for Discharge (ePED) iPad Application to Improve Parent Discharge Experience. Journal of Pediatric Nursing 2020;52:41-48. | Intervention delivered in inpatient context |
| Pallesen H et al. Patients' and Health Professionals' Experiences of Using Virtual Reality Technology for Upper Limb Training after Stroke: A Qualitative Substudy. Rehabilitation Research and Practice 2018. | Intervention delivered in inpatient context |
| Reilly K et al. A multicomponent transitional care intervention to improve clinical outcomes among patients being discharged after a heart failure admission: A pilot randomized clinical trial. Journal of General Internal Medicine 2016;31(2):S99. | Intervention delivered in inpatient context |
| Schweier R et al. Dissemination strategies and adherence predictors for web-based interventions--how efficient are patient education sessions and email reminders? Health education research 2016;31(3):384-394. | Intervention delivered in inpatient context |
| Zhou Y et al. Effects of a Nurse-Led Phone Follow-up Education Program Based on the Self-efficacy Among Patients with Cardiovascular Disease. Journal of Cardiovascular Nursing 2018;33(1):E15‐E23. | Intervention delivered in inpatient context |
| Shane-McWhorter et al. The Utah Remote Monitoring Project: improving health care one patient at a time. Diabetes Technology & Therapeutics 2014;16(10):653-660. | Intervention not delivered by a health professional |
| Shane-McWhorter et al. Pharmacist-provided diabetes management and education via a telemonitoring program. Journal of the American Pharmacists Association: JAPhA 2015;55(5):516-526. | Intervention not delivered by a health professional |
| Abroms LC et al. A randomized trial of Text2Quit: a text messaging program for smoking cessation. American Journal of Preventive Medicine 2014;47(3):242‐250. | Intervention not delivered by health professional |
| Ackermann RT et al. A randomized comparative effectiveness trial of using cable television to deliver diabetes prevention programming. Obesity 2014;22(7):1601‐1607. | Intervention not delivered by health professional |
| Aguiar EJ et al. Efficacy of the Type 2 Diabetes Prevention Using LifeStyle Education Program RCT. American Journal of Preventive Medicine 2016;50(3): 353-364. | Intervention not delivered by health professional |
| Algeo N et al. A patient and public involvement group study on the usability of the myjointpain.org website. Rheumatology 2015;4:i105. | Intervention not delivered by health professional |
| Alley SJ et al. The effectiveness of a web 2.0 physical activity intervention in older adults - a randomised controlled trial. International Journal of Behavioral Nutrition & Physical Activity 2018;15(1):4. | Intervention not delivered by health professional |
| Al-Ozairi E et al. Diabetes and TelecommunicationS (DATES) study to support self-management for people with type 2 diabetes: a randomized controlled trial. BMC Public Health 2018;18(1): 1249. | Intervention not delivered by health professional |
| Ammerlaan J et al. Feasibility of an online and a in-person version of a self-management program for young adults with a rheumatic disease: experiences of young adults and peer leaders. Pediatric Rheumatology Online Journal 2014;12:10. | Intervention not delivered by health professional |
| Asch DA and Volpp KG. On the Way to Health. LDI Issue Brief 2012;17(9): 1-4. | Intervention not delivered by health professional |
| Ashing K and Rosales M. A telephonic-based trial to reduce depressive symptoms among Latina breast cancer survivors. Psycho-oncology 2014;23(5):507‐515. | Intervention not delivered by health professional |
| Bala N et al. Use of Telehealth to Enhance Care in a Family-Centered Childhood Obesity Intervention. Clinical Pediatrics 2019;58(7):789‐797. | Intervention not delivered by health professional |
| Bantum EO et al. Surviving and thriving with cancer using a Web-based health behavior change intervention: randomized controlled trial. Journal of Medical Internet Research 2014;16(2):e54. | Intervention not delivered by health professional |
| Bashi N et al. Development and testing of an iPad application for teaching self-management to heart failure patients. European Journal of Heart Failure, 2012;11: S1-S2. | Intervention not delivered by health professional |
| Bourbeau J et al. An international randomized study of a home-based self-management program for severe COPD: the COMET. International Journal Of Chronic Obstructive Pulmonary Disease 2016;11:1447‐1451. | Intervention not delivered by health professional |
| Brenes GA et al. A randomized controlled trial of telephone-delivered cognitive-behavioral therapy for late-life anxiety disorders. American Journal Of Geriatric Psychiatry 2012;20(8):707‐716. | Intervention not delivered by health professional |
| Bruno A et al. Home-exercise Childhood Obesity Intervention: A Randomized Clinical Trial Comparing Print Versus Web-based (Move It) Platforms. Journal of Pediatric Nursing 2018;42:e79-e84. | Intervention not delivered by health professional |
| Chan KS. Development of web-based psychoeducation program for caregivers of patients with psychosis. Early Intervention in Psychiatry 2016;10: 67. | Intervention not delivered by health professional |
| Cho Het al. A multi-level usability evaluation of mobile health applications: A case study. Journal of Biomedical Informatics 2018;86:79-89. | Intervention not delivered by health professional |
| Darvall JN et al. Feasibility and acceptability of remotely monitored pedometer-guided physical activity. Anaesthesia & Intensive Care 2016;44(4):501-506. | Intervention not delivered by health professional |
| de Garibay VG et al. Utility of a mHealth App for Self-Management and Education of Cardiac Diseases in Spanish Urban and Rural Areas. Journal of Medical Systems 2016;40(8). | Intervention not delivered by health professional |
| den Bakker CM et al. Electronic Health Program to Empower Patients in Returning to Normal Activities After Colorectal Surgical Procedures: mixed-Methods Process Evaluation Alongside a Randomized Controlled Trial. Journal of Medical Internet Research 2019;21(1):e10674. | Intervention not delivered by health professional |
| Desai PM et al. Impact of telephone reinforcement and negotiated contracts on behavioral predictors of exercise maintenance in older adults with osteoarthritis. American journal of health behavior 2014;38(3):465‐477. | Intervention not delivered by health professional |
| DuBenske LL et al. Web-based cancer communication and decision-making systems: connecting patients, caregivers, and clinicians for improved health outcomes. Medical Decision Making 2010;30(6):732-744. | Intervention not delivered by health professional |
| Dunn C et al. Using synchronous distance-education technology to deliver a weight management intervention. Journal of nutrition education and behavior 2014;46(6):602‐609. | Intervention not delivered by health professional |
| Eakin E et al. Maintenance of physical activity and dietary change following a telephone-delivered intervention. Health psychology 2010;29(6):566‐573. | Intervention not delivered by health professional |
| Gill DP et al. The HealtheSteps TM lifestyle prescription program to improve physical activity and modifiable risk factors for chronic disease: a pragmatic randomized controlled trial. BMC Public Health 2019;19(1):841. | Intervention not delivered by health professional |
| Griffiths PC et al. Tele-Savvy: An Online Program for Dementia Caregivers. American Journal of Alzheimer's Disease & Other Dementias 2018;33(5):269-276. | Intervention not delivered by health professional |
| Helle C et al. Evaluation of an eHealth intervention aiming to promote healthy food habits from infancy -the Norwegian randomized controlled trial Early Food for Future Health. International Journal Of Behavioral Nutrition And Physical Activity 2019;16(1):1. | Intervention not delivered by health professional |
| Levesque DA et al. Randomized trial of a computer-tailored intervention for patients with depression. American Journal Of Health Promotion 2011;26(2):77-89. | Intervention not delivered by health professional |
| Lyden JR et al. Implementing health information technology in a patient-centered manner: patient experiences with an online evidence-based lifestyle intervention. Journal for Healthcare Quality 2013;35(5):47-57. | Intervention not delivered by health professional |
| Maduforo U et al. Diabetes connect: Feasibility of a peer support diabetes intervention for low-income African Americans. Journal of Investigative Medicine 2013;61(2):492. | Intervention not delivered by health professional |
| Mensorio MS et al. Analysis of the efficacy of an internet-based self-administered intervention ("Living Better") to promote healthy habits in a population with obesity and hypertension: An exploratory randomized controlled trial. International Journal Of Medical Informatics 2019;124:13-23. | Intervention not delivered by health professional |
| Noh JH et al. Web-based comprehensive information system for self-management of diabetes mellitus. Diabetes Technology & Therapeutics 2010;12(5): 333‐337. | Intervention not delivered by health professional |
| Nolan RP et al. Randomized Controlled Trial of E-Counseling for Hypertension: REACH. Circulation. Cardiovascular quality and outcomes 2018;11(7): e004420. | Intervention not delivered by health professional |
| Pinto BM et al. A randomized trial to promote physical activity among breast cancer patients. Health psychology 2013;32(6):616‐626. | Intervention not delivered by health professional |
| Quinn CC et al. Cluster-randomized trial of a mobile phone personalized behavioral intervention for blood glucose control. Diabetes care 2011;34(9):1934‐1942. | Intervention not delivered by health professional |
| Rimmer JH et al. Telehealth weight management intervention for adults with physical disabilities: a randomized controlled trial. American Journal Of Physical Medicine & Rehabilitation 2013;92(12):1084‐1094. | Intervention not delivered by health professional |
| Salisbury C et al. Effectiveness of an integrated telehealth service for patients with depression: A pragmatic randomised controlled trial of a complex intervention. The Lancet Psychiatry 2016;3(6):515-525. | Intervention not delivered by health professional |
| Sangster J et al. Effectiveness of a pedometer-based telephone coaching program on weight and physical activity for people referred to a cardiac rehabilitation program: a randomized controlled trial." Journal of cardiopulmonary rehabilitation and prevention 2015;35(2):124‐129. | Intervention not delivered by health professional |
| Spring B et al. Integrating technology into standard weight loss treatment: a randomized controlled trial." JAMA internal medicine 2013;173(2):105‐111. | Intervention not delivered by health professional |
| Toschi, E., et al. (2018). "Use of mobile-based technologies improve diabetes self-management behavior." Diabetes Technology and Therapeutics 20: A81. | Intervention not delivered by health professional |
| Voncken-Brewster V et al. A randomized controlled trial evaluating the effectiveness of a web-based, computer-tailored self-management intervention for people with or at risk for COPD." International Journal of COPD 2015;10:1061-1073. | Intervention not delivered by health professional |
| Walker EA et al. Results of a successful telephonic intervention to improve diabetes control in urban adults: a randomized trial. Diabetes Care 2011;34(1):2‐7. | Intervention not delivered by health professional |
| Whittemore R et al. Type 1 diabetes eHealth psychoeducation: youth recruitment, participation, and satisfaction. Journal of Medical Internet Research 2013;15(1): e15-e15. | Intervention not delivered by health professional |
| Wieland ML et al. Pilot Feasibility Study of a Digital Storytelling Intervention for Immigrant and Refugee Adults With Diabetes. Diabetes Educator 2017;43(4):349-359. | Intervention not delivered by health professional |
| Zheng X et al. Effect of Text Messaging on Risk Factor Management in Patients With Coronary Heart Disease: the CHAT Randomized Clinical Trial. Circulation. Cardiovascular quality and outcomes 2019;12(4): e005616. | Intervention not delivered by health professional |
| Fang R and Deng X. Electronic messaging intervention for management of cardiovascular risk factors in type 2 diabetes mellitus: a randomised controlled trial. J. Clin. Nurs. 2018; 27: 612‐620. | Low or middle income country |
| Hemmati Maslakpak M et al. Effects of Face-to-Face and Telephone-Based Family-Oriented Education on Self-Care Behavior and Patient Outcomes in Type 2 Diabetes: a Randomized Controlled Trial. Journal of Diabetes Research 2017: 8404328. | Low or middle income country |
| Agarwal E et al. E-h ealth support promotes dietary improvements in women previously treated for cancer: results from the Australian women's wellness after cancer program. Supportive Care in Cancer 2018;26(2):S166-S167. | No detail of intervention ICT component(s) |
| Arora PG et al. Dissemination and implementation science in program evaluation: A telemental health clinical consultation case example. Evaluation & Program Planning 2017;60:56-63. | No detail of intervention ICT component(s) |
| Berentsen B et al. Interdisciplinary self-help intervention for IBS-eHealth and patient education." Neurogastroenterology and Motility 2018;30. | No detail of intervention ICT component(s) |
| Berg M et al. Person-centered Web support to women with type 1 diabetes in pregnancy and early motherhood--the development process. Diabetes Technology & Therapeutics 2013;15(1): 20-25. | No detail of intervention ICT component(s) |
| Bishop A et al. PhysioDirect: supporting physiotherapists to deliver telephone assessment and advice services within the context of a randomised trial. Physiotherapy 2013;99(2): 113‐118. | No detail of intervention ICT component(s) |
| Burford S et al. Participatory research design in mobile health: Tablet devices for diabetes self management. Communication & Medicine (Equinox Publishing Group) 2015;12(2/3):145-156. | No detail of intervention ICT component(s) |
| Buysse H et al. Introducing a telemonitoring platform for diabetic patients in primary care: Will it increase the socio-digital divide? Primary Care Diabetes 2013;7(2):119-127. | No detail of intervention ICT component(s) |
| Campbell I et al. Mobile technology as a tool for patient education and self-management in the diabetic population. Diabetes Technology and Therapeutics 2014;16:A109-A110. | No detail of intervention ICT component(s) |
| Campbell, I et al. Mobile technology as a tool for patient education and self-management in the diabetic population. Diabetic Medicine 2014;31:113-114. | No detail of intervention ICT component(s) |
| Chan S et al. A smartphone app for psychoeducation for family caregivers of people living with dementia: A feasibility study. Australian and New Zealand Journal of Psychiatry 2016;50:73. | No detail of intervention ICT component(s) |
| Chorianopoulou A et al. Investigation of the quality and effectiveness of telemedicine in children with diabetes. Studies in Health Technology & Informatics 2015;210:105-109. | No detail of intervention ICT component(s) |
| Chumbler NR et al. A randomized controlled trial on Stroke telerehabilitation: the effects on falls self-efficacy and satisfaction with care. Journal of Telemedicine and Telecare 2015;21(3):139‐143. | No detail of intervention ICT component(s) |
| Clark D et al. Remote Hypertension Management Using Blood Pressure Telemonitoring In A Rural And Low-Income Population. Journal of the American College of Cardiology 2020;75(11): 2060. | No detail of intervention ICT component(s) |
| Clark RA et al. A collaborative approach to developing culturally appropriate heart failure self-care tools for indigenous Australians using multi-media technology. Circulation 2014;130. | No detail of intervention ICT component(s) |
| Cole E. Diabetes care tool puts kids in control. Nursing Standard 2015;29(45):18-20. | No detail of intervention ICT component(s) |
| Dinesen B et al. Interaction between COPD patients and healthcare professionals in a cross-sector tele-rehabilitation programme. Studies In Health Technology And Informatics 2011;169:28‐32. | No detail of intervention ICT component(s) |
| Dowe A et al. COPD hospital admission reduction playbook: Incorporating telehealth in the home. American Journal Of Respiratory And Critical Care Medicine 2019;199(9). | No detail of intervention ICT component(s) |
| Egerton T et al. Development of a service delivery intervention and implementation plan for optimising primary care management of knee osteoarthritis: The partner project. Osteoarthritis and Cartilage 2018;26:S269. | No detail of intervention ICT component(s) |
| Faiman M et al. Diabetes education through shared medical appointment: Utilizing digital health-a comparison of platforms and integrating devices with result review. Diabetes 2019;68. | No detail of intervention ICT component(s) |
| Gonzalez-Morkos B et al. The teen impact experience: a webcast pilot project for teens with cancer and blood diseases. Journal of Pediatric Oncology Nursing 2014;31(5): 272-276. | No detail of intervention ICT component(s) |
| Hoffman L et al. Augmenting mental health in primary care: A 1-Year Study of Deploying Smartphone Apps in a Multi-site Primary Care/Behavioral Health Integration Program. Frontiers in Psychiatry 2019;10(FEB). | No detail of intervention ICT component(s) |
| Jayasena R et al. Monitoring of Chronic Disease in the community: Australian Telehealth Study on Organisational Challenges and Economic Impact. International Journal of Integrated Care (IJIC) 2016;16(6):1-3. | No detail of intervention ICT component(s) |
| Menon A et al. Comparison of clinical characteristics of a diabetes telehealth service with specialist face-to-face outpatients: A cross-sectional survey. Diabetes Technology and Therapeutics 2018;20:A117. | No detail of intervention ICT component(s) |
| Nyberg A et al. Effects of an internet based tool for self-management in patients with COPD-a controlled pragmatic pilot trial. European Respiratory Journal 2017;50. | No detail of intervention ICT component(s) |
| Park ES et al. The initiation of a preoperative and postoperative telemedicine urology clinic. Studies in Health Technology and Informatics 2011;163:425-427. | No detail of intervention ICT component(s) |
| Reguera BJ et al. Efficacy of an integrated internet community program after pulmonary rehabilitation for COPD patients: A pilot randomized control trial. European Respiratory Journal 2017;50. | No detail of intervention ICT component(s) |
| Sabatier R et al. The place of telemedicine in the management of heart failure in community medicine: General practitioner perceptions of a regional telemedicine platform in Normandy, France. European Journal of Heart Failure 2019;21:569-570. | No detail of intervention ICT component(s) |
| Schwamm, LH. Telehealth: seven strategies to successfully implement disruptive technology and transform health care. Health Affairs 2014;33(2): 200-206. | No detail of intervention ICT component(s) |
| Ski C et al. The coaching patients on achieving cardiovascular health (coach) programme: Reducing the treatment gap between Indigenous and non-indigenous Australians. Heart Lung and Circulation 2017;26: S336. | No detail of intervention ICT component(s) |
| Tideman P et al. Country access to cardiac health (CATCH) program: Improving rehospitalisation rates and length of stay for cardiac rehabilitation using innovative telehealth delivery. Heart Lung and Circulation 2015;24:S453. | No detail of intervention ICT component(s) |
| Wallace D et al. Assessing the Need for Mobile Health (mHealth) in Monitoring the Diabetic Lower Extremity. JMIR MHealth and UHealth 2019;7(4): e11879. | No detail of intervention ICT component(s) |
| Weiner E et al. Using the Virtual Reality World of Second Life to Promote Patient Engagement. Studies in Health Technology & Informatics 2016;225:198-202. | No detail of intervention ICT component(s) |
| Zavala M and Millan A. Using telehealth to improve diabetes management in the Hispanic population. Hispanic Health Care International: The Official Journal of The National Association of Hispanic Nurses 2014;12(2): 99-101. | No detail of intervention ICT component(s) |
| Zhang D et al. Attitudes toward telehealth video conferencing among patients with rheumatic disease. Arthritis and Rheumatology 2018;70:2599-2600. | No detail of intervention ICT component(s) |
| Sabesan S. Specialist cancer care through Telehealth models. Australian Journal of Rural Health 2015;23(1):19-23. | No detail of intervention ICT components |
| Abutaleb A et al. Inflammatory Bowel Disease Telemedicine Clinical Trial: impact of Educational Text Messages on Disease-Specific Knowledge Over 1 Year. Inflammatory Bowel Diseases 2018;24(10): 2191‐2197. | Non interactive ICT platform |
| Atack Land Luke R. The impact of validated, online health education resources on patient and community members’ satisfaction and health behaviour. Health Education Journal 2012;71(2):211-218. | Non interactive ICT platform |
| Au LE et al. If You Build It They Will Come: Satisfaction of WIC Participants With Online and Traditional In-Person Nutrition Education. Journal of Nutrition Education & Behavior 2016;48(5): 336-342. | Non interactive ICT platform |
| Baker LC et al. Effects of care management and telehealth: A longitudinal analysis using medicare data. Journal of the American Geriatrics Society 2013;61(9):1560-1567. | Non interactive ICT platform |
| Becker A et al. A new computer-based counselling system for the promotion of physical activity in patients with chronic diseases-Results from a pilot study. Patient Education And Counseling 2011;83(2):195-202. | Non interactive ICT platform |
| Bell AM et al. Mobile phone-based video messages for diabetes self-care support. Journal of Diabetes Science and Technology 2012;6(2):310‐319. | Non interactive ICT platform |
| Bove AA et al. Managing hypertension in urban underserved subjects using telemedicine--a clinical trial. American Heart Journal 2013;165(4): 615‐621. | Non interactive ICT platform |
| Boyde M et al. Pilot testing of a self-care education intervention for patients with heart failure. European Journal of Cardiovascular Nursing 2013;12(1): 39-46. | Non interactive ICT platform |
| Boyde M et al. Self-care educational intervention to reduce hospitalisations in heart failure: A randomised controlled trial. European Journal of Cardiovascular Nursing 2018;17(2):178-185. | Non interactive ICT platform |
| Bradford D et al. Making an APPropriate Care Program for Indigenous Cardiac Disease: Customization of an Existing Cardiac Rehabilitation Program. Studies in Health Technology & Informatics 2015;216: 343-347. | Non interactive ICT platform |
| Byrne, JL et al. A brief eHealth tool delivered in primary care to help parents prevent childhood obesity: a randomized controlled trial. Pediatric Obesity 2018;13(11): 659‐667. | Non interactive ICT platform |
| Carolan-Olah M and Sayakhot P. A randomized controlled trial of a web-based education intervention for women with gestational diabetes mellitus. Midwifery 2019;68:39-47. | Non interactive ICT platform |
| Chau JP et al. An evaluation of a web-based diabetes education program designed to enhance self-management among patients living with diabetes. CIN: Computers, Informatics, Nursing 2012;30(12):672-679. | Non interactive ICT platform |
| Depp CA et al. Augmenting psychoeducation with a mobile intervention for bipolar disorder: a randomized controlled trial. Journal Of Affective Disorders 2015;174: 23‐30. | Non interactive ICT platform |
| Dickinson WP et al. Use of a website to accomplish health behavior change: if you build it, will they come? And will it work if they do? Journal of the American Board of Family Medicine: JABFM 2013;26(2):168-176. | Non interactive ICT platform |
| Dobson R et al. Effectiveness of text message based, diabetes self management support programme (SMS4BG): two arm, parallel randomised controlled trial. BMJ (Clinical research ed.) 2018;361: k1959. | Non interactive ICT platform |
| Douglas N and Free C. Someone batting in my corner': experiences of smoking-cessation support via text message." British Journal Of General Practice 2013;63(616): e768‐776. | Non interactive ICT platform |
| Driesenaar JA et al. Patients' Evaluation of a Preparatory Online Communication Tool for Older Patients With Cancer Preceding Chemotherapy. Cancer Nursing 2020;43(2): E71-E78. | Non interactive ICT platform |
| Gambling T and Long A. Tailoring advice and optimizing response: a case study of a telephone-based support for patients with type 2 diabetes. Family practice 2010;27(2): 179‐185. | Non interactive ICT platform |
| Hekler EB et al. Determining who responds better to a computer- vs. human-delivered physical activity intervention: results from the community health advice by telephone (CHAT) trial. International Journal Of Behavioral Nutrition And Physical Activity 2013;10:109. | Non interactive ICT platform |
| Kerr C et al. The potential of Web-based interventions for heart disease self-management: a mixed methods investigation. Journal of Medical Internet Research 2010;12(4): e56-e56. | Non interactive ICT platform |
| Mameli C et al. Combined use of a wristband and a smartphone to reduce body weight in obese children: randomized controlled trial. Pediatric obesity 2018;13(2): 81‐87. | Non interactive ICT platform |
| Naughton F et al. Randomized controlled trial to assess the short-term effectiveness of tailored web- and text-based facilitation of smoking cessation in primary care (iQuit in practice). Addiction 2014;109(7): 1184‐1193. | Non interactive ICT platform |
| O'Connor PJ et al. Randomized trial of telephone outreach to improve medication adherence and metabolic control in adults with diabetes. Diabetes care 2014;37(12): 3317‐3324. | Non interactive ICT platform |
| Ogrin R et al. Co‐design of an evidence‐based health education diabetes foot app to prevent serious foot complications: a feasibility study. Practical Diabetes 2018;35(6): 203-203. | Non interactive ICT platform |
| Riley AR et al. Dissemination of Evidence-Based Behavioral Advice via Video in Pediatric Primary Care. Clinical Pediatrics 2016;55(2):122-128. | Non interactive ICT platform |
| Rod K. Finding ways to lift barriers to care for chronic pain patients: Outcomes of using internet-based self-management activities to reduce pain and improve quality of life. Pain Research and Management 2016. | Non interactive ICT platform |
| Rondags SMPA et al. HypoAware: development and pilot study of a brief and partly web-based psychoeducational group intervention for adults with Type 1 and insulin-treated Type 2 diabetes and problematic hypoglycaemia. Diabetic Medicine 2016;33(2): 184-191. | Non interactive ICT platform |
| Russell NM et al. Text-Messaging to Support Diabetes Self-Management in a Rural Health Clinic: A Quality Improvement Project. Online Journal of Nursing Informatics 2017;21(2): 9-1. | Non interactive ICT platform |
| Ryhanen AM et al. The effects of Internet or interactive computer-based patient education in the field of breast cancer: a systematic literature review. Patient Education & Counseling 2010;79(1): 5-13. | Non interactive ICT platform |
| Sanderson PR et al. Breast cancer education for Navajo women: a pilot study evaluating a culturally relevant video. Journal of Cancer Education 2010;25(2): 217-223. | Non interactive ICT platform |
| Semere W et al. Caregiving for patients with diabetes in the ERA of online patient portals: Findings from the eclipse study. Journal Of General Internal Medicine 2018;33(2): 135. | Non interactive ICT platform |
| Torkamani M et al. A randomized controlled pilot study to evaluate a technology platform for the assisted living of people with dementia and their carers. Journal of Alzheimer's disease 2014;41(2): 515‐523. | Non interactive ICT platform |
| Widmer RJ et al. Digital health intervention during cardiac rehabilitation: a randomized controlled trial. American Heart Journal 2017;188: 65‐72. | Non interactive ICT platform |
| Xiao H et al. Wellness Coaching for People With Prediabetes: a Randomized Encouragement Trial to Evaluate Outreach Methods at Kaiser Permanente, Northern California, 2013. Preventing chronic disease 2015;12: E207. | Non interactive ICT platform |
| Yu CH et al. A web-based intervention to support self-management of patients with type 2 diabetes mellitus: effect on self-efficacy, self-care and diabetes distress. BMC Medical Informatics & Decision Making 2014;14: 117. | Non interactive ICT platform |
| Yeung D et al. Low-health literacy flashcards & mobile video reinforcement to improve medication adherence in patients on oral diabetes, heart failure, and hypertension medications. Journal American Pharmacy Association. 2017;57(1):30-37. | Non-interactive ICT platform |
| Barker A et al. Evaluation of RESPOND, a patient-centred program to prevent falls in older people presenting to the emergency department with a fall: a randomised controlled trial. PLoS medicine 2019;16(5): e1002807. | Not a chronic disease or risk factor |
| White T et al. Virtual Postoperative Visits for New Ostomates. CIN: Computers, Informatics, Nursing 2019;37(2): 73-79. | Not a chronic disease or risk factor |
| Advocat J and Lindsay J. Internet-based trials and the creation of health consumers. Social Science & Medicine 2010;70(3): 485-492. | Not a health service intervention |
| Alencar MK et al. The efficacy of a telemedicine-based weight loss program with video conference health coaching support. Journal Of Telemedicine And Telecare 2019;25(3): 151‐157. | Not a health service intervention |
| Allam A et al. The effect of social support features and gamification on a web-based intervention for rheumatoid arthritis patients: randomized controlled trial. Journal of Medical Internet Research 2015;17(1): e14-e14. | Not a health service intervention |
| Allen KD et al. Telephone-based self-management of osteoarthritis: a randomized trial. Annals of Internal Medicine 2010;153(9): 570‐579. | Not a health service intervention |
| Alschuler KN et al. Promoting resilience in individuals aging with multiple sclerosis: results from a pilot randomized controlled trial. Rehabilitation Psychology 2018;63(3): 338‐348. | Not a health service intervention |
| Amorim AB et al. Integrating Mobile-health, health coaching, and physical activity to reduce the burden of chronic low back pain trial (IMPACT): a pilot randomised controlled trial. BMC musculoskeletal disorders 2019;20(1): 71. | Not a health service intervention |
| Anderson D et al. Decreasing menopausal symptoms in women undertaking a web-based multi-modal lifestyle intervention: The Women's Wellness Program. Maturitas 2015;81(1): 69-75. | Not a health service intervention |
| Arnold CW et al. BabySTEPS: a sugar tracking electronic portal system for gestational diabetes. Studies in Health Technology & Informatics 2013;192: 1123. | Not a health service intervention |
| Ashton LM et al. Feasibility and preliminary efficacy of the 'HEYMAN' healthy lifestyle program for young men: a pilot randomised controlled trial. Nutrition Journal 2017;16(1): 2. | Not a health service intervention |
| Badger TA et al. Who benefits from a psychosocial counselling versus educational intervention to improve psychological quality of life in prostate cancer survivors? Psychology & Health 2013;28(3): 336‐354. | Not a health service intervention |
| Badger T et al. The effect of psychosocial interventions delivered by telephone and videophone on quality of life in early-stage breast cancer survivors and their supportive partners. Journal Of Telemedicine And Telecare 2013;19(5): 260‐265. | Not a health service intervention |
| Baghaei N et al. Diabetic Mario: Designing and Evaluating Mobile Games for Diabetes Education. Games for Health Journal 2016;5(4): 270-278. | Not a health service intervention |
| Bail J et al. Assessing the feasibility of an online cognitive health education program to address cognitive changes among breast cancer survivors. Cancer Epidemiology Biomarkers and Prevention 2017;26(2). | Not a health service intervention |
| Baker LC et al. Integrated telehealth and care management program for medicare beneficiaries with chronic disease linked to savings. Health Affairs 2011;30(9): 1689-1697. | Not a health service intervention |
| Bannink R et al. Effectiveness of a Web-based tailored intervention (E-health4Uth) and consultation to promote adolescents' health: randomized controlled trial. Journal of Medical Internet Research 2014;16(5): e143. | Not a health service intervention |
| Barnes LE et al. Feasibility study for technology-based cancer education for Latina women from an agricultural community. Cancer Epidemiology Biomarkers and Prevention 2011;20(10). | Not a health service intervention |
| Barrett MA et al. Effect of a mobile health, sensor-driven asthma management platform on asthma control. Annals of Allergy, Asthma and Immunology 2017;119(5): 415-421. | Not a health service intervention |
| Bassilios B et al. Evaluation of an Australian primary care telephone cognitive behavioural therapy pilot. Australian Journal of Primary Health 2014;20(1): 62-73. | Not a health service intervention |
| Befort CA et al. Group versus individual phone-based obesity treatment for rural women. Eating behaviors 2010;11(1): 11‐17. | Not a health service intervention |
| Bollyky JB et al. Remote Lifestyle Coaching Plus a Connected Glucose Meter with Certified Diabetes Educator Support Improves Glucose and Weight Loss for People with Type 2 Diabetes. Journal of Diabetes Research 2018: 3961730. | Not a health service intervention |
| Bradbury K et al. Using mixed methods to develop and evaluate an online weight management intervention. British Journal Of Health Psychology 2015;20(1): 45‐55. | Not a health service intervention |
| Brindal E et al.Design and pilot results of a mobile phone weight-loss application for women starting a meal replacement programme. Journal Of Telemedicine And Telecare 2013;19(3):166‐174. | Not a health service intervention |
| Brown W and Odenthal D. The uses of telemedicine to improve asthma control. Journal of Allergy and Clinical Immunology: In Practice 2015;3(2):300-301. | Not a health service intervention |
| Buis LR et al. Use of a text message program to raise type 2 diabetes risk awareness and promote health behavior change (part I): assessment of participant reach and adoption. Journal of Medical Internet Research 2013;15(12): e281. | Not a health service intervention |
| Buis LR et al. Use of a text message program to raise type 2 diabetes risk awareness and promote health behavior change (part II): assessment of participants' perceptions on efficacy. Journal of Medical Internet Research 2013;15(12): e282. | Not a health service intervention |
| Carlson JA et al. Physical activity and dietary behavior change in Internet-based weight loss interventions: comparing two multiple-behavior change indices. Preventive Medicine 2012;54(1): 50-54. | Not a health service intervention |
| Castro Sweet CM et al. Outcomes of a Digital Health Program With Human Coaching for Diabetes Risk Reduction in a Medicare Population. Journal of Aging & Health 2018;30(5): 692-710. | Not a health service intervention |
| Catenacci VA et al. Changes in physical activity and sedentary behavior in a randomized trial of an internet-based versus workbook-based family intervention study. Journal Of Physical Activity & Health 2014;11(2): 348‐358. | Not a health service intervention |
| Chang MW et al. Mothers In Motion intervention effect on psychosocial health in young, low-income women with overweight or obesity. BMC Public Health 2019;19(1):56. | Not a health service intervention |
| Chen F et al. Clinical and Economic Impact of a Digital, Remotely-Delivered Intensive Behavioral Counseling Program on Medicare Beneficiaries at Risk for Diabetes and Cardiovascular Disease. PLoS ONE 2016;11(10): e0163627. | Not a health service intervention |
| Cherrington AL et al. Diabetes Connect: Developing a Mobile Health Intervention to Link Diabetes Community Health Workers With Primary Care. Journal of Ambulatory Care Management 2015;38(4): 333-345. | Not a health service intervention |
| Cheung KL et al. A Review of the Theoretical Basis, Effects, and Cost Effectiveness of Online Smoking Cessation Interventions in the Netherlands: A Mixed-Methods Approach. Journal of Medical Internet Research 2017;19(6): e230. | Not a health service intervention |
| Chiswell M et al. Using Webinars for the Education of Health Professionals and People Affected by Cancer: Processes and Evaluation. Journal of Cancer Education 2018;33(3):583-591. | Not a health service intervention |
| Christensen H et al. Prevention of generalized anxiety disorder using a web intervention, iChill: randomized controlled trial. Journal of Medical Internet Research 2014;16(9): e199. | Not a health service intervention |
| Colkesen EB et al. Effects on cardiovascular disease risk of a web-based health risk assessment with tailored health advice: a follow-up study. Vascular Health & Risk Management 2011;7:67-74. | Not a health service intervention |
| Concotelli-Fisk N and Larroque C. Cyber CF a telehealth adolescent support group. Pediatric Pulmonology 2012;47:388-389. | Not a health service intervention |
| Dahlberg LE et al. An innovative internet-based platform for the management of osteoarthritis. Osteoarthritis and Cartilage 2017;25:S339-S340. | Not a health service intervention |
| Davis AM et al. Treating rural pediatric obesity through telemedicine: outcomes from a small randomized controlled trial. Journal of pediatric psychology 2013;38(9): 932‐943. | Not a health service intervention |
| Delisle Nyström C et al. A 12-month follow-up of a mobile-based (mHealth) obesity prevention intervention in pre-school children: the MINISTOP randomized controlled trial. BMC Public Health 2018;18(1): 658. | Not a health service intervention |
| Devan H et al. Current practices of health care providers in recommending online resources for chronic pain self-management. Journal of Pain Research 2019;12: 2457-2472. | Not a health service intervention |
| Devries KM et al. Preventing smoking relapse using text messages: analysis of data from the txt2stop trial. Nicotine & tobacco research 2013;15(1): 77‐82. | Not a health service intervention |
| Donnelly JE et al. Equivalent weight loss for weight management programs delivered by phone and clinic. Obesity 2013;21(10): 1951‐1959. | Not a health service intervention |
| Doorenbos AZ et al. Developing the Native People for Cancer Control Telehealth Network. Telemedicine Journal & E-Health 2011;17(1): 30-4. | Not a health service intervention |
| DuBenske LL et al. CHESS improves cancer caregivers' burden and mood: results of an eHealth RCT. Health psychology 2014;33(10): 1261‐1272. | Not a health service intervention |
| Duncan M et al. Effectiveness of a web- and mobile phone-based intervention to promote physical activity and healthy eating in middle-aged males: randomized controlled trial of the ManUp study. Journal of Medical Internet Research 2014;16(6): e136. | Not a health service intervention |
| Eakin EG et al. Six-month outcomes from living well with diabetes: a randomized trial of a telephone-delivered weight loss and physical activity intervention to improve glycemic control. Annals of Behavioral Medicine 2013;46(2):193‐203. | Not a health service intervention |
| Ebert DD et al. The 6-month effectiveness of Internet-based guided self-help for depression in adults with Type 1 and 2 diabetes mellitus. Diabetic Medicine 2017;34(1):99-107. | Not a health service intervention |
| Ehde et al. Efficacy of a Telephone-Delivered Self-Management Intervention for Persons With Multiple Sclerosis: a Randomized Controlled Trial With a One-Year Follow-Up. Archives Of Physical Medicine And Rehabilitation 2015;96(11):1945‐58. | Not a health service intervention |
| Elfeddali I et al. Preventing smoking relapse via Web-based computer-tailored feedback: a randomized controlled trial. Journal of Medical Internet Research 2012;14(4): e109. | Not a health service intervention |
| Fjeldsoe BS et al. Get Healthy, Stay Healthy: evaluation of the Maintenance of Lifestyle Changes Six Months After an Extended Contact Intervention. JMIR MHealth and UHealth 2019;7(3):e11070. | Not a health service intervention |
| Friederichs SA et al. Motivational interviewing in a web-based physical activity intervention: questions and reflections. Health Promotion International 2015;30(3): 803-815. | Not a health service intervention |
| Fukuoka Y et al. Innovation to motivation--pilot study of a mobile phone intervention to increase physical activity among sedentary women. Preventive Medicine 2010;51(3-4): 287-289. | Not a health service intervention |
| Gerber BS et al. Video telehealth for weight maintenance of African-American women. Journal Of Telemedicine And Telecare 2013;19(5): 266‐272. | Not a health service intervention |
| Godino JG et al. Text messaging and brief phone calls for weight loss in overweight and obese English- and Spanish-speaking adults: a 1-year, parallel-group, randomized controlled trial. PLoS medicine 2019;16(9): e1002917. | Not a health service intervention |
| Gomez Quiñonez S et al. mHealth or eHealth? Efficacy, Use, and Appreciation of a Web-Based Computer-Tailored Physical Activity Intervention for Dutch Adults: a Randomized Controlled Trial. Journal of Medical Internet Research 2016;18(11): e278. | Not a health service intervention |
| Goode AD et al. A telephone-delivered physical activity and dietary intervention for type 2 diabetes and hypertension: does intervention dose influence outcomes? American Journal Of Health Promotion 2011;25(4): 257‐263. | Not a health service intervention |
| Griauzde D et al. A Mobile Phone-Based Program to Promote Healthy Behaviors Among Adults With Prediabetes Who Declined Participation in Free Diabetes Prevention Programs: mixed-Methods Pilot Randomized Controlled Trial. JMIR MHealth and UHealth 2019;7(1): e11267. | Not a health service intervention |
| Guilcher SJ et al. Spanning boundaries into remote communities: an exploration of experiences with telehealth chronic disease self-management programs in rural northern ontario, Canada. Telemedicine Journal & E-Health 2013;19(12): 904-909. | Not a health service intervention |
| Hart T et al. Analyzing the ingredients of a telephone counseling intervention for traumatic brain injury. Disability and rehabilitation 2013;35(19):1668‐1675. | Not a health service intervention |
| Härter M et al. Effectiveness of Telephone-Based Health Coaching for Patients with Chronic Conditions: a Randomised Controlled Trial. PloS one 2016;11(9): e0161269. | Not a health service intervention |
| Heapy AA et al. Interactive Voice Response-Based Self-management for Chronic Back Pain: the COPES Noninferiority Randomized Trial. JAMA internal medicine 2017;177(6): 765‐773. | Not a health service intervention |
| Helle C et al. Early food for future health: a randomized controlled trial evaluating the effect of an eHealth intervention aiming to promote healthy food habits from early childhood. BMC Public Health 2017;17(1): 729. | Not a health service intervention |
| Hitoshi F et al. A Randomized Controlled Trial to Evaluate the Effects of Health Guidance with Video Call as Compared to Face-to-Face Health Guidance. International Medical Journal 2017;24(2): 186-191. | Not a health service intervention |
| Jarvela-Reijonen E et al. The effects of acceptance and commitment therapy on eating behavior and diet delivered through face-to-face contact and a mobile app: a randomized controlled trial. International Journal of Behavioral Nutrition & Physical Activity 2018;15(1): 22. | Not a health service intervention |
| Johnson KE et al. Telemedicine-Based Health Coaching Is Effective for Inducing Weight Loss and Improving Metabolic Markers. Telemedicine journal and e-health 2019;25(2): 85‐92. | Not a health service intervention |
| Kao DP et al. Impact of a Telehealth and Care Management Program on All-Cause Mortality and Healthcare Utilization in Patients with Heart Failure. Telemedicine Journal & E-Health 2016;22(1): 2-11. | Not a health service intervention |
| Karhula T et al. Telemonitoring and Mobile Phone-Based Health Coaching Among Finnish Diabetic and Heart Disease Patients: randomized Controlled Trial. Journal of Medical Internet Research 2015;17(6): e153. | Not a health service intervention |
| Kasper J et al. A new graphical format to communicate treatment effects to patients-A web-based randomized controlled trial. Health Expectations 2017;20(4): 797-804. | Not a health service intervention |
| Knight E et al. Health promotion through primary care: enhancing self-management with activity prescription and mHealth. Physician and Sports Medicine 2014;42(3): 90‐99. | Not a health service intervention |
| Kornman KP et al. Electronic therapeutic contact for adolescent weight management: the Loozit study. Telemedicine Journal And E-Health 2010;16(6): 678‐685. | Not a health service intervention |
| Kvedar J et al. Connected health: a review of technologies and strategies to improve patient care with telemedicine and telehealth. Health Affairs 2014;33(2): 194-199. | Not a health service intervention |
| Leow MQH. and Chan SWC. Methods of delivering psychoeducation intervention for caregivers of a person with advanced cancer. Annals of the Academy of Medicine Singapore 2015;44(10): S234. | Not a health service intervention |
| Lim S et al. Comparing a telephone- and a group-delivered diabetes prevention program: characteristics of engaged and non-engaged postpartum mothers with a history of gestational diabetes. Diabetes Research and Clinical Practice 2017;126: 254‐262. | Not a health service intervention |
| Nguyen B et al. Two-year outcomes of an adjunctive telephone coaching and electronic contact intervention for adolescent weight-loss maintenance: the Loozit randomized controlled trial. International Journal Of Obesity 2013;37(3): 468‐472. | Not a health service intervention |
| Nobis S et al. Efficacy of a web-based intervention with mobile phone support in treating depressive symptoms in adults with type 1 and type 2 diabetes: a randomized controlled trial. Diabetes Care 2015;38(5): 776‐783. | Not a health service intervention |
| Nuovo J. The Impact of a Diabetes Self-Management Education Program Provided Through a Telemedicine Link to Rural California Health Care Clinics. Health Services Insights 2013;(6): 1-7. | Not a health service intervention |
| Nyenwe EA et al. Improving diabetes care via telemedicine: Lessons from the addressing diabetes in Tennessee (ADT) project. Diabetes Care 2011;34(3): e34. | Not a health service intervention |
| Odnoletkova I et al. Optimizing diabetes control in people with Type 2 diabetes through nurse-led telecoaching. Diabetic medicine 2016;33(6):777‐785. | Not a health service intervention |
| Odnoletkova I et al. "Patient and provider acceptance of telecoaching in type 2 diabetes: a mixed-method study embedded in a randomised clinical trial. BMC Medical Informatics & Decision Making 2016;16: 142-142. | Not a health service intervention |
| Odnotletkova I et al. Delivering Diabetes Education through Nurse-Led Telecoaching. Cost-Effectiveness Analysis. PloS one 2016;11(10):e0163997. | Not a health service intervention |
| Oksman E et al. Cost-effectiveness analysis for a tele-based health coaching program for chronic disease in primary care. BMC health services research 2017;17(1): 138. | Not a health service intervention |
| O'Neil PM et al. Randomized controlled trial of a nationally available weight control program tailored for adults with type 2 diabetes. Obesity 2016;24(11): 2269‐2277. | Not a health service intervention |
| Onrust S et al. Use of social network facebook for mental health prevention and counselling. European Psychiatry 2015;30: 222. | Not a health service intervention |
| Osborne R et al. 'Stepping up when arthritis and pain get you down': The new flexible web-based system to deliver evidencebased patient education and self-management support. Internal Medicine Journal 2012;42: 31. | Not a health service intervention |
| Partridge SR et al. Improved eating behaviours mediate weight gain prevention of young adults: moderation and mediation results of a randomised controlled trial of TXT2BFiT, mHealth program. International journal of behavioral nutrition and physical activity 2016;13: 44. | Not a health service intervention |
| Peimani M et al. Effectiveness of short message service-based intervention (SMS) on self-care in type 2 diabetes: a feasibility study. Primary Care Diabetes 2016;10(4): 251‐258. | Not a health service intervention |
| Petitte TM et al. Feasibility Study: Home Telemonitoring for Patients With Lung Cancer in a Mountainous Rural Area. Oncology Nursing Forum 2014;41(2): 153-161. | Not a health service intervention |
| Pignatiello A et al. Child and youth telepsychiatry in rural and remote primary care. Child & Adolescent Psychiatric Clinics of North America 2011;20(1): 13-28. | Not a health service intervention |
| Price S et al. Development and Implementation of an Interactive Text Messaging Campaign to Support Behavior Change in a Childhood Obesity Randomized Controlled Trial. Journal Of Health Communication 2015;20(7): 843‐850. | Not a health service intervention |
| Pugh NE et al. Internet therapy for postpartum depression: a case illustration of emailed therapeutic assistance. Archives of Women's Mental Health 2014;17(4): 327-337. | Not a health service intervention |
| Quinn CC et al. Mobile Diabetes Intervention for Glycemic Control in 45- to 64-Year-Old Persons With Type 2 Diabetes. Journal of applied gerontology 2016;35(2): 227‐243. | Not a health service intervention |
| Rosen D et al. Increasing self-knowledge: Utilizing tele-coaching for patients with congestive heart failure. Social Work in Health Care 2016;55(9): 711-719. | Not a health service intervention |
| Ross KM and Wing RR. Impact of newer self-monitoring technology and brief phone-based intervention on weight loss: a randomized pilot study. Obesity 2016;24(8): 1653‐1659. | Not a health service intervention |
| Royer HR et al. Formative Research for the Development of an Interactive Web-Based Sexually Transmitted Disease Management Intervention for Young Women. CIN: Computers, Informatics, Nursing 2013;31(9): 430-438. | Not a health service intervention |
| Sakane N et al. Effects of telephone-delivered lifestyle support on the development of diabetes in participants at high risk of type 2 diabetes: J-DOIT1, a pragmatic cluster randomised trial. BMJ Open 2015;5(8): e007316. | Not a health service intervention |
| Sherifali D et al. The Diabetes Health Coaching Randomized Controlled Trial: rationale, Design and Baseline Characteristics of Adults Living With Type 2 Diabetes. Canadian Journal Of Diabetes 2019;43(7): 477‐482. | Not a health service intervention |
| Ski CF et al. Improving access and equity in reducing cardiovascular risk: Coaching patients On Achieving Cardiovascular Health (COACH). European Journal of Cardiovascular Nursing 2015;14: 1-2. | Not a health service intervention |
| Skobel E et al. Internet-based training of coronary artery patients: the Heart Cycle Trial. Heart and Vessels 2017;32(4): 408‐418. | Not a health service intervention |
| Sohn S. et al. Costs and Benefits of Personalized Healthcare for Patients with Chronic Heart Failure in the Care and Education Program “Telemedicine for the Heart”. Telemedicine and e-Health. 2012;18(3):198-204. | Not a health service intervention |
| Spring B et al. Effects of an abbreviated obesity intervention supported by mobile technology: the ENGAGED randomized clinical trial. Obesity 2017;25(7): 1191‐1198. | Not a health service intervention |
| Stacey D et al. Patient and family experiences with accessing telephone cancer treatment symptom support: a descriptive study. Supportive Care in Cancer 2016;24(2): 893-901. | Not a health service intervention |
| Storm V et al. Effectiveness of a Web-Based Computer-Tailored Multiple-Lifestyle Intervention for People Interested in Reducing their Cardiovascular Risk: A Randomized Controlled Trial. Journal of Medical Internet Research 2016;18(4): e78. | Not a health service intervention |
| Tabaei BP et al. Impact of a telephonic intervention to improve diabetes control on health care utilization and cost for adults in South Bronx, New York. Diabetes care 2020;43(4): 743-750. | Not a health service intervention |
| Tan G et al. Improving access to care for women veterans suffering from chronic pain and depression associated with trauma. Pain Medicine 2013;4(7): 1010-1020. | Not a health service intervention |
| Turner-McGrievy GM and Tate DF. Are we sure that Mobile Health is really mobile? An examination of mobile device use during two remotely-delivered weight loss interventions. International Journal Of Medical Informatics 2014;83(5): 313‐319. | Not a health service intervention |
| Vadheim LM et al. Telehealth delivery of the diabetes prevention program to rural communities. Translational Behavioral Medicine 2017;7(2): 286-291. | Not a health service intervention |
| van Doorn-van Atten MN et al. Implementation of a multicomponent telemonitoring intervention to improve nutritional status of community-dwelling older adults: a process evaluation. Public Health Nutrition 2019;22(2):363-374. | Not a health service intervention |
| Varnfield M et al. Uptake of a technology-assisted home-care cardiac rehabilitation program. Medical Journal of Australia 2011;194(4): S15-S19. | Not a health service intervention |
| Volpe T et al. Mental health services for Nunavut children and youth: evaluating a telepsychiatry pilot project. Rural & Remote Health 2014;14(2): 2673. | Not a health service intervention |
| Warner MM et al. Patients' Experiences and Perspectives of Telehealth Coaching with a Dietitian to Improve Diet Quality in Chronic Kidney Disease: A Qualitative Interview Study. Journal of the Academy of Nutrition & Dietetics 2019;119(8): 1362-1374. | Not a health service intervention |
| Wiecha JM et al. Evaluation of a web-based asthma self-management system: A randomised controlled pilot trial. BMC Pulmonary Medicine 2015;15(1). | Not a health service intervention |
| Wilhelm S et al. Cognitive-Behavioral Therapy in the Digital Age: Presidential Address. Behavior Therapy 2020;51(1): 1-14. | Not a health service intervention |
| Wingo et al. Lessons learned from a blended telephone/e-health platform for caregivers in promoting physical activity and nutrition in children with a mobility disability. Disability And Health Journal 2020;13(1):100826 | Not a health service intervention |
| Yardley L et al. Evaluation of a Web-based intervention providing tailored advice for self-management of minor respiratory symptoms: exploratory randomized controlled trial. Journal of Medical Internet Research 2010;12(4): e66. | Not a health service intervention |
| Ko JM and Lee JK. Effects of a coaching program on comprehensive lifestyle modification for women with gestational diabetes mellitus. Journal of Korean Academy of Nursing 2014;44(6): 672‐681. | Not published in English |
| Laux G. Online-/Internet-based psychological therapies for depression - a summarizing report. Journal fur Neurologie, Neurochirurgie und Psychiatrie 2017;18(1): 16-24. | Not published in English |
| Lee J and Lee H. The Effects of Smart Program for Patients Who Underwent Percutaneous Coronary Intervention (SP-PCI) on Disease-Related Knowledge, Health Behavior, and Quality of Life: a Non-Randomized Controlled Trial. Journal of Korean Academy of Nursing 2017;47(6):756‐769. | Not published in English |
| Theissing J et al. Liveonline aftercare in patients with abdominal obesity in cardio-diabetological rehabilitation: findings of a randomized controlled study. Die rehabilitation 2013;52(3): 153‐154. | Not published in English |
| Bannink R et al. Evaluation of computer-tailored health education ('E-health4Uth') combined with personal counselling ('E-health4Uth + counselling') on adolescents' behaviours and mental health status: design of a three-armed cluster randomised controlled trial. BMC Public Health 2012;12(1): 1083-1083. | Study protocol |
| Blackler K et al. Improving access to psychological services for people with cancer: A randomised controlled trial of an interactive web-based intervention. Asia-Pacific Journal of Clinical Oncology 2014;10: 92. | Study protocol |
| Boyde M et al. The Self-care Educational Intervention for Patients With Heart Failure. Journal of Cardiovascular Nursing 2017;32(2): 165-170. | Study protocol |
| Broekhuizen K et al. A tailored lifestyle intervention to reduce the cardiovascular disease risk of individuals with Familial Hypercholesterolemia (FH): design of the PRO-FIT randomised controlled trial. BMC Public Health 2010;10: 69. | Study protocol |
| Carlson J et al. Effectiveness of Telebehavioral Health Program Nurse Case Managers (NCM): Data Collection Tools and the Process for NCM-Sensitive Outcome Measures. US Army Medical Department Journal 2014;36-45. | Study protocol |
| Castro A et al. Efficacy of low-intensity psychological intervention applied by ICTs for the treatment of depression in primary care: A controlled trial. BMC Psychiatry 2015;15(1). | Study protocol |
| Chambers SK et al. A randomised controlled trial of a mindfulness intervention for men with advanced prostate cancer. BMC Cancer 2013;13. | Study protocol |
| Eakin EG et al. Living Well with Diabetes: a randomized controlled trial of a telephone-delivered intervention for maintenance of weight loss, physical activity and glycaemic control in adults with type 2 diabetes. BMC Public Health 2010;10: 452. | Study protocol |
| Egede LE et al. Rationale and design: telephone-delivered behavioral skills interventions for Blacks with Type 2 diabetes. Trials 2010;11: 35. | Study protocol |
| Farmer A et al. Self-management support using an Internet-linked tablet computer (the EDGE platform)-based intervention in chronic obstructive pulmonary disease: protocol for the EDGE-COPD randomised controlled trial. BMJ Open 2014;4(1): e004437. | Study protocol |
| Ferrara A et al. A pragmatic cluster randomized clinical trial of diabetes prevention strategies for women with gestational diabetes: design and rationale of the Gestational Diabetes' Effects on Moms (GEM) study. BMC pregnancy and childbirth 2014;14: 21. | Study protocol |
| Kraal JJ et al. Effects and costs of home-based training with telemonitoring guidance in low to moderate risk patients entering cardiac rehabilitation: the FIT@Home study. BMC cardiovascular disorders 2013;13: 82. | Study protocol |
| Lynch CP et al. Tablet-Aided BehavioraL intervention EffecT on Self-management skills (TABLETS) for Diabetes. Trials 2016;17: 157. | Study protocol |
| Napolitano MA et al. Using social media to deliver weight loss programming to young adults: design and rationale for the Healthy Body Healthy U (HBHU) trial. Contemporary clinical trials 2017;60:1‐13. | Study protocol |
| Perri MG et al. Design of the Rural LEAP randomized trial: an evaluation of extended-care programs for weight management delivered via group or individual telephone counseling. Contemporary clinical trials 2019;76: 55‐63. | Study protocol |
| Plotnikoff RC et al. Alberta Diabetes and Physical Activity Trial (ADAPT): a randomized theory-based efficacy trial for adults with type 2 diabetes--rationale, design, recruitment, evaluation, and dissemination. Trials 2010;11: 4. | Study protocol |
| Sevick MA et al. The Healthy Hearts and Kidneys (HHK) study: design of a 2×2 RCT of technology-supported self-monitoring and social cognitive theory-based counseling to engage overweight people with diabetes and chronic kidney disease in multiple lifestyle changes. Contemporary Clinical Trials 2018;64:265‐273. | Study protocol |
| Shah BR et al. Secondary prevention risk interventions via telemedicine and tailored patient education (SPRITE): a randomized trial to improve postmyocardial infarction management. Circulation. Cardiovascular quality and outcomes 2011;4(2): 235‐242. | Study protocol |
| van Vugt M et al. Web-based self-management with and without coaching for type 2 diabetes patients in primary care: design of a randomized controlled trial. BMC Endocrine Disorders 2013;13(1): 53-59. | Study protocol |
| Williams NJ et al. Telephone-delivered behavioral intervention among blacks with sleep apnea and metabolic syndrome: study protocol for a randomized controlled trial. Trials 2014;15: 225. | Study protocol |

**Table S5. Included program characteristics**

| **Name of health program (country, years implemented)** | **Program description (community health setting)** | **ICT platform (Health professional delivering program)** | **Target cohort** |
| --- | --- | --- | --- |
| Cancer | | | |
| Telephone interpersonal counselling (TIPC) and Supportive Health Education (SHE)[46] (United States, 2013-2017) | The counselling intervention (TIPC) was delivered over the telephone and addressed mood and affect management, emotional expression, communication skills, social support, cancer information, resources and referral. This was compared to SHE which involved sending resources to participants and a follow up phone call. (Community cancer center) | Telephone (Social Work) | Latina patients and carers/families |
| Telephone-based symptom management (TSM)[47] (United States, 2013-2015) | A telephone-based symptom self-management (TSM) intervention was implemented for lung cancer patients and caregivers jointly participating and compared to education. Intervention contained evidence-based cognitive behavioral and emotion-focused therapy. (Community cancer center) | Telephone (Social Work) | Patients and carers |
| Education or Motivational-Interviewing-Based Coaching[48] (United States, not reported) | Those in the education group viewed a video on managing pain, whereas the coaching group received the same intervention in addition to four 30min telephone sessions utilizing motivational interviewing techniques. These two groups were compared to usual care (control). (Community cancer center) | Telephone (Nursing) | Patients |
| Cardiovascular disease | | | |
| Virtual cardiac rehabilitation program (vCRP)[49,50] (Canada, 2008-2012) | A 12-week virtual cardiac rehabilitation program was implemented for patients following an acute cardiac event. This included online forms, one-on-one chat sessions with a nurse case manager, exercise specialist and dietician involvement, weekly education sessions in slide presentation form and group chat sessions. (Outpatient hospital) | Internet-based (Multi-disciplinary) | Patients |
| CHANGE Intervention[51] (United States, 2009-2010) | Involved a 12-month intervention with two components: self-management education via monthly telephone calls, and medication management facilitation. (Outpatient hospital) | Telephone (Nursing) | Patients |
| Collaborative Care program for depression following a coronary artery bypass graft (CABG)[52] (United States, 2004-2008) | Intervention included a mail out workbook to enhance self-care for depression for patients who had a CABG and contact with a nurse care manager via telephone. The nurse care manager then liaised with the psychiatrist weekly to review participants and called participants bi-weekly to assess depression status and monitor pharmacotherapy. (Outpatient hospital) | Telephone (Nursing) | Patients |
| Illawarra and  Shoalhaven Healthy Heart Project[53] (Australia, 2005-2006) | Intervention group received a pedometer and were instructed to record steps per day for 6 weeks during which they received telephone behavioral counselling and goal setting sessions. They then received "booster" phone calls at 12 and 18 weeks to offer feedback and support. (Outpatient hospital) | Telephone (Multi-disciplinary) | Patients |
| Synchronous telehealth program for chronic CVD[54] (Taiwan, 2009-2013) | Intervention involved a synchronous telehealth program with real-time transmission of patient data (electrocardiography, BP, HR, oximetry, blood glucose levels as appropriate) to the telehealth center. The nurse case manager received and reviewed data, and took action as required. Daily telephone interviews between nurses and participants and decision support was also provided. (Outpatient hospital) | Telehealth (Nurse case manager) | Patients |
| Home-based telerehabilitation [55,56] (Australia, 2013-2016) | Involved a 12-week telerehabilitation program delivered via synchronous videoconferencing platform to groups of up to four participants. Two-way communication enabled all participants and physiotherapist to interact. Education presentation delivered electronically as recorded from centre-based program. (Outpatient hospital) | Videoconferencing (Physiotherapy) | Patients |
| The Teledialog Project[57](Denmark, 2012-2014) | A three-month program where patients recently discharged from hospital (post-cardiac event) were given a tablet and were required to monitor their blood pressure, pulse, weight and physical activity (steps). Data was transmitted to health professionals who monitored patients and contacted them if there were any abnormal findings. (Outpatient hospital) | Telemonitoring and internet-based (Multi-disciplinary) | Patients |
| Telemonitored exercise-based Cardiac Rehabilitation (TCR)[58] (Denmark, 2014-2015) | A 12-week intervention contained multiple components including a combination of face to face group based exercise training and telemonitoring which involved email, skype, text-messaging and phone calls between physiotherapists and participants. (Outpatient hospital) | Internet-based (Physiotherapy) | Patients |
| Nurse-Led Collaborative Management using Telemonitoring[59] (Japan, 2013-2015) | A 12-month three-arm RCT where participants receiving usual care were given one education session (standard heart failure education). This was compared to the intervention Self-Management (SM) group who were given monthly 30-minute education sessions for the first six months and the Collaborative Management (CM) group who were given the same six education sessions in addition to nurse-led telemonitoring of daily weight, BP and pulse rate. (Outpatient hospital) | Telemonitoring and internet-based (Nursing) | Patients and carers/families |
| Community Outreach Heart Health and Risk Reduction Trial (COHRT)[60] (Canada, not reported) | Intervention included telehealth counselling to small groups compared to brief counselling (control). (Outpatient hospital) | Telephone (Multi-disciplinary) | Patients |
| ProActive Heart trial[61,62] (Australia, 2007-2010) | The 'ProActive Heart program' focused on key determinants of health behavior, knowledge of the risks and benefits of the behavior, building participant confidence to engage in behaviors under different circumstances, outcome expectations, and targeted strategies for achieving positive health behavior change. Participants received up to ten 30-minute scripted telephone coaching sessions which was compared to usual care. (Outpatient hospital) | Telephone (Nursing) | Patients |
| Tele-nutrition intervention for men at risk of CVD[63] (United States, 2016) | The intervention involved a 12-week tele-nutrition intervention which was compared to usual care (control) for men at risk of cardiovascular disease. (Primary care practices) | Video conferencing (Dietetics) | Patients |
| Other chronic diseases | | | |
| Acquired Brain Injury  Move it to improve it program[64] (Australia, 2013) | Intervention was a 20-week multimodal therapy program (Mitii) incorporating interactive and game elements targeting gross motor, and cognitive and visual perception. (Home-based) | Internet-based (Multi-disciplinary) | Patients and carers |
| Multiple chronic diseases  Telehealth program[65](United Kingdom, 2008-2010) | Variations of telehealth were delivered to participants in primary care practices, and focused on monitoring vital signs, symptoms and self-management behaviors. Health education and review was provided by specialist nurses. (Primary care practices) | Telehealth (Nursing) | Patients |
| Parkinson disease  mHealth-mediated exercise program[66] (United States, 2013-2015) | An mHealth-mediated exercise program intervention using a pedometer and supported by a mobile health application (monitored by a physical therapist) was compared to an exercise program without technology. (Outpatient hospital) | Internet-based (Physiotherapy) | Patients |
| Asthma  Telephone Asthma Program (TAP)[67,68](United States, 2004-2005) | The Telephone Asthma Program (TAP) was a 12-month nursing led education and support program for parents of children with asthma (aged 5 to 12 years). Based on the transtheoretical model of behavior change, calls occurred on a weekly to monthly basis. (Outpatient hospital) | Telephone (Nursing) | Patient and carers |
| Mental health – insomnia  Tele-Insomnia program[69] (United States, 2010-2011) | The Tele-Insomnia program was a Veterans Health Administration (VHA) initiative in which CBT-I was delivered in a group format by telehealth. Veterans received six weekly sessions of group CBT-I. (Outpatient hospital) | Videoconferencing (Psychology) | Patients |
| Multiple chronic diseases  PACE inspired web-based communication intervention[70] (Canada, 2009-2010) | Participants were assigned to either an e-learning intervention; e-learning and workshop; or usual care (routine chronic disease follow up visits with primary care practitioner). The e-learning intervention was a self-directed program. The website presented content in the form of audio, stand-alone text, narrated text and representations of different doctor-patient encounters. (Primary care practices) | Internet-based (Multi-disciplinary) | Patients |
| Multiple chronic diseases  TERVA randomized controlled trial[71] (Finland, 2007-2008) | Health coaching was delivered via telephone and participants were contacted monthly. The coaching sessions covered a broad spectrum of topics such as learning about the participant specific chronic disease, intake of medications, recommended tests and services, and lifestyle changes. (Home-based) | Telephone (Nursing) | Patients |
| Multiple chronic diseases  PHYZ X 2U program[72] (Australia, 2017-2018) | The PHYZ X 2U program incorporated behavior change and structured exercise that aimed to increase access to allied health professionals for people with chronic disease living in rural communities. The program utilized multiple platforms, such as face to face consultations via a mobile clinic, weekly remote exercises via PhysiApp and health coaching via the phone or web. (Multi-disciplinary community health center) | Telephone and mobile app (Multi-disciplinary) | Patients |
| Osteoarthritis  Vett app[73](Norway, 2015) | The intervention involved 3.5 hour group-based education program using a an app. (Outpatient hospital) | Internet-based (Multi-disciplinary) | Patients |
| Inflammatory Arthritis program  Advanced Clinician Practitioner in Arthritis Care (ACPAC)-led inflammatory arthritis education program (RxEd program)[74,75] (Canada, 2012-2013) | A one-day education program provided by extended role practitioners for inflammatory arthritis, delivered in person and remotely. (Outpatient hospital) | Videoconferencing (Multi-disciplinary) | Patients |
| Hepatitis C  Hepatitis C Telemedicine Clinic[76] (United States, 2011) | Telemedicine clinics were provided from central Veterans Affairs (VA) hospital to three remote VA sites. This included a pharmacist-led initial group education session followed by individualized medication and disease management. (Outpatient hospital) | Telehealth (Pharmacy) | Patients |
| Chronic Obstructive Pulmonary Disease (COPD) | | | |
| Horizonte program[77] (Spain, 2013-2014) | Involved seven months of text messages, emails and phone calls to improve education and adherence to prescribed treatment and healthier behaviors. (Outpatient hospital) | Telephone and internet-based (Nursing) | Patients |
| COPD telemonitoring (RM system)[78] (Australia, 2006-2008) | A telemonitoring intervention was delivered to participants who had completed pulmonary rehabilitation, to augment nursing outreach services. The RM system included a laptop, blood pressure cuff, pulse oximeter, and other equipment for patients to monitor their vital signs. These were transmitted to a nurse who reviewed them on a weekly basis and contacted the patient if there were any issues identified. (Outpatient hospital) | Telehealth (Nursing) | Patients |
| Integrated Care Services supported by Information and Communication Technologies (ICS-ICT): multi-site program[79] (Spain, Norway and Greece, 2008-2012) | A 12-month ICT supported post 8-week cardiopulmonary rehabilitation program. In Spain, the ICT support included supervised/monitored exercise plan and exercise counselling. In Norway, ICT support included telephone calls, whereas in Greece, ICT included telemonitoring (SpO2). Platforms allowed bi-directional communication. (Outpatient hospital) | Internet-based and telephone (Multi-disciplinary) | Patients |
| Toronto Central LHIN Telehomecare program[80] (Canada, 2012-2016) | The intervention involved six months of health status monitoring (internet) and self-management education (phone) through health coaching. (Home-based) | Internet-based (Nursing) | Patients |
| INSPIRE-II Study (Coping Skills Training intervention)[81](United States, 2009-2013) | The intervention was a Coping Skills Training (CST) program which involved the delivery of cognitive-behavioral coping skills training to patients and partners over the telephone, weekly for 12-weeks, and bi-monthly for one month. (Outpatient hospital) | Telephone (Psychology) | Patients and carers |
| Telemonitoring program (COPD)[82,83] (Denmark, 2013-2014) | A telemonitoring program was delivered to patients with a recent COPD-related hospital admission, in addition to standard care. Patients self-monitored vital signs which were transmitted to a nurse call center who provided feedback on abnormal signs. Some measurements were obtained during a video consultation. (Outpatient hospital) | Telehealth (Nursing) | Patients |
| Tele-rehab program[84] (Australia, 2012-2014) | Participants developed a walking action plan and were supported by community nurses to complete the plan over 8 – 12 weeks. (Outpatient hospital) | Telephone (Nursing) | Patients |
| telEPOC program[85] (Spain, 2010-2012) | Intervention included two 30-minute education sessions on COPD, training in use of smartphone and telemonitoring software. Telemonitoring software included a daily questionnaire on symptom levels and daily data on O₂, HR, temp, steps, resp rate, which then triggered a particular response from nursing or medical staff. (Outpatient hospital) | Internet-based and telemonitoring (Nursing) | Patients |
| COPD telemonitoring service (TELESCOT Program)[86-88] (United Kingdom, 2008-2011) | The intervention involved installing telemonitoring equipment in the homes of patients with COPD for the purpose of self-management. Information was transmitted to a team of health professionals who responded as required. This usually involved a telephone call and undertaking a clinical assessment. (Home-based) | Telehealth (Multi-disciplinary) | Patients |
| CoMET program[89] (France, Germany, Italy and Spain, 2010-2015) | A home-based disease management intervention (COMET) delivered across 33 centers, which included a self-management program, home monitoring and an e-health telephone/web platform compared to usual care. (Home-based) | Telehealth (Multi-disciplinary) | Patients |
| u-Health service[90] (South Korea, 2008-2010) | Participants were allocated to one of three groups who received either a COPD monitoring service only or received monitoring and audio/phone support service or received monitoring, audio and visual/video. (Outpatient hospital) | Telehealth (Nursing) | Patients |
| Net-COPD program[91] (Denmark, 2015) | Patients were given a tablet computer with a web camera, microphone and measurement equipment (SpO2, spirometer, scales), measured indicators three times a week and readings were transferred to a call center at hospital. (Outpatient hospital) | Telehealth (Nursing) | Patients |
| COPD telehealth program[92](Netherlands, 2011-2013) | The program consisted of four modules which included activity coaching for ambulant activity monitoring and real-time coaching of daily activity behavior, a web-based exercise program for home exercising, self-management of COPD exacerbations via a triage diary on a web portal, self-treatment of exacerbations, and a teleconsultation. (Outpatient hospital) | Internet-based (Physiotherapy) | Patients |
| Telerehabilitation service for COPD patients[93](Norway, 2012-2016) | The service was a long-term telerehabilitation service for COPD patients comprising exercise training at home, telemonitoring and education/self-management. The service was offered as a two-year follow-up program by a physiotherapist. Equipment included a treadmill, a pulse oximeter and a tablet computer. Participants had weekly videoconference sessions with the physiotherapist. (Home-based) | Telehealth (Physiotherapy) | Patients |
| Chronic pain | | | |
| Chronic Knee Pain  Internet-Delivered Exercise and Pain-Coping Skills  Training Intervention[94] (Australia, 2014-2016) | The intervention involved three internet-delivered treatments which included education about exercise, pain management, emotions, healthy eating, CAM therapy, medications; interactive automated pain coping skills training (PCST) (eight modules 35-45mins each and practice PCS daily), and seven skype sessions with a physiotherapist over 12 weeks. (Home-based) | Internet-based (Physiotherapy) | Patients |
| Chronic Low Back Pain  Healthy Lifestyle intervention[95] (Australia, 2015) | The intervention group was offered a healthy lifestyle intervention involving brief telephone advice and offer of a clinical consultation followed by referral to a 6-month telephone-based healthy lifestyle coaching service. The approach was based on a formative evaluation which identified telephone services as the most preferred method by patients to support lifestyle change and weight loss. (Outpatient hospital) | Telephone (Physiotherapy) | Patients |
| Diabetes (including Gestational Diabetes) | | | |
| Mobile telehealth diabetes care[96-98] (United Kingdom, 2010-2011) | A mobile telehealth intervention using a phone application allowing participants to transmit diabetes-related clinical parameters to a nurse and receive clinical advice and feedback and education. (Outpatient hospital) | Internet-based (Nursing) | Patients |
| PEACH Trial[99](Australia, 2006-2009) | The intervention involved a nurse-delivered structured telephone coaching program to patients, focusing on lifestyle issues, medication adherence and dosing, self-monitoring of disease and support for goal setting. (Primary care practices) | Telephone (Nursing) | Patients |
| A tele-education intervention[100](Belgium 2012-2013) | Tele-education consisted of an online education-oriented communication between patient and diabetes educator with supervision of the endocrinologist, via the eConnecta platform. Diabetes Educators could suggest therapy modification, which the endocrinologist could approve or reject via eConnecta. (Outpatient hospital) | Internet-based (Diabetes Educator) | Patients |
| Telehealth diabetes self-management education[101] (United States, not reported) | Self-management educational module (culturally appropriate) housing participants’ health record and care plan. Care plan developed by telehealth nurse and participant during biweekly VCs. Information (clinical observation) and care plan were uploaded and transmitted to their provider. (Home-based) | Telehealth (Nursing) | Patients |
| TeleDiab 1 Study (Diabeo Software)[102] (France, 2007-2009) | Participants with type one diabetes were randomized to either a quarterly follow up and smartphone application (Diabeo software) or smartphone application (Diabeo software) with teleconsultations every two weeks. (Outpatient hospital) | Telemonitoring and internet-based (Medical) | Patients |
| LINE (mobile app)[103] (Taiwan, not reported) | All participant groups were provided with diabetes self-care information and emotional support through usual care. The mobile-based (intervention) group also used a communication app (LINE) to send and receive multimedia messages about diabetes self-management for 12 weeks. The telephone group received three to four phone calls (30-60mins duration) throughout the intervention period. (Outpatient hospital) | Telephone and internet-based (Diabetes educator) | Patients |
| Modified Diabetes Prevention Program (DPP)[104](United States, 2010-2015) | Over the 6-year study, three interventions were conducted which incorporated group classes from the DPP: a 16-week urban, in-person program (Urban-16); a 16-week rural, telehealth program (Rural-16); and a 12-week rural, telehealth program (Rural-12). Urban-16 and Rural-16 programs comprised 16 weekly sessions (the core period of the intervention), followed by six-monthly or bimonthly group sessions (post-core period). (Multi-disciplinary community health centers) | Videoconferencing (Multidisciplinary) | Patients |
| Telehealth diabetes self-management education (DSME)[105] (United States, not reported) | Intervention involved a comprehensive  DSME delivered remotely by a dietitian and nurse (CDE) to improve adherence to  guidelines. This included a remote retinal assessment. Of the thirteen sessions, ten were delivered remotely in a mix of group and individual sessions (via telemedicine), and three in person (all group sessions). (Multi-disciplinary community health centers) | Telehealth, videoconferencing and telephone (Multidisciplinary) | Patients |
| Proactive Interdisciplinary Self-Management (PRISMA) Program[106] (Netherlands, 2014-2015) | PRISMA involved participants setting personal goals and formulating action plans. Platform (e-Vita) had five items: health status, personal goals, educational modules (individualized based on health data), messages (emails), library (links to reliable information, short videos about pt. experiences using platform). (Primary care practices) | Internet-based (Nursing) | Patients |
| Living Well with Diabetes (LWWD)[107,108] (Australia, not reported) | An 18-month intervention which included 27 telephone coaching calls on a tapered schedule, using a motivational interviewing approach to promote behavior changes. Participants were given a workbook, pedometer and digital scales and were encouraged to achieve a weight loss of 5-10%, by improving dietary intake and physical activity. (Primary care practices) | Telephone (Dietitian) | Patients |
| Medical Guard Diabetes (MGD) system for people with type 1 diabetes[109] (Spain, not reported) | Medical Guard Diabetes system was an internet-based system that allowed automatic downloading of the SMGBLs to a secure website. Investigators could access data at any time and participants could changes in insulin dose, diet or exercise and make comments in text messages. Software and interface PC-meter were provided to those in the intervention group. (Outpatient hospital) | Internet-based (Multidisciplinary) | Patients |
| Telemonitoring diabetes management[110] (Greece, 2012-2014) | Telemonitoring participants received a 6-month diabetes management active support via a glucometer-connected modem which transmitted data to an endocrinologist daily, for review and feedback or action as required (via SMS or email). (Outpatient hospital) | Telemonitoring  and internet-based (Medical) | Patients |
| Telehealth remote monitoring (Care Innovations Guide)[111] (United States, 2013) | Participants in the intervention group attended a one-hour small group in-person training session covering use of the Care Innovations Guide (remote monitoring system) which connects to the glucose meter. Eight-four daily health sessions (developed by research team) were delivered sequentially through the Care Innovations Guide system. CDEs reviewed all data and contacted participants as required. (Outpatient hospital) | Telehealth (Diabetes Educators) | Patients |
| Telescot[112,113] (United Kingdom, 2011-2013) | Participants in the telemonitoring group were instructed to measure blood pressure (BP), blood glucose and weight monitors which were connected via Bluetooth technology to a remote secure server. Participants measured one fasting and one non-fasting blood glucose twice weekly and measured BP and weight weekly. Education included lifestyle modification, effects of medication change on glucose and BP, and when, or how to contact their healthcare advisor. Primary care nurses checked participant results weekly and organized treatment changes in line with national guidelines. (Primary care practices) | Internet-based (Nursing) | Patients |
| IDEATel project (114-118] (United States, 2000-2007) | Participants enrolled in the telemedicine intervention of the Informatics for Diabetes Education and Telemedicine (IDEATel) project participated in home televisits with nurse and dietitian educators every 4–6 weeks for 2–6 years. Behavior change goals (based on social cognitive theory) related to nutrition, physical activity, monitoring, diabetes health maintenance, and/or use of the home telemedicine unit were established at the conclusion of each televisit and re-assessed. (Home-based) | Videoconferencing (Multidisciplinary) | Patients |
| Group diabetes self-management education delivered via telehealth to participants at a rural health service site [119] (United States, not reported) | A group diabetes education class provided face to face and via telemedicine to people with diabetes. After a one-hour initial assessment, participants attended two three-hour hour group education sessions with a MDT (dietitian nurse and exercise physiologist, all CDEs). Telehealth participants were together (groups of 2-3) at their local rural health service. (Outpatient hospital) | Videoconferencing (Multidisciplinary) | Patients |
| Remote delivery specialty clinic model [120] (United States, not reported) | A type 2 diabetes specialty clinic model consisted of telephone and email contact, was compared to usual care (face to face endocrinology visit). Participants input data (BGLs, BP readings from last 2 weeks) to discuss during telehealth consults. (Outpatient hospital) | Telephone (Multidisciplinary) | Patients |
| Mobile Phone  and Web-Based Collaborative Care Intervention  for Patients with Type 2 Diabetes[121] (United States, 2007-2008) | The intervention involved a nurse practitioner delivered chronic care model for people with diabetes type 2 which integrated smartphone application, webpage, and telephone contact. (Outpatient hospital) | Internet-based (Nursing) | Patients |
| Rural telemedicine and DSME program (provided in-person)[122] (United States, not reported) | The intervention involved care management, telemedicine for access to specialist care, and diabetes prevention education. The nurse facilitated the telemedicine visits with the network endocrinologist and provided DSME (in groups or individual, in-person). A bilingual nurse care manager provided monthly patient telephone follow‐up, community referrals, assistance with medication and supplies, and assisted with telemedicine appointment scheduling. (Primary care practices) | Telehealth, videoconferencing and telephone (Nursing) | Patients |
| SINERGIA program[123] (Italy, not reported) | The SINERGIA model of management for people with type 2 diabetes of included telemedicine as one component. Participants had the option of mobile phone (SMS) or email for communication. (Outpatient hospital) | Telehealth (Multi-disciplinary) | Patients |
| Smartphone-based health coaching[124] (Canada, 2011-2015) | Involved a health coaching intervention with smart-phone self-monitoring (one hour of contact per participant each week), to support self-management of type 2 diabetes. (Multi-disciplinary community health center) | Internet-based (Health coaches) | Patients |
| Phone-based motivational interviewing for women with previous GDM[125] (Australia, not reported) | Intervention involved women with previous history of gestational diabetes who received a 6-month phone-based motivational interviewing program, and access to usual care. Phone calls were scheduled weekly for five weeks, then monthly for five months. (Outpatient hospital) | Telephone (Diabetes educators) | Patients |
| Web-based education and self-management platform for people with diabetes[126] (United States, 2009-2010) | Intervention included a web-based personal health record to which participants upload blood glucose levels regularly (every second day). Educational and motivational messages were supplied by an app (automated but based on inputs) and web-based access to nurse coordinator. A chat function enabled communication with nurses, a monitored discussion board and a focus on developing a peer network. (Multi-disciplinary community health center) | Internet-based (Nursing) | Patients |
| Telemedicine for diabetes care[127] (United States, not reported) | The intervention group were provided with specialist team diabetes management (CDE and endocrinologist) via videoconference (VC) once a week. During the video consultation, the patient was at their outpatient clinic with one health care provider (usually a nurse CDE). Electronic medical records were available and blood glucose data were faxed to the telehealth team, who interviewed the patient and provided advice regarding the management of diabetes via VC. (Primary care practices) | Videoconferencing (Multi-disciplinary) | Patients |
| Diabetes Prevention Program (DPP)[128] (United States, 2009) | A DPP intervention was delivered to an on-site group and simultaneously through telehealth video conferencing to a second group in a remote frontier community. The intervention included 16-weekly sessions plus six monthly after-care sessions. Each session lasted approximately one hour. Participants at the in-person site and those at the remote (VC) site could see and hear each other. (Outpatient hospital) | Videoconferencing (Multidisciplinary) | Patients |
| Pedometer-based physical activity intervention with telephone mediated motivational interviewing[129] (Belgium, not reported) | Intervention involved an initial face to face session, the provision of a pedometer and  seven motivational interview sessions via telephone over a 24-week period with follow-up 12-months post study. The intervention was informed by the Diabetes Prevention Program, cognitive behavioral therapy, the First Step Program and motivational interviewing. Participants were asked to wear pedometer daily and record steps and other physical activity in a logbook at the end of each day. (Outpatient hospital) | Telephone (Psychology) | Patients |
| Telephone diabetes self-management education and coaching[130] (Australia, not reported) | In addition to usual diabetes care, intervention group participants received 6 months of telephone coaching. Diet, exercise and treatment goals were the focus of the sessions. (Outpatient hospital) | Telephone (Dietitian) | Patients |
| Telephone delivered diabetes self-management education[131] (Singapore, not reported) | Intervention included monthly telephone calls for the first 3 months, three personalized education modules (on diabetes, cholesterol and blood pressure management and symptoms related to complications) and lifestyle modification education. A follow-up call 3 months after the last call was made, to monitor progress and provide support. (Outpatient hospital) | Telephone (Nursing) | Patients |
| Home telehealth for diabetes and hypertension[132] (United States, 2005-2006) | Intervention included a telehealth home device to facilitate close surveillance with nurse care management over a 6-month period. Participants entered blood glucose (BG) and BP levels into the device which used telephone connected to transmit data to the study nurse who reviewed and implemented interventions as required. (Outpatient hospital) | Telehealth (Nursing) | Patients |
| Telehealth-delivered diabetes self-management education and support[133] (United States, 2017-2018) | The intervention included one in-person home visit and follow-up weekly virtual synchronous one-on-one diabetes self-management education and support DSMES sessions via electronic tablet (synchronous VC) for four additional weeks. Participants continued to access their usual care throughout the intervention (Outpatient hospital) | Internet-based (Nursing) | Patients |
| Risk factors | | | |
| Obesity/Overweight  MOVE! Program[134] (United States, 2008-2010) | The MOVE! Weight management program was delivered by a multi-disciplinary health care team through 12 weekly group classes using videoconferencing. The program targeted veterans with a BMI greater than 25 kg/m^2^. Content focused on diet, physical activity and behavioral modifications. (Outpatient hospital) | Videoconferencing (Multi-disciplinary) | Patients |
| Physical inactivity  Healthy4U[135] (Australia, 2016-2017) | A 30 min group education session focused on self-management and lifestyle modification (drew on self-determination theory) was delivered, followed up by integrated telephone motivational interviewing and cognitive behavioral therapy delivered in eight 30 min sessions over 12 weeks. (Outpatient hospital) | Telephone (Multi-disciplinary) | Patients |
| Psychological distress  Telephone and web-based coping skills training program[136] (United States, 2013-2015) | Weekly telephone sessions were delivered over six weeks, addressing relaxation, communication, and self-management for patients discharged who had been mechanically ventilated (critically ill). This was compared to an education program. (Outpatient hospital) | Telephone (Psychology) | Patients and carers |
| Overweight  Lifestyle, Eating and Activity Program (LEAP Beep)[137] (United Kingdom, 2010) | All participants completed a 12-week dietitian-led weight management group addressing the risk factor of being overweight. Participants were then given daily targets for fruit, vegetable and breakfast consumption, and a daily steps target (and pedometer). Twice a week, participants were sent text reminders to text the practitioner their data – and they were to send back one weekday and one weekend message. A response was then provided. (Home-based) | Text-messaging (Dietetics) | Patients |
| Lifestyle prevention  GLOW study[138] (United States, 2014-2017) | The core lifestyle intervention consisted of two in person and 11 (weekly) telephone sessions on behavioral strategies to improve weight, diet, and physical activity, and stress management to help women achieve weight gain at the lower limit of the Institute of Medicine (IOM) guidelines range for total GWG. Lifestyle intervention drew on the principles of the Diabetes Prevention Program. (Outpatient hospital) | Telephone (Dietetics) | Patients |
| Obesity  Mobile phone app for patients who are overweight or obese[139] (Switzerland, 2016-2018) | A digital communication system connected patients remotely with their dietitian who had all the tools required for remote counseling. All features of the app were modeled on typical activities in a dietitian’s everyday practice and included chat-like communication with dietitians, group chats for support from peers, dietitian’s profile to create a more personal connection, a photo-based food log, activity and weight logs, a goal scorecard, showing past goals and future options, a content database, and feedback to the dietitian. (Home-based) | Internet-based (Dietetics) | Patients |
| Hypertension  Self-Help Intervention Program for High Blood Pressure (SHIP-HBP)[140] (United States, 2003-2008) | The intervention had three components and included a structured psycho-behavioral education, home BP monitoring with a tele-transmission system; and telephone counseling delivered by a bilingual nurse. After a 6-week education and a 6-week test period for home BP monitoring, telephone counseling was begun and continued for 12 months. (Home-based) | Telehealth (Nursing) | Patients |
| Obesity  Self-management and educational support in severely obese patients (EVOLUTION)[141] (Canada, 2013-2015) | Trial included three groups: in person self-management group intervention delivered by a multidisciplinary team designed to educate patients regarding proper diet and exercise and weight management skills, a web based self-management group with similar content to the in person intervention but delivered solely in an online format, and a control group who received education material. (Multi-disciplinary community health center) | Internet-based (Multi-disciplinary) | Patients |
| Obesity  Interactive Diary for Diet Management (DAI)[142] (Italy, 2008-2009) | The intervention involved text messages to and from dietitians focusing on nutrition education and management of dietary intake. Proactive phone calls were delivered weekly to reinforce nutrition and physical activity messages. (Outpatient hospital) | Internet-based (Dietetics) | Patients |
| Stroke prevention  Telehealth Stroke Education[143] (United States, not reported) | The intervention involved a stroke prevention education session for elderly persons at risk of stroke delivered in person and via videoconferencing. (Multi-disciplinary community health center) | Videoconferencing (Nursing) | Patients |
| Lifestyle prevention  SHINE (Diabetes Prevention Program) study[144] (United States, 2009-2010) | DPP lifestyle intervention was delivered by telephone, either individually (IC) or in conference calls (CC), up to eight participants per call. Sessions included education, augmented by monthly coaching (dietitian) sessions. For both interventions, educators followed scripts per DPP materials for the 16-session core curriculum, which included goal-setting, self-monitoring, diet/activity modification and problem-solving. Topics were presented weekly for the first five weeks, then monthly for one year. A modified 12-session curriculum (monthly calls) was used by educators during year two. (Primary care practices) | Telephone (Dietetics) | Patients |

**Table S6. Research evidence of included programs**

| **Name of health program** | **Research Design (follow up periods)** | **Sample size (attrition rate n %)** | **Mean age years (SD) or years (range)** | **Female n (%)** | **Primary outcome** | **Secondary outcome(s)** | **Key results** | **Effect (Positive/Neutral/Negative – RCTs only) with implications** |
| --- | --- | --- | --- | --- | --- | --- | --- | --- |
| Cancer | | | | | | | | |
| Telephone interpersonal counselling (TIPC) and Supportive Health Education (SHE) | Randomized controlled trial[46]  (baseline, 2, 4 and 6 months) | TIPC = 116 pts and 116 carers; SHE = 114 pts and 114 carers (n=31; 27%) | TIPC pts: 49.64 (10.38), SHE pts: 51.8 (10.49), TIPC carers: 45.22 (14.43), SHE carers 43.53 (12.92) | 230 (100%) | Psychological distress (Patient Reported Outcome Measurement Information System (PROMIS) | Symptoms (General Symptom Distress Scale); social isolation | TIPC was superior to SHE for the management of depression, SHE was more effective in managing anxiety, social isolation and cancer related symptoms. Both interventions worked as designed. | Neutral  Both telephone-delivered education and counselling intervention for women with breast cancer and their carers were found to be effective. |
| Telephone-based symptom management (TSM) | Randomized Controlled Trial[47]  (2 and 6 weeks) | Intervention n=51 patients, n=51 caregivers; control n=55 patients, n=55 caregivers (n=56, 26.4%) | Intervention patients 63.5 (7.7), caregivers 56.3 (14.1); control patients 62.0 (8.2), caregivers 56.8 (13.8) | Intervention patients n=28 (54.9%), caregivers n=37 (72.6%); control patients n=28 (50.9), caregivers n=40 (72.7%) | Anxiety / depression (Patient Health Questionnaire (PHQ-8), Generalised Anxiety Disorder (GAS-7)); pain (Brief Pain Inventory Short Form); fatigue (Fatigue Symptom Inventory) | Self-efficacy (survey); Caregiver Reaction Assessment | Intervention had no effect on anxiety and depression symptoms, pain, fatigue or breathlessness when compared to control. No effect on self-efficacy in the patient intervention group, however, impacts on self-efficacy in managing emotions for caregivers in the intervention group. | Neutral  Intervention may have some benefits for caregivers, but not for patients. Further research required in a larger population sample. |
| Education or Motivational-Interviewing-Based Coaching | Randomized controlled trial[48]  (baseline and 6 months) | Education n=103; Coaching n=105; Control n=109 (n=91, 28.6%) | Education 62.5 (11.2); Coaching 61.8 (11.3); Control 58.7 (11.5) | Education 4 (5%); Coaching 10 (16%); Control 9 (10%) | Attitudinal barriers (Barriers Questionnaire (BQ)) | Pain management (Brief Pain Inventory); Functionality (SF-36); QoL (FACT-G) | Attitudinal barrier scores did not change over time among groups. Patients randomized to the coaching group reported significant improvement in their ratings of pain related interference with function, as well as general health, vitality, and mental health. | Positive  Although additional research is required, coaching using the telephone may be a useful strategy to help patients decrease attitudinal barriers toward cancer pain management and to better manage their cancer pain. |
| Cardiovascular disease | | | | | | | | |
| Virtual cardiac rehabilitationprogram (vCRP) | Qualitative study[49] | 19 participants | Not reported | Not reported | Patient experience (semi-structured interviews) | Not reported | Five major themes; (1) accessibility - understood to be accessible, convenient and effective, (2) making health choices - greater awareness and motivation to manage, (3) surveillance, (4) barriers to participation - lack of time, infrequent access to the internet or computer, lack of motivation, (5) perceptions of vCRP - wanted program to be longer | vCRP understood to be a accessible, convenient and effective way to delivery cardiac rehabilitation services, particularly for those located in rural and remote settings who otherwise lack ready access to traditional services. |
|  | Randomized controlled trial[50]  (baseline, 4 months, and 16 months) | Intervention n=38; control n-=40 (attrition rate not reported) | Intervention  61.7 (51.3-65.2); control 58.4 (52.8-64.7) | Intervention n= 4 (10%); control n=8 (20%) | Physical activity (steps, sedentary time, 6MW) | Blood pressure; Feasibility | Participants in the intervention group had a greater increase in maximal time on the treadmill by 45.7 seconds (95% CI: 1.04, 90.48) compared to the usual care group over the 16 months (p=0.045). Intervention perceived to be accessible, convenient and effective. | Positive  Cardiac rehabilitation program delivered exclusively through the internet to patients is safe and effective and can lead to improvements in exercise capacity and reductions in CVD risk. |
| CHANGE intervention | Randomized trial[51] | CHANGE intervention n=182 | CHANGE intervention 56 (12) | 126 (69) | Rate of medication intensification | HbA1c;  Blood pressure; LDL cholesterol | (1) Enhance medication adherence; (2) low rates of appropriate medication intensification (i.e. clinical inertia) | Neutral  Education via telephone improves medication adherence but does not influence intensification of treatment by the relevant medical officer |
| Collaborative Care program for depression following a coronary artery bypass graft | Economic evaluation of a randomized controlled trial[52]  (12 months) | Intervention n=150; control n=152 (n=189, 62.6%) | Intervention 67.0 (10.4); control 58.8 (10.1) | Intervention 37%; control 41% | Health-related Quality of Life (SF-26); Cost analysis | Depression (Hamilton Rating Scale for Depression) | At 12 months, those receiving the intervention had $2068 lower but statistically similar estimated  median costs compared to control (P=0.30). Incremental cost-effectiveness ratio of the intervention was −$9889 (−$11,940 to −$7838) per additional quality-adjusted  life-year (QALY), and there was 90% probability it would be cost-effective at the willingness to pay threshold of $20,000 per additional QALY. | Positive  Telephone delivered nursing intervention post CABG proved to be cost-effective. |
| Illawarra and  Shoalhaven Healthy Heart Project | Randomized controlled trial[53]  (baseline, 6 weeks, and 6 months) | Intervention n= 109; control n = 113 (n=21, 9.5%) | Intervention 66.9 (9.0); control 67.1 (11.5) | Intervention n 9 (27.5%); control n= 35 (31.5%) | Physical activity (Active Australia Questionnaire) | Anxiety / depression (Kessler 6); self-efficacy (self-efficacy for exercise scale) | After the 6-week intervention, improvements in total physical activity (PA) time, total PA sessions, walking time and walking sessions were all significantly greater in the intervention group compared to control. Significant improvements in self-efficacy, outcome expectancies, cognitive and behavioral self-management strategies in the intervention group at 6 weeks, and were maintained at 6 months for outcome expectancies and self-efficacy. | Positive  For patients who are unable to participate in standard Cardiac Rehabilitation, a pedometer-based phone intervention may successfully increase their physical activity and walking levels. |
| Synchronous telehealth program for chronic CVD | Case control and economic evaluation[54] | n= 576 intervention  n= 1178 control | 64.6 (16.3) intervention  64.5 (16.1) control | 220 (38.2%) intervention  461 (39.1%) | Cost | Hospitalization rate  Length of stay | The telehealth program was associated with lower total costs, lower rate of hospitalizations, and shorter hospitalization length of stay in patients with chronic CVD during the 2-year follow-up period. The intervention itself was more cost-effective than the usual approach to delivering care. | The telehealth program did reduce the rate of hospitalization of people with chronic CVD and therefore saved costs associated with acute hospital care. |
| Home-based telerehabilitation | Randomized controlled trial[56]  Economic evaluation of an RCT[55] | Intervention n=24; control n=29 (n=4, 7.6%) | Intervention 68 (14); control 67 (11) | Intervention n=5 (21%); control n=8 (28%) | Physical activity (6MWD, TUGT, 10-min walk test, strength (kg) grip, BOOMER, RUIS); cost-effectiveness | QoL (EQ-5D, VAS, Utility, MLWHFQ) | Non-inferiority of the telerehabilitation program in terms of primary and secondary outcomes and participant satisfaction  Telerehabilitation less costly and as effective as centre-based heart failure rehab. The program costs per participant were $1,778 and $2,906 respectively. Total health care costs per participant over the 6 months (inclusive of program and heart failure readmission | Neutral  Telerehabilitation delivered in the home is no less effective than that delivered in care settings. No difference in utilities between the two programs, but the telerehabilitation group had a high probability of being cost saving compared with the control group. |
| The Teledialog Project (CVD) | Economic Evaluation of a randomized controlled trial[57]  (12 months) | n=75 intervention; n=76 control (n=32, 21.2%) | 62.46 (12.3) intervention; 62.67 (11.72) control | n=16 (22.2%) intervention; n=14 (20.3%) control | Quality of life (SF-36); costs | Not reported | Rehabilitation activities were the same between intervention and control, however, there was more contact with the physiotherapist in the intervention group. Mean total cost per patient was €1,700 higher in the intervention group (not statistically different). Incremental CU ratio was more than €400,000 per QALY gained. | Neutral  Telemonitoring program for cardiac rehabilitation may increase the patient's participation in rehabilitation activities, reduce the cost of transport, and reduce the number of emergency physician visits. However, the program was not cost-effective compared to the control. |
| Telemonitored exercise-based Cardiac Rehabilitation (TCR) | Cohort (one group follow up pre and post)[58] | 34 (n=10, 29.4%) | 58 (10.2) | n=6, (17.7%) | V_O2peak_ ; muscle power (Nottingham Leg Extensor Power Rig); muscle strength (Good Muscle Strength Metitur) | HRQoL (SF-36) | A significant increase in V_O2peak_ of 10%, in muscle endurance of 17%, in muscle power of 7% and in muscle strength of 10% after the TCR program at 12 weeks. HRQoL was significantly improved by 19% in the physical and 17% in the mental component scores at 12 weeks. No improvement found at a six week follow up. | A 12-week TCR program improved physical capacity, muscle endurance, muscle strength, power and HRQoL post-intervention, but no improvements found at six months follow up. |
| Nurse-Led Collaborative Management using Telemonitoring | Randomized controlled trial[59]  (baseline and at 6, 12, 18, and 24 months) | Usual Care (UC) n=19; Self-management education (SM) n=20; Collaborative Management using telemonitoring (CM) n= 20; (n=13, 22%) | UC 74.5 (12.1); SM 69.4 (12.9); CM 70.5 (13.3) | UC 9 (47.4%); SM 3 (16.7%); CM 10 (50%) | Quality of life (QoL) (Minnesota Living with Heart Failure Questionnaire MLWHFQ) | Rate of readmission (an unplanned overnight stay due to heart failure symptoms); Self-efficacy (Chronic Disease Self-Efficacy Scale CD-SES) and self-care (European Heart Failure Self-Care Behavior) | QoL scores improved significantly in the CM group, compared to UC at 18 and 24 months, and at 18 months when compared to the SM group. QoL significantly improved at 6, 12, 18 and 24 months in only the CM group. CM group also had a significant improvement in self-efficacy. Both the SM and CM groups had a reduced rate of hospitalization compared to the UC group. | Positive  A collaborative model of care including education and telemonitoring can help improve QoL in patients with heart failure and may help reduce hospital readmissions due to heart failure. |
| Community Outreach Heart Health and Risk Reduction Trial (COHRT) | Randomized controlled trial[60]  (baseline, 2 weeks and 6 months) | Intervention n=415; control n=268 (n=62, 9.1%) | Intervention 59.27(0.43); control 58.61 (0.53) | Intervention n=202 (48.9%); control n=132 (49.4%) | Adherence to Health Canada guidelines for exercise, diet and smoke free living. | Risk factors for CVD (blood pressure, LDL and HDL cholesterol) and Framingham index. | A larger proportion of telehealth participants compared to control participants reported adherence to exercise and diet after treatment and at a 6-month follow-up. All risk factors decreased significantly for telehealth participants and controls at 6-month follow-up. Telehealth participants demonstrated greater decreases in systolic and diastolic blood pressures but not total/high-density lipoprotein cholesterol or 10-year absolute risk of coronary heart disease | Positive  Telehealth counseling augments therapeutic lifestyle change in individuals at risk of a cardiovascular event compared to brief intervention counselling. |
| ProActive Heart trial | Randomized controlled trial[61]  (baseline and 6 months) | Intervention n=215; control n=215 (n=93, 21.6%) | Intervention 61.3 (11.3); control 59.9 (11.1) | Intervention n=52 (24.2%); control n=57 (26.5%) | HRQoL (SF-36); physical activity (Active Australia Survey) | Self-reported dietary intake and alcohol intake (Food frequency questionnaire); BMI; smoking status | In the intervention group, significant effects were found in some domains of HRQoL, including mental component (p=0.02), social functioning (p=0.04), and role-emotional (p=0.03) when compared to usual care. The intervention group was more likely to meet recommended levels of physical activity (p=0.02), BMI (p=0.05), vegetable intake (p=0.04) and alcohol consumption (p=0.05). | Positive  A telephone delivered secondary prevention program for cardiovascular disease can improve health outcomes in participants. |
|  | Randomized controlled trial[62]  (baseline and 6 months) | Intervention n=215; control n=215 (n=105, 24.4%) | Intervention 62 (11); control 59.7 (10.4) | Intervention n=30 (21.3%); control n=32 (20.5%) | Anxiety and depression (Hospital Anxiety and Depression Scale) | None reported | A statistically significant p=0.04, -0.7 (-1.4, -0.02) reduction in mean anxiety scores in the intervention group compared to the control group. Mean depression scores between the two groups did not reveal significant results. | Positive  Some positive psychological effects of rehabilitation programs in cardiac populations. Results of the study support the implementation of tele-health in chronic disease populations. |
| Tele-nutrition intervention for men at risk of CVD | Pilot Randomized controlled trial[63]  (baseline, 6 weeks, and 12 weeks) | Intervention n=29; control n=30 (n=3, 5.1%) | Intervention 58.6 (8.1); control 59.3 (7.4) | n=0 (0%)^  ^male only intervention | Feasibility (satisfaction and program adherence using survey) | Weight loss (anthropometric and body composition measures); Dietary intake (Healthy Eating Index-2015 index) | 92% of participants agreed or strongly agreed that they were 'satisfied with the program'. Participants in the intervention group lost a mean total of 8.3 kg (6.2% ± 3.5) and those in the control group lost a mean total of 5.0 kg (4.3% ± 4.3). For intervention and control groups, intakes improved for total fruit (p = 0.05), whole grains (p = 0.004) and fatty acid ratio (p = 0.002) and lowered for sodium (0.01), refined grains (p = 0.04), added sugars (p = 0.01) and saturated fats (p = 0.002). | Positive  Tele-nutrition programs are effective in reducing caloric intake, body weight and improving diet quality in men at risk of CVD |
| Other chronic diseases | | | | | | | | |
| Acquired Brain Injury  Move it to improve it program | Randomized controlled trial[64]  (baseline and 20 weeks) | Intervention n=30; control n=30 (n=9, 15%) | 11;11 months (2;6 months) (children) | Not reported | Functional strength | Walking endurance (6MWT); high level mobility (High level Mobility Assessment Tool); functional mobility (Timed Up and Go Test); habitual physical activity (accelerometer device); mobility limitations (28-item Mobility Questionnaire); acceptability and feasibility (questionnaire) | For functional strength, there was a significant difference between intervention and control. For endurance, there was no significant difference between intervention and control. In terms of feasibility, there were mixed results: majority of parents in Intervention group reported benefits of ICT intervention but that the ICT frequency and duration was too long. | Neutral  Although significant improvements in ICT group, results did not exceed minimal clinical sign difference. |
| Telehealth program (multiple chronic diseases) | Cluster randomized controlled trial[65]  (12 months) | n=845 intervention; n=728 control  (n=372, 23.6%) | 70.11 (11.81) intervention; 70.61 (11.78) control | n=350 (41.4%) intervention; n=290 (29.8%) control | Health-related quality of life (SF-12 and EQ-5D); anxiety (6-item Brief State-Trait Anxiety Inventory) and depressive symptoms (10-item Centre for Epidemiological Studies Depression Scale) | N/A | In the intention to treat analyses, differences between groups were small and non-significant for all outcomes at 12 months. | Neutral  Telehealth was not effective or efficacious compared with usual care. There was no improvement in quality of life or psychological outcomes for patients with COPD, diabetes or heart failure. |
| Parkinson’s Disease  mHealth-mediated exercise program | Randomized controlled trial[66]  (baseline, 3 months, 6 months and 12 months) | Intervention n=26; control n=25 (n=7, 13.7%) | Intervention 64.8 (8.5); control 63.3 (10.6) | Intervention n=11 (42.3%); control n=12 (48%) | Physical activity (StepWatch Activity Monitor) | Health-related Quality of life (PDQ-39); Walking capacity (6MWT); Adherence, safety and acceptability (satisfaction survey) | Mean change in daily steps at 12 months was 102.6 steps (95% CI= -888 to 1092) in the mHealth compared with 159 steps (95% CI= -878 to 1195) in the control group. The difference was not statistically significant. Estimated mean change in the number of moderate-intensity minutes per day at 12 months was 17.4 minutes (95% CI = −17.2 to 52.0) and 12.3 minutes (95% CI = −23.9 to 48.5) for the mHealth and active control groups, respectively. Not statistically significant | Neutral  Both home exercise program (intervention) enhanced with mHealth technology and control implemented for people with Parkinson’s may prevent decline in exercise due to the disease. |
| Telephone Asthma Program (TAP) | Randomized controlled trial[67]  (12 months pre-intervention and 12 months post-intervention) | n=190 intervention; n=172 control (n=11, 3.0%) | Not reported | n=70 (37%) intervention; n=68 (40%) control | Carer and Patient Quality of Life | Urgent Care Events; Asthma control; self-management behaviors | Parental asthma related QOL scores improved by a mean of 0.67 units (95% CI 0.49 to 0.84) in the intervention group and 0.28 units (95% CI 0.10 to 0.46) in the control group (statistically significant). No difference was found in the child’s QOL or in the mean number of urgent care events. | Positive  A telephone coaching program can improve parental QOL, however, no difference for child QOL or urgent care attendance. |
|  | Randomized controlled trial[68] | As above | Age of parents not reported. Children were 5-12 years | Not reported | Goals set (Controller medications, Asthma Action Plan, Rescue meds, planning visits) | Not reported. | Increased uptake of the 4 targeted desired asthmas care behaviors, especially use of controller medications, asthma action plans and use of rescue medications, was found in intervention group compared to control. | Positive  Nurses telephone coaching was successful in promoting asthma self-management behaviors in parents of children with asthma when compared to control. |
| Mental health – insomnia  Tele-Insomnia program | Cohort (one group pre and post follow up)[69] | 214 veterans | 57.8 (12.9) | n=6, (3%) | Insomnia Severity Index (ISI) | Sleep diary | Participants reported significantly decreased insomnia symptoms (ISI), with a mean decrease of 4.8 – 6.3 points (p < 0.0001). Participants reported significant improvements in all sleep diary variables (all p < 0.01) with the exception of total sleep time (p = 0.09). | Results indicate that telehealth delivery of CBT-I can produce clinically significant improvements in a wide range of patients. |
| Multiple chronic diseases  PACE inspired web-based communication intervention | Randomized controlled trial[70]  (baseline, 4 months and 16 months) | E-learning n=110; E-learning and workshop n=104; control n=108 (n=99, 31%) | E-learning 57.0 (8); E-learning and workshop 60.0 (10);  Usual care 58.1 (10) | n=92 (41.1%) | Proportion of patients meeting treatment suggested guidelines (BP, HbA1c, Lipids) | Not reported. | Patients in the e-learning were 1.42 times more likely to meet targets compared to usual care [95% CI: 1.00–2.00], a statistical difference was not seen in the combined group. | Positive  A web-based communication intervention positively impacted reaching treatment targets for primary care patients. However, the high cost and low attendance of workshops render them an unattractive tool for patient communication skills interventions in this setting. |
| Multiple chronic diseases  TERVA randomized controlled trial | Randomized controlled trial[71]  (baseline and 12 months) | Intervention n=1034; control n=501 (n=314 20.5%) | T2DM intervention 64.6 (9.4), control 65.6 (9.5); CAD intervention 65.4 (9.4), control 66.0 (8.6); CHF intervention 67.3 (7.9), control 62.4 (7.7). | n=611 (40%) | Blood pressure; weight loss; total cholesterol, LDL | For CHF, improved or maintained NYHA class; for T2DM HbA1c | Results were inconclusive. The participants in the intervention arm with diastolic blood pressure initially above the target level decreasing to 85mmHg or lower was 48% in the intervention arm and 37% in the control arm. The reduction was statistically significant between the two groups. | Neutral  Limited evidence from the study to support tele coaching intervention for chronic disease management. |
| Multiple chronic diseases  PHYZ X 2U program | Cohort (one group pre and post follow up)[72] | 63 participants (n=39, 61.9%) | 56.9 (15.7) | n=29 (47%) | Goals set (goals attained) | QoL (EQ-5D-3L); Weight loss (kgs); rate of hospitalization; exercise self-efficacy | In terms of goals attained, 59% of participants reported attaining at least one of their health-related goals. Participants with multi-morbidity had higher goal attainment compared with people without multimorbidity. Participants who attained at least one goal received significantly more health coaching minutes and more phone calls than participants who did not achieve any goal. | The program improved health related outcomes including attainments of health-related goals and increased the amount of weekly exercise for people with chronic diseases in rural Australia. |
| Osteoarthritis  Vett app | Cohort (one group pre and post follow up)[73] | 12 (n=1, 8.3%) | 65 (61-70) | n=10 (83.3%) | Acceptability | Goals attained (Self-Report Habit Questionnaire); utility estimates | Perceived goal achievement increased for all participants. The intervention was deemed feasible and well-accepted among participants | Technology-based tools can promote behavioral change and increase adherence for changes. The app had high acceptability, usability and utility. |
| Inflammatory Arthritis program  Advanced Clinician Practitioner in Arthritis Care (ACPAC)-led inflammatory arthritis education program (RxEd program) | Pre- and post- intervention design[74]  Pre- and post-intervention survey and qualitative interviews[75] | Remote n=87; in-person n=36 | Remote 58.6 (13.3); in-person 56.80 (13.09) | Remote n=76 (87.4%); in-person n=22 (91.7%) | Attitudes and usefulness of the program (semi-structured interviews); self-efficacy; knowledge about arthritis; coping efficacy; intrusiveness of illness on life (13-item illness intrusiveness scale); Effective Musculoskeletal Consumer Scale (17-item scale) | Disease activity (rapid assessment of disease activity index) and disability (Health Assessment Questionnaire (HAQ-8)) | Delivering the program via videoconferencing technology was feasible, well-received and as effective as delivering in person. Both groups showed immediate effect in improved arthritis which diminished over six months post program. | No difference in outcome measures between remote and in-person evaluation/feasibility findings for program delivery. |
| Hepatitis C  Hepatitis C Telemedicine Clinic | Survey study[76] | 18 participants | Not reported | Not reported | Patient satisfaction (survey) | Travel time | There was a reduction in distance travelled and time spent accessing pharmacy care of disease. 82% of survey respondents preferred their future Hep C Clinic visits to be conducted via telemedicine. 78% of respondents would prefer all future appointments for any disease state to be telemedicine. | Telemedicine has potential to improve ease of access and opportunities for patients accessing health services from remote locations. |
| Chronic Obstructive Pulmonary Disease (COPD) | | | | | | | | |
| Horizonte program | Cohort (one group pre and post follow up)[77] | 114 (n=21, 18.4%) | 69.6 (9.1) | n=21, 18.40% | COPD assessment test (CAT) | COPD exacerbations; adherence | Median change in total exacerbations (post-pre study) was -1 (IQR: -2,0); 41.9% significantly improved CAT status (CAT ↓≥2 points);no significant change in pulmonary function; 66.7% of patients who were non-compliant at baseline became compliant during the intervention. | For high-risk COPD patients, involvement in a remote support program (phone and email) based on disease-specific self-management principles resulted in half experiencing an improvement in self-reported health status, and a reduction in number of exacerbations. Those with worse health status at baseline benefitted the most. |
| COPD telemonitoring (RM system) | Pilot randomized controlled trial[78]  (baseline and 12 months) | n=22 intervention; n=22 control (n=8, 18.2%) | 68 (9) intervention; 70 (10) control | n=12 (54.5%) intervention; n=12 (54.5%) control | Hospital admissions; inpatient bed days; quality of life (SF-36) | 6MWD; patient acceptance | No differences were found between primary and secondary outcomes in intervention and control groups. | Neutral  Although the addition of telemonitoring to standard care was feasible, it did not reduce hospital admissions or improve quality of life. |
| Integrated Care Services supported by Information and Communication Technologies (ICS-ICT): multi-site program | Multi-country non-randomized controlled trial[79]  (baseline, 8 weeks and 12 months | Spain intervention n=27, control n=96 (n=46, 37.4%); Norway intervention n=28, control n=27 (n=18, 39.1%); Greece intervention n=25; control n=57 (n=42, 51.2%) | Spain intervention 64 (6), control 66 (9); Norway intervention 65 (8), control 62 (7); Greece intervention 65 (5), control 67 (8) | Spain intervention 8%, control 10%; Norway intervention 64%, control 45%; Greece, not reported | Physical activity (6MWT) | Quality of life (Saint George’s Respiratory Questionnaire); dyspnea symptoms (modified Medical Research Council dyspnea scale) | Spain ICT group maintained aerobic activity capacity at 12 month follow up compared with usual care. This result was not seen in Norway or Greece, outcomes for intervention and control were similar | Neutral  ICT approach needs to be specific to the organization, client group and technology availability. |
| Toronto Central LHIN Telehomecare program | Cohort analytic (one group pre and post follow up)[80] | Over 3000 patients enrolled between 2012-2016 | Not reported | Not reported | Rate of hospitalization | Patient, caregiver and health provider satisfaction | Reduction in Emergency Department visits by 46% whilst on program; hospital admissions reduced by 63% compared to six months prior (baseline). There benefits continued following the program.  General satisfaction with the program: 88% patients felt that nurse understood what was important to them and 94% would recommend the program to others. | Evidence for ICT support approach that can be applied in a region-wide context. |
| INSPIRE-II Study (Coping Skills Training intervention) | Randomized controlled trial[81]  (baseline, 16 weeks and up 4.4 years) | Intervention n=162; control n=164 (n=28, 8.6%) | Intervention 65.6 (7.9); control 66.6 (8.7) | Intervention n=61 (37.7%); control n=66 (40.2%) | Quality of Life (SF-36, Beck Depression Inventory II, STAI), COPD-related hospitalization | Somatic Quality of Life (PQLS, SOBQ); Brief Fatigue Inventory; St George’s Respiratory Questionnaire; Pulmonary Function, Physical Activity (6MWT, Charlson Medical Comorbidity Index) | The intervention group had greater improvements in psychological QoL when compared to controls (p=0.001), less depression and anxiety and overall better mental health and social functioning. There were no improvements in COPD-related hospitalization. | Positive  The telehealth intervention resulted in improvements in quality of life and functional capacity. |
| Telemonitoring program (COPD) | Survey[82] | n=279 | 74.0 (9.0) | 177 (68.9%) | Reason for declining participation | Not reported | Reasons for declining participation included: technical reasons (n=53), personal reasons (n=164, wish to continue at the outpatient clinic (n=17), do not want to participate in research (n=23), death (n=10), and other/not reported (n=12). | Personal reasons and concerns around technology were key reasons for declining participation. |
|  | Randomized controlled trial[83]  (baseline and 6 months) | n=141 intervention; n=140 control (n=33, 12.6%) | 69.8 (9.0) intervention; 69.4 (10.1) control | n=86 (61%) intervention; n=63 (45%) control | Hospital admissions for COPD | All-cause hospital admissions; time to first hospital admission; number of hospital visits | No difference was found in hospital admissions for COPD between the groups (P=0.74). | Neutral  Telemonitoring in addition to standard care, did not reduce COPD related hospital admissions. |
| Tele-rehab program | Randomized controlled trial[84]  (baseline, between 8-12 weeks and after 8 weeks) | Intervention n=35; control n=30 (n=25, 38.5%) | Intervention 68 (9.9); control 70 (6.8) | Intervention n=19 (54%); control n=17 (57%) | Physical activity (6MWT) | Health-related Quality of Life (CAT); Health Behaviors (SNAPPS) | Limited evidence for the effectiveness of self-management support via telephone health-mentoring on physical capacity or self-directed exercise at home | Neutral  No benefits to participating in a telerehab program prior to group based pulmonary rehab |
| telEPOC program | Non-randomised controlled clinical trial[85] | Intervention n=119; control n=78 (n=18, 9.1%) | Intervention 71.3 (9.4); control 70.1 (7.5) | Intervention n=16 (13.4%); control n=10 (12.8%) | Rate of hospitalization for COPD; Visits to ED related to COPD | Quality of life (St George’s Respiratory Questionnaire); Activities of Daily Living (London Chest Activity of Daily Living);  Exercise (6MWT) | After 2 years, both cohorts showed a reduction in rates of hospital admission, but the reduction was significantly higher in the intervention group (1.14 vs 2.33, p <0.001). Significant reductions in ED attendance rate, length of stay and rate of readmission at 30 weeks in the intervention group. Significant between-cohort differences (baseline to 2 years) in ADL's (LCADL), exercise capacity (6MWT), anxiety and BODE index (body mass index, airflow obstruction, dyspnea, exercise capacity). No significant difference in depression or mortality at 2 years. | For COPD patients with frequent admissions, adding a telemonitoring and structured education program to their standard care may reduce their use of healthcare resources related to admissions and ED presentations. Compared to just providing standard care, a program like this may provide benefits in their exercise capacity, ability to participate in daily activities, and their anxiety. |
| COPD telemonitoring service (TELESCOT Program) | Multi-centre randomized controlled trial[86,87]  (baseline and 12 months) | n=128 intervention; n=128 control (n=51, 19.9%) | 69.4 (8.8) intervention; 68.4 (8.4) control | n=75 (59%) intervention; n=65 (51%) control | Time to first hospital admission | Exacerbations of COPD, admissions and deaths; health related quality of life; anxiety and depression (Hospital anxiety and depression scale); participant knowledge (St George’s respiratory questionnaire); self-efficacy (Self-efficacy for managing chronic disease 6 item scale) | The time to admission did not differ between the intervention and control. There was no difference in the mean number of COPD admissions or any other secondary outcome measures. | Neutral  Telemonitoring did not have any effect on delaying hospital admissions in the intervention group but did increase the workload of health professionals. |
|  | Mixed methods study[88] | n=27 patients, n=25 professionals | 68 (8.7) patients | 11 (41%) participants | Perceptions | Not reported | Patients were positive about the technology and perceived it enabled earlier recognition of exacerbations. Health professionals were concerned about false positive symptom score, over-treatment and an increased workload. | Patients were positive but health professionals were apprehensive about the technology. |
| CoMET program | Randomized controlled trial[89]  (baseline, 12 months) | Intervention n= 172; control n=173 (n=54, 15.7%) | Intervention 67.3 (8.9); control 66.6 (9.6) | Intervention n=48, (30.6%); control n=49, (30.2%) | Rate of hospitalization | Physical activity (6MWD); Body mass index, airflow obstruction, dyspnea and exercise (BODE); Anxiety / depression (Hospital Anxiety and Depression Scale); St George’s Respiratory Questionnaire | Distributions of unplanned all-cause hospitalization days were similar between intervention and control, as was the number of patients with exacerbations of COPD. | Neutral  Overall, there was no statistical difference in primary outcome between intervention and control. |
| u-Health service | Cohort analytic (three groups pre and post follow up)[90] | u-Health device n=78; u-Health device and mobile phone n=36; u-Health devices and video service n=30 | 94% > 61 years of age | n=13 (9%) | Knowledge of COPD management (BCKQ) | Attitudes | The study demonstrated that COPD knowledge increased over the course of the study and most participants were satisfied or very satisfied with the program | The monitoring and provision of education to people with COPD can be achieved remotely. |
| Net-COPD | Qualitative study using semi-structured interviews[91] | 14 | 69.5 (55-83) | 8 (57%) | Attitudes | None reported | Participants conveyed satisfaction with the intervention. Three themes; a sense of security and control from self-monitoring, knowing your disease and the virtues of the virtual consultation. | Technology was not a barrier to participation in the program. Participants noted the value of consistency regarding who they had contact with. |
| COPD telehealth program | Randomized controlled trial[92]  (baseline, one month, three months, six months and nine months) | Intervention n=15; control n=14 (n=17, 58.6%) | Intervention 64.1 (9.0); control 62.8 (7.4) | Intervention n=6 (50%); control n=6 (50%) | Adherence to intervention (diaries); satisfaction (Client Satisfaction Questionnaire 8) | Rate of hospitalization; Length of stay; exercise (6MWT); fatigue (Multi-dimensional Fatigue Inventory 20); health status (Clinical COPD Questionnaire); dyspnea (Medical Research Council scale) and Quality of Life (EQQol-5D) | Increased satisfaction with telehealth program and high take up of the telehealth program. Adherence to the exercise program was low. | Neutral  A telehealth program with decision support indicates high satisfaction and higher uptake of some interventions. |
| COPD telerehabilitation program | Cohort (one group pre and post follow up)[93] | 10 participants (n=0, 0%) | 54 (51 to 56.8) | 5 (50) | Rate of hospitalization | Long-term exercise maintenance (COPD Assessment Test, St George’s Respiratory Questionnaire); adherence to exercise program; hospital length of stay, health care costs; quality of life (EQ-5D). | After more than one year, all participants were still participating actively, and no dropouts had occurred. On average, there were 2.0 training sessions/week, 3.3 measurements /week registered via the website and 0.5 videoconference contacts/week. There was a reduction of 27% in the COPD-related hospital costs. Feedback from the participants was very positive. | Long-term telerehabilitation of COPD patients is feasible, and appears promising in reducing the burden for the healthcare system  and patients. |
| Chronic pain | | | | | | | | |
| Chronic Knee Pain  Internet-Delivered Exercise and Pain-Coping Skills  Training Intervention | Randomized controlled trial[94]  (baseline, 3 months and 9 months) | Intervention n=74; control n=74 (n=15, 10.1%) | Intervention 60.8 (6.5); control 61.5 (7.6) | Intervention 43 (58); control 40 (54) | Pain management (Numerical Rating Scale); physical dysfunction (Western Ontario and McMaster Universities Osteoarthritis Index) | Quality of Life (Assessment of Quality of Life instrument); self-efficacy (Arthritis Self-Efficacy Scale); pain catastrophizing (Pain catastrophizing Scale); coping skills to manage pain (Coping Attempts Scale of the Coping Strategies Questionnaire) | Intervention group had significant improvement in self-reported pain, pain while walking, physical function. | Positive  The combination of internet-delivered education and physiotherapy coaching and treatment improved chronic knee pain outcomes. |
| Chronic Low Back Pain  Healthy Lifestyle intervention | Randomized controlled trial[95]  (baseline, 2 weeks and monthly for 6 months) | Intervention n=80; control n=80 (n=36, 22.5%) | Intervention 56.0 (13.3); control 57.4 (13.6) | Intervention n=38 (60.8%); control 46 (57.5%) | Self-reported back pain intensity (Numerical Rating Scale) | Self-reported weight (kg); low back pain disability (Roland Morris Disability Questionnaire); Quality of Life (12-item Short Form Health Survey); Sleep quality (Pittsburgh Sleep Quality Index); physical activity (Active Australia Survey); alcohol consumption (Alcohol Use Disorders Identification Test); smoking prevalence; Pain Attitudes; health care utilization; emotional distress (Depression Anxiety Stress Scale) | No difference between groups for pain intensity over 6 months or any secondary outcome.  In the intervention group, 41% of participants reported an adverse event compared with 56% in the control group. | Neutral  Results showed that a healthy lifestyle intervention involving brief telephone advice, offer of a clinical consultation involving detailed education, and referral to a 6-month telephone-based healthy lifestyle coaching service targeting weight loss, physical activity, and diet did not improve pain intensity for patients with low back pain who were overweight or obese.  The intervention did not reduce self-reported weight, the hypothesized mechanism to influence pain, nor did the intervention improve other secondary outcomes. |
| Diabetes (including Gestational Diabetes) | | | | | | | | |
| Mobile telehealth diabetes care | Mixed methods process evaluation of RCT[96] | Intervention n = 45; control n = 36  (n = 10), 12.3%) | Not reported | Not reported | Feasibility | N/A | Enrolled sample represented 6% of clinic patients. During intervention period, there were 263 contacts between nurses and participants, with text-messaging being the most frequent type of contact used. | Recruiting from clinics to mobile telehealth interventions can be challenging and subject to contextual variables. A factor directly mediating with intervention implementation, included nursing staff turnover. |
|  | Randomized controlled trial including qualitative data[97,98]  (baseline, 3 months and 9 months) | As above | Intervention 58.2 (13.6); control (55.8 (13.8) | Intervention n=14 (31.1%); control n=21 (58.3%) | HbA1c; Self-efficacy (HeiQ, insulin management diabetes self-efficacy scale) | Illness beliefs (personal models of diabetes scale); Diabetes self-care; perceptions; BP, daily insulin does | The program did not significantly influence HbA1c (p=0.228). Significant effects were found for four of the seven self-efficacy subscales in the intervention. Qualitative themes included increased awareness, increased motivation, influence on diabetes self-care and perceived sense of security. | Neutral  The program did not achieve clinically significant changes in HbA1c but has potential to empower people with diabetes to self-manager their condition. |
| PEACH Trial | Pragmatic cluster randomized controlled trial[99]  (baseline, 12 months and 18 months) | Intervention n=236; control n=237 (n=45, 9.5%) | Intervention 63.6 (10.4); control 61.9 (10.5) | Intervention n=109 (46%); control n=95 (40%) | HbA1c | Lipid profile; renal function; BP; BMI; waist circumference; smoking status; quality of life; diabetes self-efficacy; diabetes support; depression status; intensification of treatment | At 18 months follow up, the effect on HbA1c did not differ between the intervention and control. Other biochemical and clinical outcomes were similar between groups too. | Neutral  practice nurse led telephone coaching program for people with diabetes in the primary care setting, produces comparable outcomes. |
| A tele-education intervention | Randomized controlled trial[100]  (baseline, 3 months, 6 months, 12 months and 24 months) | Intervention n=81; control n=72  (n= 19, 12.4%) | Intervention 37 (14.7) Control 38 (13.2) | Intervention 38 (47)  Control 38 (53) | HbA1c | Fear of Hypoglycemia Survey; self-efficacy (Confidence in Diabetes Self-Care); diabetes distress (Problem Areas in Diabetes) | Participants in both groups (received tele-education at different time points) demonstrated significant improvements in glycemic control which were sustained. | Positive  Implementation of tele-education between in-person contacts improved glycemic control for both groups, which was maintained over two years. |
| Telehealth diabetes self-management education | Randomized controlled trial[101]  (baseline and 9 months) | Intervention n=26  Control n=21  (n=27, 36.4%) | Intervention 52  Control: 49  (SDs not reported) | Intervention: 18 (69)  Control: 9 (43) | HbA1c | Blood pressure  Weight loss (weight, waist circumference) | Intervention group reported increased knowledge of diabetes and adherence to self-management practices. Significant association between the intervention and reaching target HBA1c =< 7% (p < .05). Significant association between intervention and decreased BMI 18.5-24.9 (p < .05). | Positive  A telehealth intervention for low-income, urban people, can improve HbA1c and increase knowledge of diabetes self-management. |
| TeleDiab 1 Study (Diabeo Software) | Multicenter randomized controlled trial[102]  (baseline, 3 months and 6 months) | Intervention (G2 with face to face follow up) n=60; Intervention (G3 with teleconsultation follow up) n=59; Control (G1) n=61  (n=7, 3.8%) | Intervention (G2) 32.9 (11.7); Intervention (G3) 31.6 (12.5); control (G1) 36.8 (14.1) | Intervention (G2) n=37 (61.7%); Intervention (G3) n=37 (62.7%); control (G1) n=40 (65.6%) | HbA1c | Quality of life (Diabetes Health Profile and Diabetes QoL questionnaires) | At six months, mean HbA1c was lower in G3 intervention group when compared to the control (8.41 v. 9.10). The Diabeo software resulted in a 0.91% improvement in HbA1c over controls and a 0.67% reduction when used without teleconsultation. | Positive  Telemedicine can result in significant improvements in HbA1c for persons with poorly controlled type 1 diabetes. |
| LINE – diabetes self-management support | Cohort (two or more groups)[103] | n= 49 mobile-based group  n= 91 telephone group  n=91 usual care group  Attrition not reported | 58.6 (6) mobile/ intervention  64.7 (8.3) telephone  64.7 (9.5) usual care | n=17 (35%) mobile/ intervention  n=41 (45%) telephone  n=47 (56%) usual care | HbA1c | Depression  Diabetes-related distress (PAID Scale) | Participants in the mobile-based group had significant reductions in HbA1c and in diabetes-related distress, when compared with telephone and usual care groups. Participants aged <60 and with higher education levels in the mobile-based group at baseline experienced the greatest improvements inHbA1c and distress levels. | Mobile-based intervention effects on HbA1c and diabetes distress were greater than those in the telephone group and usual care. |
| Modified Diabetes Prevention Program (DPP) | Observational study (intention to treat)[104] | n=191 Rural-16 intervention  n=198 Rural-12 intervention  n=278 Urban-16 control  n=35 (18%) Rural-16  n=29 (15%) Rural-12  n=41 (15%) Urban-16 | 55.3 (12.6) Rural-16 intervention  51.8 (13.1)  Rural-12 intervention  55.7 (12.4)  Urban-16 control | n=168 (88%)  Rural-16 intervention  n=171 (86.4%)  Rural-12 intervention  n=231 (83.1%)  Urban-16 control | Weight loss | Meeting nutritional goals  Meeting physical activity goals | Similar weight loss results for the Urban (in-person) group as the Rural (telehealth) groups. Higher participation, male sex, a lower baseline BMI, monitoring fat intake and physical activity were all associated with achieving weight loss goals. A subset of participants across the three interventions achieved their weight loss goals after the intervention indicating that longer programs may be required for some participants. | The telehealth and in-person groups achieved similar results in terms of weight loss and meeting diet and physical activity goals. |
| Telehealth diabetes self-management education | Randomized controlled trial[105]  (baseline, 6 months and 12 months) | Intervention n=85; control n=80 (n=29, 17.6%) | Intervention 59.9 (9.4); control 59.2 (9.3) | Intervention n=62 (72.9); control n=61 (76.3) | HbA1c | Cholesterol  Blood pressure | HbA1c and LDL cholesterol improvement significantly better in intervention group, when compared to control. No difference in systolic or diastolic BP, BMI, waist circumference, albumin creatinine ratio. Good retention rate (82.4% at the 12-month mark). | Positive  Multicomponent telehealth strategies were effectively utilized to successfully conduct remote-delivery of diabetes self-management education in a rural, underserved, and ethnically diverse primary care setting. |
| Proactive Interdisciplinary Self-Management (PRISMA) Program | Randomized controlled trial[106]  (baseline and 6 months) | Intervention n=101; control n=102  (n=37, 18.2%) | Intervention 69.7(9.8); control 70.1(10.1) | Intervention n=39(41); control n=38(38.8) | Feasibility (platform usage / participation) | N/A | No difference in engagement in online management tool between control and intervention groups | Neutral  PRISMA group education (in person) has limited impact on engagement with e-Vita platform for people with diabetes |
| Living Well with Diabetes (LWWD) | Randomized controlled trial [107,108]  (baseline, 6 months, 18 months and 24 months) | Intervention n=151; control n=151  (n=91, 30.1) | Intervention 57.7(8.1); control 58.3(9) All 58(8.6) | Intervention n=67 (44.4); control n=65 (43) | Weight loss (weight, waist circumference) | Dietary intake (habits)  Blood pressure  Physical  activity  accelerometer derived MVPA*  HbA₁C  Fasting blood lipids | At the end of 18-month intervention there were statistically significant but clinically modest benefits observed for weight loss (p=0.013), waist circumference (p=0.007) mod-vigorous physical activity (p=0.018) and diet quality (p=0.014) in the telephone counseling group compared to control. | Neutral  The program resulted in modest improvements in weight loss and behavior change (diet and physical activity). Lack of improvement in cardiometabolic markers. Limited utility and sustainability of this approach in management of type 2 diabetes |
|  |  | As above | Low call 57.1 (7.3) Medium call 59.4 (7.4) High call 56.8 (9.3) | As above | Weight loss (weight, waist circumference) | Physical activity HbA1c | People with pre-existing anxiety / depression less likely to complete the intervention. Those that had higher call rate (higher dose/ engagement) lost more weight. | Engagement with people with anxiety /depression is limited in telephone coaching. Higher dose rate produced better results in terms of weight loss |
| Medical Guard Diabetes (MGD) system for people with type 1 diabetes | Randomized controlled trial[109]  (baseline and monthly for 6 months) | Intervention n=78; control n=76  (n=36, 23.4) | Intervention 32.2(10.1); control 31.5(9) | Intervention n=45 (57.6); control n=40 (52.6) | Cost | HbA1c  Diabetes knowledge (via Diabetes Knowledge Questionnaire)  Quality of life (via the Spanish Diabetes Quality of Life test)  Self-care treatment adherence (via the Diabetes Self-Care Inventory) | For glycemic control, there were no significant differences in outcomes between control and intervention groups. Intervention more cost-effective and time-efficient for participants. QoL improvement greater in control group than in intervention group | Neutral  Mix of face to face and online monitoring/education is as effective as all face to face in management of adults with poorly controlled Type 1 diabetes, yet is significantly more time and cost-effective for patients |
| eTelemonitoring diabetes management | Randomized controlled trial[110]  (baseline, 3 months and 6 months) | Intervention n=70; control  n=35  (n=10, 8.7%) | Intervention 55.2 (16.1); control  55.4 (19.2) | Intervention n=25 (35.7%); control  n=11 (31.4%) | HbA1c | BMI  Hypo- and hyperglycemia frequency  Cost to participant | Telemonitoring achieved better HbA1c improvement than the control group sustained at six-months. This was attenuated at the six-month post intervention mark, indicating that improvement may be linked to more frequent contact with healthcare professional.  Participants with higher HbA1c at baseline (>10%) experienced greater improvement. | Positive  Statistically significant reduction in HbA1c in telemonitoring versus control group and less frequent hypo- and hyperglycemic episodes. |
| Telehealth remote monitoring (Care Innovations Guide) | Randomized controlled trial[111]  (baseline and 6 months) | Intervention n= 45; control  n= 45  (n=9, 10%) | Intervention 53.9 (10.4); control  57.5 (10.6) | Intervention n=23 (51.1%); control  n=19 (42.2%) | HbA1c | Medication changes  Empowerment (measured via the DES)  Diabetes knowledge (measured via the DKT)  Self-management behaviors: diet, physical activity, SMBG, medication, footcare (measured via the SDSCA) | At 6 months, intervention group demonstrated a statistically significant sustained decreased in HbA1c levels compared with control group. Intervention group self-reported more medication changes at 3 and 6-month marks (i.e. reduced clinical inertia) at a statistically significant level compared with control group. | Positive  Intervention group achieved a statistically significant reduction in HbA1c at the six-month mark compared with the control group. |
| Telescot | Qualitative study of RCT participants and healthcare providers[112] | n=23 patient participants  n=10 healthcare professional participants | N/A | N/A | Acceptability and perceptions | Not reported | Telemonitoring of blood glucose and BP was deemed to be feasible, well-accepted by patients and was effective in supporting self-care and medical treatment decision. | Telemedicine was understood to be feasible and well accepted. |
|  | Randomized controlled trial[113]  (baseline and 9 months) | Intervention n=160; control n=161  (n=36, 11.2%) | Total 61 (9.8) | Intervention n=54 (33.8%); control n=53 (32.9%) | HbA1c | BP; Weight | There was a clinically and statistically significant improvements in the telemonitoring group over 9 months when compared to the control/usual care group. There were also significant reductions in blood pressure.  No statistically significant differences in weight loss between the groups. | Positive  Telemonitoring group achieved greater improvement to HbA1c and BP levels than the control group. |
| IDEATel project | Randomized controlled trial[114]  (baseline, 12 months and 24 months) | Intervention n=447  Control n=443  (n=158, 17.8 at 1-year; n=452, 50.8 at 2-year) | All 71.02(7.07) | All 507 (57) | Weight loss (weight, waist circumference) | Dietary intake  Physical activity | Intervention group showed longitudinal benefits on diet and exercise. Intervention appears to have improved participant knowledge of appropriate diet and exercise behaviors. | Positive  Telehealth education and case management led to enhance knowledge (diet and exercise) and led to improvements in waist circumference and BMI. |
|  | Five-year results from randomized controlled trial[11,116]  (baseline and 5 years) | n=844 intervention  n=821 control group  n=248 (14.9%) | White 71.3 (6.95)  Black 70.7 (7.06)  Hispanic 70.3 (5.88) | White n=455 (55.54)  Black n=185(74.6)  Hispanic n=400 (68.38) | Self-care behaviors (measured via SDSCA)  HbA1c | Comorbidities  Diabetes-related symptoms (measured via Type 2 Diabetes Symptom Checklist); Weight loss (weight, waist circumference) | Intervention improved adherence to diabetes self-care behaviors and led to improved glycemic management in intervention group when compared to control group (to a statistically significant level). All three ethnic groups improved adherence to self-care. Over time, lower A1C levels were associated with more glucose uploads and female sex. | Positive  Self-care behaviors and HbA1c levels achieved by telehealth group were better than those of the control group. The IDEATel intervention was associated with improvement in glycemic control, more so in Hispanics, suggesting that telemedicine can help reduce disparities in diabetes management. |
|  | Randomized controlled trial[117]  (baseline and five years) | Intervention n=837  Control n=813 | Intervention 70.8(6.5)  Control 70.9(6.8) | Intervention n=532 (63.6)  Control n=505 (62.1) | Physical activity | Not reported | Study showed less decline in physical activity and lower rate of physical impairment in the intervention group (Control 2.23 points compared with intervention 1.64 points: p=0.037). Pedometer use was associated with preserved functional status in older people with diabetes | Positive  Telemedicine intervention with older people can significantly lessen the rate of decline in PA and PI over 5 years. Greater PA associated with fewer comorbid conditions, less depression, more social networking, lower BMI, male gender and lower hemoglobin A1c at baseline. |
|  | Randomized controlled trial[118] | Intervention n=610 (61, 10%) | Intervention  Age 55–64 14.4%  Age 65–69 32.3%  Age 70–74 24.4%  >80 12.3% | Intervention n=336 (55.1) | Goals set (goals attained) | Not reported | Overall, 68% of behavioral goals were rated as ‘‘improved’’ or ‘‘met.’’ The most common goals were related to monitoring, followed by diabetes health maintenance, nutrition, exercise, and use of the telemedicine equipment.  The greatest success was achieved for goals related to proper insulin injection technique and daily foot care. | Positive  Collaborative behavior change goals can be set and attained using telehealth appointments. Telehealth can improve diabetes self-management in underserved elderly rural adults. |
| Group diabetes self-management education delivered via telehealth to participants at a rural health service site | Cohort analytic (two group pre and post)[119] | Intervention n=27; control n=39 | Intervention 49.8 (11.5); control 54.9 (10.9) | Intervention n=19 (70.4); control n=22 (56.4) | HbA1c | Anxiety /depression | HbA1c changed from 8.2(0.31) at baseline to 7.8(0.28) at 6 months in face to face group compared to 7.8(0.29) at baseline to 7.3(0.37) at 6 months in telemedicine group. There were no significant changes in weight between groups or within groups. Significant improvements in each group with PAID score and improvement in DTSQ (satisfaction with Diabetes treatment) in face to face group only | There was no statistical difference between face to face and telemedicine. Challenges included need to have a good understanding of telemedicine equipment, troubleshoot technical problems and need for a dedicated telemedicine health care provider |
| Remote delivery specialty clinic model | Randomized controlled trial[120]  (baseline and 12 months) | Intervention n=30; control n=30  (n=16, 26.7) | Intervention 54.4 (9.6); control 54.3 (9.8) | Intervention 15(50); control  13(43.3) | HbA1c | Weight (BMI)  Statin treatment  Blood pressure | HbA1c decreased in intervention group by 1.7% (statistically significant), whereas in control group decreased by 0.3% (not significant). Statin therapy usage was greater in the intervention group. Blood pressure and BMI changes were comparable in both groups | Positive  Greater frequency of interactions between patients and providers, as demonstrated through intervention using email and telephone contact, the greater the clinical benefits when compared to less frequent contact |
| Mobile Phone  and Web-Based Collaborative Care  Intervention  for Patients with Type 2 Diabetes | Qualitative study[121] | Intervention  n=8  Control n=6 (not involved in this study) | Not reported | Not reported | Feasibility | N/A | Five major themes emerged; (1) connecting with the nurse practitioner is valuable, (2) uploading data from glucose meters is easy, (3) smartphones are frustrating, (4) program helps me focus on taking care of myself, (5) accessing the web features through the Wii was not useful. | Participants generally appreciated increased accessibility of nurse practitioner through internet, but participants can experience frustrations with technology |
| Rural telemedicine and DSME program (provided in-person) | Retrospective cohort study[122] | Intervention n=63; (n=4, 6.3%) | 21-76 | 47 (80) | Feasibility | HbA1c  Rate of hospitalization  LDL cholesterol  Blood Pressure  Endocrinology appointments | Participants were either satisfied or very satisfied with the use of VC telemedicine (endocrinologist) and the instruction for medication and education. All reported they could explain medical problems using telemedicine, were satisfied, found it convenient and would recommend to others. HbA1c reduction was achieved (stat sig. mean 0.83%) and there was decreased rate of hospitalization of program participants | Telehealth education and case management led to enhance knowledge (diet and exercise) and led to improvements in waist circumference and BMI for older people in an underserved community. |
| SINERGIA program | Cohort (one group pre and post follow up)[123] | 1004 participants | 66.6 (9.5) | n=461 (45.9%) | HbA1c | Blood pressure; LDL cholesterol | Proportion of patients with a HbA1c <7.0% increased from 32.7% to 45.8%. Improvements were also noted in other clinical indicators (LDL) | The program has potential to improve metabolic control in the medium term. Support for use of telemedicine to facilitate interaction between patients and clinicians was given. |
| Smartphone-based health coaching | Qualitative study[124] | n=11 (out of 22 in the intervention group) | Females 55.8(8.8) Males 63.5(4.9) | 9 (82) | Attitudes | Feasibility | The key findings of the study reports that the smartphone-based behavior monitoring software helped individuals track behavior and communicate with their health coach and adapt an active role in self-management. The overall intervention was most effective when software use was optimally coordinated with personalized health coaching interactions. | From a patient perspective, smartphone monitoring software substantially enhanced the therapeutic alliance with patients who held their intervening coaches in high regard |
| Phone-based motivational interviewing for women with previous GDM | Pilot randomized controlled trial[125]  (baseline and 6 months) | Intervention n=18; control n=20  (n=6, 15.8%) | Intervention 32.9(4.9); control 32.2(5.1) | n=38 (100%) | Weight loss (weight, waist circumference) | Dietary intake (habits)  Physical activity (steps, sedentary time, 6MW) | There was 1 kilogram weight reduction in the intervention group and a 3 kg increase in the control group between baseline and follow-up (regression coefficient: -4.0kg; 95%CI: -7.6 to -0.5), and significant improvements in BMI in the intervention group compared to the control group (regression coefficient: -1.5 kg/m2  ; 95%CI: -2.8 to -0.1). | Positive  The lifestyle intervention delivered via telephone may improve weight status, physical activity and dietary behavior in women with a recent history of GDM |
| Web-based education and self-management platform for people with diabetes | Cohort (one group pre and post)[126] | Intervention n=21 | 54.36 (4.09) | n=14 (66.7%) | HbA1c | Cholesterol  Blood pressure | Statistical and clinical changes in HbA1c; cholesterol improvements. No change in BMI. Higher SF-36 scores indicating improved satisfaction. | Resource intensive approach with some positive results. A less expensive approach is needed for those with moderately controlled diabetes. |
| Telemedicine for diabetes care | Cluster randomized controlled trial[127]  (baseline and 18 months) | Intervention n=199; control  n=83  Attrition not reported | Intervention 61.6 (9.4); control  61.1 (10) | n=1 (0.36%) | HbA1c | Satisfaction (measured via the DTSQ)  BP  Cholesterol  Creatinine | Participants in both the intervention/telehealth and control/usual care groups achieved a small decrease in the HbA1c with no statistical significance between the groups. | Neutral  Similar outcomes in terms of HbA1c improvement |
| Diabetes Prevention Program (DPP) | Cohort analytic (two groups pre and post)[128] | Intervention (telehealth) n=14; control (on-site) n=13 | Intervention 50(7); control  53(14) | Intervention n=13 (93%); control n=9 (69) | Goals set (goals attained) | Physical activity  Weight loss (weight, waist circumference) | No statistical difference in program participation, weekly fat goals or physical activity between telehealth and on-site group | Similar outcomes can be achieved regardless whether programs are implemented on site or through telehealth. Authors deemed the program to be feasible |
| Pedometer-based physical activity intervention with telephone mediated motivational interviewing | Randomized controlled trial[129]  (baseline, six months and 12 months) | Intervention n=60; control n=32  (n=4, 4.3%) | Total 62 (9) | Total 31% | Physical activity | HbA1c  Blood pressure | Intervention group increased their physical activity (steps per day) more than the control, however there were no significant changes in health outcomes. An increase of >4000 steps a day significantly improved HbA1c (-0.2%), whereas an increase in steps by <4000, led to an increase (0.3%) in HbA1c. | Neutral  Despite its clear effects on physical activity, this pedometer-based behavioral intervention had no direct effects on any of the measured health outcomes in this generally well-controlled group of type 2 diabetes patients. |
| Telephone diabetes self-management education and coaching | Randomized controlled trial[130]  (baseline, six months and 12 months) | Intervention n=47; control n=47  (n=23, 24.5%) | Intervention 59 (56-62); control 64 (10.7-15.6) | Intervention 13 (28%); control 17 (36%) | HbA1c | Blood pressure  Weight loss (weight, waist circumference)  Fasting blood glucose levels  Fasting cholesterol  Fasting triglycerides  Physical activity  Depression/ anxiety (via K10) | Significant interaction effects were observed between groups at 6 months, demonstrating improvement in HbA1C, fasting glucose, diastolic blood pressure and physical activity. The intervention’s effect on these parameters was not sustained at 12 months. | Positive  Telephone coaching improved glycemic control and adherence to complication screening in people with type 2 diabetes, for the duration of its delivery, but these effects were not maintained on withdrawal of the intervention. |
| Telephone delivered diabetes self-management education | Cohort analytic (two groups pre and post)[131] | Intervention n=633; control n=598  (160, 20.2) | Intervention 63(12.5); control 61.8(12.8) | Intervention 296 (47.8%); control 276 (46.2%) | HbA1c | Rate of hospitalization  Blood pressure | HbA1c levels were significantly lower in intervention group when compared to usual care, both before and after the program. | Self-management education delivered via telephone by trained nurses between physician follow-up visits, can improve HbA1c. |
| Home telehealth for diabetes and hypertension | Randomized controlled trial[132]  (baseline and 12 months) | Intervention (high) n=93; intervention (low) n=102; control n=107  (n=56, 18.5%) | Intervention (high) 67.8 (10); intervention (low) 68.4 (9.5); control  67.9 (9.9) | Intervention (high) n=1 (1%), intervention (low) n=1 (1%); control n=4 (3.7%) | HbA1c | BP  Depressive symptoms (measured via the GDS)  Adherence to self-care (measured via Self-Reported Medication Taking Scale and a validated scale for diabetes self-care) | Significantly greater decrease in HbA1c in the intervention groups (high and low) compared with control group at the six-month mark. At the 12-month mark, the control group showed a significant reduction in HbA1c where the low and high intensity intervention groups did not. Adherence to self-care improved over time in all three groups (no stat sig. differences). | Neutral  Changes were not sustained beyond the intervention (12-month mark) |
| Telehealth-delivered diabetes self-management education and support | Feasibility study[133] | Intervention n= 20  (n=8,40%) | 66.5 (8.62) | n=11 (55%) | Feasibility  (proportion of patients approached and enrolled versus declined) | Adherence | For the 12 patients who completed the program, general diabetes knowledge significantly improved, as did knowledge of insulin. HbA1C level also improved.  At 30 days, two individuals who did not complete the program had rehospitalizations.  Notably, there were no unplanned hospital readmissions for any patient who completed the program during this same timeframe. | It is feasible to identify and enroll patients in a telehealth education program for diabetes during hospital admission. Diabetes knowledge (including medication) and HbA1c can be improved by a telehealth intervention. Hospitalization rate can also be decreased |
| Risk factors for chronic disease | | | | | | | | |
| Overweight/obese  MOVE! Program | Retrospective cohort study – two groups[134] | n=60 intervention; n=60 control | 57 (10.1) intervention; 62 (11.1) control | n=5 (8%) intervention; n=3 (5%) control | BMI; weight change | N/A | The intervention group receiving the MOVE! Program lost weight, whereas the control group gained weight. The mean difference between groups was -5.5 ± 2.7 kg (95% CI = -8.0 to -3.0; P<0.0001). | Provides evidence for the use of videoconferencing to deliver the MOVE! Weight management program to veterans. |
| Physical inactivity  Healthy4U | Economic Evaluation of a randomized controlled trial[135]  (baseline and 6 months) | Intervention n=38; control n=36 (n=4, 5.6%) | 53 (8) | n=54, 75% | Change in moderate-to-vigorous physical activity (MVPA, accelerometry); Resource use and costs | HRQoL (SF-12, standard Brazier algorithm) | The results are reported as the mean cost for the program and mean change in (1) moderate or vigorous physical activity and (2) quality of life (i.e. incremental cost-effectiveness ratios). Over the follow up period, the intervention group experienced higher increases in physical activity and HRQoL than the control group. Total est. cost of delivering the intervention was $279 p/p which led to an average of 41min (12) of MVPA per day. The intervention would cost approx. $37,000 for each QALY gained. | Positive  Telephone-delivered MI-CBT is effective in increasing PA and QoL in people with and without chronic disease. There is a cost associated and the cost-effectiveness of the intervention depends on the willingness to pay for each QALY. |
| Psychological distress prevention  Telephone and web-based coping skills training program | Randomized controlled trial[136]  (baseline, 3 months and 6 months) | Telephone intervention patients n=86, family members n=39; education program patients n=89, family members n=47 (patients n=44, 25.1%; family members n=20, 23.3%) | Telephone intervention patients 49.7 (13.8), family members 50.0 (14.9); education program patients 53.7 (13.5), family edu 52.9 (15.2) | Telephone intervention patients n=38 (44), family members n=33 (85); education program patients n=37 (42), family members n= 36 (77) | Psychological distress (HADs) | Impact of events (Impact of Events Scale-Revised); PTSD (PTSD symptom score), Health related Quality of Life (EQ-5D); global mental and physical health status (PROMIS); adaptive coping behaviors (COPE) | No significant difference between telephone intervention and education program in either 3-month HADS scores (difference, 1.3; 95% confidence interval [CI],20.9 to 3.4; P = 0.24) or secondary patient and family outcomes. In patients with high baseline distress (n = 60), telephone intervention recipients had greater improvement in 6-month HADS score (difference, 24.6; 95% CI, 28.6 to 20.6; P= 0.02) than the education group. | Neutral  The telephone intervention did not improve psychological distress symptoms compared with an education the telephone intervention  improved symptoms of distress at 6 months among patients with  high baseline distress, whereas the education program improved  distress at 3 months among those ventilated for more than 7 days. |
| Overweight  Lifestyle, Eating and Activity Program (LEAP Beep) | Cohort analytic (two groups, pre and post)[137] | Intervention n=17; control n=17 | Intervention 58.3 (12.1); control 59.1 (9.5) | Intervention n= 10 (59%); control n= 13 (76%) | Weight loss (weight, waist circumference); BMI | QoL (Impact of Weight on Quality of Life tool IWQOL); anxiety/depression (Hospital Anxiety and Depression Scale) | Following the intervention a significant difference was found between groups for body weight, waist circumference, BMI.  Both groups experienced a reduction in anxiety, however no difference was found between groups. | Text messaging may be a cost-effective and convenient way to support behavior changes, goal setting and self-monitoring in obese patients, and may support weight loss. |
| Obesity  GLOW study | Randomized controlled trial[138] | Intervention n=200; control n=198 (n=4, 1%) | Intervention 32.4 (4.1); control 32.6 (4.3) | Total n=394 (100%) | Weight loss (weight, waist circumference) | Dietary intake (habits) | Compared with usual care, women in the lifestyle intervention had a significantly reduced weekly rate of GWG (mean 0·26 kg per week [SD 0·15] vs 0·32 kg per week [0·13]; mean between-group difference –0·07 kg per week, 95% CI –0·09 to –0·04). | Positive  A lifestyle intervention delivered primarily by telehealth was feasible in health-care delivery settings and significantly reduced the proportion of women exceeding guidelines. |
| Obesity  Mobile phone app for patients who are overweight or obese | Cohort (one group pre and post follow up)[139] | n=43 (n=7, 16.2%) | 40.6 (12.4) | n=36 (83.7%) | Weight loss (weight, waist circumference, anthropometric and clinical assessments) | Dietary intake (habits); fasting glucose; lipids; blood pressure; body composition; physical activity (Global Physical Activity Questionnaire); Quality of Life (SF-12) | The median weight change after the first 12 weeks was −3.8 kg (range: −15 to 2.4 and P<.001), between week 12 and week 52 it was −1.1 kg (range: −9.7 to 7 and P=.08), and the median change during the entire period of intervention was −4.9 kg (range: −21.9 to 7.5 and P<.001). Changes in BMI, waist circumference, body fat, and BP between baseline and 12 weeks and between baseline and 52 weeks were also significant. | The results show that this form of behavior counseling leads to significant weight loss, both in the first 3 months during an intensive remote counseling period, as well as weight loss (not significant). |
| Hypertension  Self-Help Intervention Program for High Blood Pressure (SHIP-HBP) | Randomized controlled trial[140]  (baseline, 3 months and 15 months) | Intensive counseling group n=203; less intensive counseling group n=194 (n=37, 9.3%) | Intensive counseling 51.8 (5.7); less intensive counselling 51.9 (5.8) | Intensive counseling n=95 52.7%; less intensive counselling n=94 52.8% | Intervention completion | Health behaviors (medication compliance, smoking, alcohol consumption, exercise) | For the patients who received the nurse telephone counseling, 360 (90.7%) completed the intervention. Over the 12-month counseling period, a total of 11,415 phone calls were placed; 5090 calls of which were successful. Success rates of telephone outreach were 74.2% (about 18 calls per person) for the MI and 86.5% (about 10 calls per person) for the LI groups (p < 0.001). | The success of telephone outreach was influenced by the dose of the intervention, the participant’s employment status, and the number of years of residence. |
| Obesity  Self-management and educational support in severely obese patients (EVOLUTION) | Randomized controlled trial[141]  (baseline, 3 months, 6 months and 9 months) | In-person intervention n=215; web-based intervention n=225: control group n=211 (n=188, 28.9%) | In-person intervention 40.5 (9.9); web-based intervention 40.6 (10.1); control group 40.4 (9.3) | In-person intervention n=176 (81%); web-based intervention n=183 (81); control group n=181 (86)) | Proportion of patients achieving weight loss (weight, waist circumference, BMI) | Blood pressure; HbA1C; lipids; Health-related quality of life (SF-12, EQ-5D, Patient Satisfaction Questionnaire); self-efficacy (Weight Efficacy Life-Style Questionnaire); depression (Patient Health Questionnaire); readiness to change (visual analogue scale); costs | At nine months, the participants enrolled in the web-based arm reported the highest proportion of 5% weight loss (24.9%). However, no statistically significant difference in the 5% weight loss in the three groups (web-based, in-person, controls) was found. | Neutral  The trial concluded that intensive in person intervention or web-based self-management interventions were no more effective that the provision of printed educational materials for patients with severe obesity. |
| Obesity  Interactive Diary for Diet Management (DAI) | Cohort (one group pre and post follow up)[142] | 140 (n=24, 17.1%) | 42.9 (12.2) | n=68 (58.3) | Weight loss (weight, waist circumference) | Dietary intake (habits); lipid profile; fasting blood glucose | Significant reduction body weight of 2.5kg and 68% reducing weight. Reduced waist circumference and BMI. Reduced calorie intake. Increased uptake of local fresh food. | Intervention effective in promoting healthy eating and the consumption of local produce. |
| Stroke prevention  Telehealth Stroke Education | Quasi-experimental non-equivalent control group design[143] | Telehealth n=9; in person n=10 (n=8, 42.1%) | Telehealth 71 (64-75); in person 69 (65-76) | Telehealth n=2 (33%); In person n=5 (100%) | Attitudes | Behavior change | No significant difference in satisfaction, knowledge and likelihood of taking preventative action against stroke by adults receiving intervention either in-person or through videoconferencing. | There was no difference in feedback from participants when a stroke prevention education program is deliver in-person or via videoconferencing. |
| Lifestyle prevention  SHINE (Diabetes Prevention Program) study | Randomized controlled trial[144]  (baseline, 6 months, 12 months and 24 months) | Conference Call (CC) intervention n=128; individual call (IC) intervention n=129 (n=122, 47.5%) | CC intervention 52.7 (12.8); IC intervention 50.7 (13.1) | CC intervention n=92 (71.9); IC intervention n=101 (78.3%) | Weight loss (weight, waist circumference) | Blood pressure; fasting glucose; lipids | There was similar weight loss at year 1. Between year 1 and year 2 Conference Call (CC) participants continued to lose weight while Individual Call (IC) participants regained.  At year 2, 52 % and 43 % (CC) and 29 % and 22 % (IC) of participants lost at least 5 % and 7 % of initial weight | Neutral  Primary Care Provider delivery of the Diabetes Prevention Program lifestyle intervention by telephone can be effective in achieving weight loss in obese people with metabolic syndrome. |
